# GC-MS-based metabolomics of volatile organic compounds in exhaled breath: applications in health and disease. A review

**DOI:** 10.3389/fmolb.2023.1295955

**Published:** 2024-01-08

**Authors:** María Bajo-Fernández, Érica A. Souza-Silva, Coral Barbas, Ma Fernanda Rey-Stolle, Antonia García

**Affiliations:** ^1^ Centro de Metabolómica y Bioanálisis (CEMBIO), Facultad de Farmacia, Universidad San Pablo-CEU, CEU Universities, Urbanización Montepríncipe, Boadilla del Monte, Spain; ^2^ Departmento de Química, Universidade Federal de São Paulo (UNIFESP), Diadema, Brazil

**Keywords:** volatile organic compounds, exhaled breath, breath test, gas chromatography-mass spectrometry, biomarkers

## Abstract

Exhaled breath analysis, with particular emphasis on volatile organic compounds, represents a growing area of clinical research due to its obvious advantages over other diagnostic tests. Numerous pathologies have been extensively investigated for the identification of specific biomarkers in exhalates through metabolomics. However, the transference of breath tests to clinics remains limited, mainly due to deficiency in methodological standardization. Critical steps include the selection of breath sample types, collection devices, and enrichment techniques. GC-MS is the reference analytical technique for the analysis of volatile organic compounds in exhalates, especially during the biomarker discovery phase in metabolomics. This review comprehensively examines and compares metabolomic studies focusing on cancer, lung diseases, and infectious diseases. In addition to delving into the experimental designs reported, it also provides a critical discussion of the methodological aspects, ranging from the experimental design and sample collection to the identification of potential pathology-specific biomarkers.

## 1 Introduction

### 1.1 Volatile organic compounds

Volatile organic compounds (VOCs) are small molecules (MW <500 Da) with low boiling points and high vapor pressures at ambient temperature. The profile of VOCs released by an organism is called the volatilome, reflecting the metabolic state and playing essential ecological and regulatory roles (Mansurova et al., 2018; Netzker et al., 2020; Sidorova et al., 2021). In humans, VOCs are released through breath, skin, feces, urine, sweat, and saliva, among others (Drabińska et al., 2021), and their origin can be endogenous and exogenous (Pleil et al., 2013). Microorganism-derived VOCs, which include symbionts, commensals, and pathogens, should be considered endogenous since they play significant roles in human health (De Vos et al., 2022).

### 1.2 Breath test along the history

The origin of the breath test can be traced back to ancient Greece. Hippocrates of Kos (460–370 BC) described specific types of odors associated with physiological imbalance, such as *fetor hepaticus* for liver dysfunction, *fetor oris* for halitosis, the fruity and sweet odor of patients with uncontrolled diabetes, the urine-like smell of kidney failure, and the putrid stench of lung abscess. Paracelsus, in the 16th century, further emphasized the link between “bad” breath and pathology (Fortes et al., 2017).

In the 18th century, Antoine Lavoisier discovered the role of oxygen in combustion and understood the respiratory physiology in animals (Karamanou and Androutsos, 2013). That was the origin of capnography and modern biochemistry. The sensitive detection of VOCs became possible with the introduction of colorimetry in the mid-19th century. Ethanol was isolated from breath by Francis E. Anstie, and acetone was found increased in the breath of diabetes mellitus patients by A. Nebelthau (Phillips, 1992).

Discoveries made in the 20th century are (Amann et al., 2014): mercaptans were detected in the breath of severe liver disease patients by Davidson (1949), connecting them to the *fetor hepaticus* described by Hippocrates of Kos; acetonitrile was detected in the breath of smokers by McKee et al. (1962); methanol was found in human breath (Eriksen and Kulkarni, 1963); volatile fatty acids were reported in patients with cirrhosis (Chen et al., 1970); ammonia was measured spectrometrically by Hunt and Williams (1977); and dimethyl- and trimethylamine were detected in the breath of end-stage renal disease patients (Simenhoff et al., 1977).

The turning point came when Pauling et al. (1971) published a pioneering study using gas–liquid partition chromatography to analyze body fluids and breath to investigate the influence of diet on human microbiota and health. This study detected 250 VOCs in human breath, offering promising prospects for further research in the field.

### 1.3 Breath test and clinical applications

Breath samples are particularly valuable for VOCs analysis. The gaseous fraction contains over 1,000 VOCs, with acetone and isoprene being the most abundant (Kuo et al., 2020; Drabińska et al., 2021).

Breath tests aim to distinguish between healthy and pathological states by analyzing exhaled breath VOC profiles, identifying pathology-specific compounds and elucidating their biochemical origin. Compared to routine diagnostic methods, they offer several advantages: they are non-invasive, cost-effective, and fast and easy to perform, have an unlimited sample size, and can be safely and repeatedly collected (Sharma et al., 2023). Despite their simplicity, to date, just a few tests are used in clinical practice, such as the fractional exhaled nitric oxide (FeNO) test for asthma diagnosis, the ^13^C-urea breath test for *Helicobacter pylori* infection, the hydrogen/methane test to detect lactose and/or fructose intolerance, also to detect small intestine bacterial overgrowth, standard capnography based on monitoring CO_2_ partial pressure levels during anesthesia and intensive care, and the alcohol breath test used by the police (Simrén and Stotzer, 2006; Buszewski et al., 2007, 2013).

Although many studies propose potential biomarkers for various pathologies, the expected clinical application of breath tests has not progressed as expected (Buszewski et al., 2007; Sharma et al., 2023). Additionally, the link between potential biomarkers and specific pathologies is not clear (Haick et al., 2014; Zou et al., 2022).

### 1.4 Major sources of endogenous VOCs

Oxidative stress (OS) and cytochrome P450 (CYP) enzymes are the main sources of endogenous VOCs. OS damages cellular components, such as phospholipids, proteins, and DNA, thus being involved in the development of many pathological conditions such as cancer, inflammation, and aging. Lipid peroxidation, especially of polyunsaturated fatty acids (PUFAs), is a significant source of VOCs. The breakdown of lipid peroxides produces a wide range of compounds, such as alkanes, alkenes, alcohols, aldehydes, carboxylic acids, esters, epoxides, and furans (Calenic et al., 2015; Ratcliffe et al., 2020).

CYP enzymes participate in reactive oxygen species (ROS) generation and lipid peroxidation, affecting the oxidation–reduction balance and OS, therefore also contributing to VOC generation. CYP enzymes are found in various tissues, with higher levels in the liver and enterocytes (Murray et al., 2009; Veith and Moorthy, 2018; Behrendorff, 2021).

### 1.5 VOCs and exhaled breath

**FIGURE 1:**
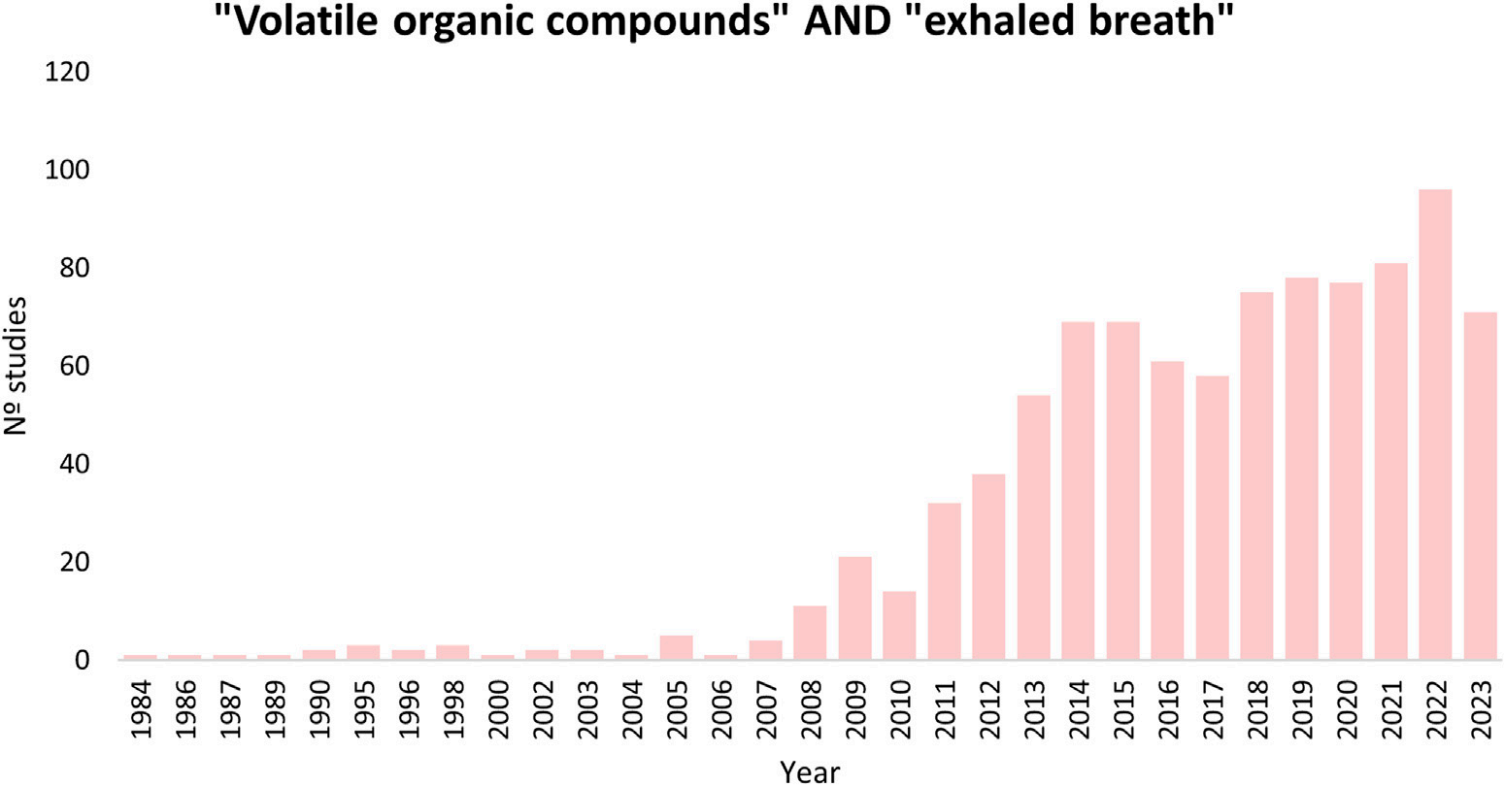
Results searching in PubMed using the terms: (volatile organic compounds) AND (exhaled breath).

As seen in Figure 1, almost 1,000 articles have been published with the aim of finding potential biomarkers and/or therapeutic targets for various pathologies. Lung cancer has been extensively studied (Antoniou et al., 2019; Janssens et al., 2020), although other cancers, pulmonary pathologies [e.g., asthma, chronic obstructive pulmonary disease (COPD), and obstructive sleep apnea (OSA)], gastrointestinal pathologies (e.g., Crohn’s and inflammatory bowel pathologies), diabetes, and infectious diseases (e.g., viral infections, tuberculosis, and invasive aspergillosis) have also been investigated (Sethi et al., 2013; Markar et al., 2015; Van Der Schee et al., 2015; Acharige et al., 2018; Saasa et al., 2018; Hanna et al., 2019; Ghosh et al., 2020; Ratiu et al., 2020). The Human Breathomics Database (HBDB) created by Kuo et al. (2020) is a consequence of the relevance of the topic, gathering information on VOCs detected in healthy and pathological subjects.

### 1.6 Exhaled breath sampling

The average human expiratory volume is 500 mL, comprising three portions: dead space air, air from the airways and alveoli, and alveolar breath. Capnography can monitor the respiratory cycle, as the CO_2_ level shows different trends in each portion. Consequently, breath samples can be classified into three types: mixed breath (containing the three portions), late expiratory breath (excluding dead space air), and alveolar breath (containing only the last portion of the expiration) (Beauchamp and Miekisch, 2020).

There are two types of breath analysis: online and offline. Online provides fast results and allows the volatilome to be monitored with minimal sample manipulation. Nevertheless, offline analysis (storing the sample for subsequent analysis) is the most widely used, as it enables sampling at different locations (Sola-Martínez et al., 2022). Sampling, transport, and storage are critical in the offline analysis of gas samples, since the samples may suffer from possible losses, adsorption, and artifact formation (Alonso and Sanchez, 2013). Therefore, the correct choice of sampling methodology is crucial.

Breath sampling methods can be categorized according to the type of sample collected. Devices employed for mixed breath include containers and bags with a valve system to prevent re-breathing, such as Tedlar^®^ and Mylar bags, sorbent tubes, canisters, sampling tubes/bulbs, and the Pneumopipe device (Pennazza et al., 2014; White and Fowler, 2019). Although these devices are simpler to use, they may lead to losses, diffusion, adsorption onto the sampling device material, and potential contamination, especially with reactive VOCs (Miekisch et al., 2012; Tang et al., 2015; Beale et al., 2016). In particular, Tedlar^®^ bags emit contaminants like N,N-dimethylacetamide, phenol, carbonyl sulfide, and carbon disulfide. To preserve sample integrity, storage time should be minimized, and analysis is recommended within 10 h (Beauchamp et al., 2008; Mochalski et al., 2009).

For collecting late expiratory or alveolar breaths, traditional sampling devices present some adaptations, such as a T-shaped mouthpiece, a spirometer system, and CO_2_ and pressure sensors (Alonso and Sanchez, 2013; Tang et al., 2015). CO_2_ sensors are commonly used for alveolar breath sampling because CO_2_ concentrations are highest and constant in the alveolar phase (Lawal et al., 2017). Various devices are available for this purpose, either collecting a final fixed volume, based on the Haldane–Priestly approach, or using CO_2_ and pressure sensors: BioVOC^®^, RTubeVOC, QuinTron AlveoSampler, ReCIVA, the adaptive breath sampler (ABS), breath collection apparatus (BCA), and SOFIA sampler (Phillips, 1997; Basanta et al., 2007; Beale et al., 2016; White and Fowler, 2019).

### 1.7 Analytical platforms: GC-MS

The concentrations of VOCs in exhalates range from parts-per-million (ppmv) to parts-per-trillion (pptv), requiring highly sensitive analytical techniques to detect these compounds. Analytical platforms used for online and offline analysis include laser spectrometry, selected ion flow tube-mass spectrometry (SIFT-MS), proton transfer reaction-mass spectrometry (PTR-MS), secondary electrospray ionization-mass spectrometry (SESI-MS), ion molecule reaction-mass spectrometry (IMR-MS), and ion mobility spectrometry (IMS). These techniques perform fast analysis and present high sensitivity, although they involve high costs and/or require skilled technicians. An alternative method, also emerging as a point-of-care tool, electronic noses (E-nose) combine selective electronic sensors, offering rapid analysis and affordability. Basically, E-noses are used to detect patterns between the samples, which are further resolved through statistical methods and machine learning. Other online approaches such as optical/laser absorption spectroscopy-based methods detect small molecules with narrow adsorption lines, commonly used for acetone analysis. Additionally, compound identification is limited, and no accepted standards ensure interoperability/normalization of methodologies. A promising approach utilizes nanomaterial-based VOC/gas sensors, which offers a wider dynamic detection range and high selectivity; however, some challenges include receptor immobilization compromising functionality, potentially irreversible reactions between VOCs and the receptor (due to high selectivity), and a reduced likelihood of VOC–receptor interaction due to the small surface area of nanoscale elements (Buszewski et al., 2013; Bruderer et al., 2019; Wojnowski et al., 2019; Sharma et al., 2023).

GC-MS is a mature technique that is considered the “gold standard” for VOC analysis in exhaled breath (De Lacy Costello et al., 2014; Drabińska et al., 2021). It offers high sensitivity and reproducibility, and the ability to identify and elucidate unknown compounds, especially with high-resolution instruments (Sola-Martínez et al., 2022; Sharma et al., 2023). In addition to requiring an offline approach such as a pre-concentration step, GC-MS applicability may be hampered by its high costs, complex and time-consuming sampling, requirement for standardization and trained personnel, and inapplicability for online analysis (Xu et al., 2016). Nonetheless, its application to the clinical setting is valuable due to its capabilities in biomarker discovery.

### 1.8 Sample pre-concentration strategies

**FIGURE 2:**
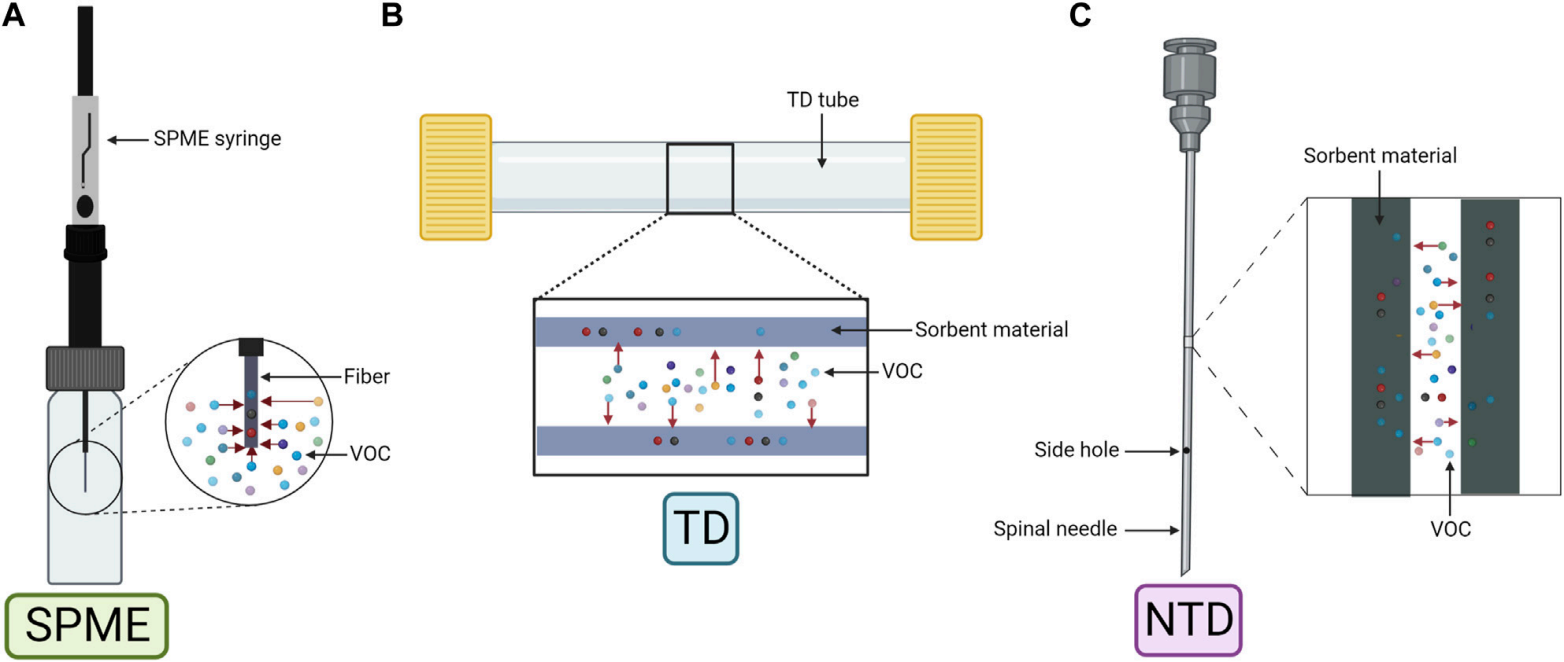
Schematic representation of the three main pre-concentration techniques. **(A)** Solid-phase microextraction (SPME). **(B)** Thermal desorption tube (TD). **(C)** Needle-trap device (NTD). Created with Biorender.com.

Exhaled breath samples, especially mixed breath, require enrichment before offline analysis due to low VOC concentration and high water vapor content. Pre-concentration methods usually include two consecutive steps, consisting of trapping VOCs in sorbents followed by their release via thermal desorption. Three main techniques especially suited for GC are used (Figure 2): solid-phase microextraction (SMPE), thermal desorption tubes (TD), and needle-trap devices (NTDs) (Lawal et al., 2017; Sola-Martínez et al., 2022).

SPME (Figure 2A) was first applied to human breath by Grote and Pawliszyn (1997). Equilibrium is established during sampling based on analyte and sorbent physicochemical properties within the fiber (Beauchamp and Miekisch, 2020). The fiber, coated usually with polydimethylsiloxane (PDMS), Carboxen (Car), or divinylbenzene (DVB), can also have a combination of coatings (Car and/or DVB embedded into PDMS) for a wider chemical species extraction (Trujillo-Rodríguez et al., 2020). Moreover, derivatization reactions can be performed by doping the fiber to increase the affinity of the analyte to the coating (Vas and Vékey, 2004).

TD (Figure 2B) allows longer periods of storage and ease of transport without affecting the sample. The device, composed of a stainless steel or a glass tube, contains sorbent materials like organic polymers (e.g., Tenax TA), graphitized carbon (e.g., Carbopack X) or carbon molecular sieves (e.g., Carboxen). TD can have single- or multi-bed sorbents, with the latter covering a wider range of analytes, but compromising reproducibility due to analyte–sorbent interactions (Lawal et al., 2017; Beauchamp and Miekisch, 2020; Sola-Martínez et al., 2022).

NTD (Figure 2C) is less common but shares similarities with SPME and TD. It uses a needle-shaped device filled with sorbent materials to capture compounds by drawing breath through the needle. Similar to SPME, NTD requires a small sample volume, although the sensitivity is volume dependent as in TD (Trefz et al., 2012). Storage and transportation are also similar to that of TD (Lawal et al., 2017).

### 1.9 Metabolomics

Metabolomics has gained significant attention in clinical research, providing insights into the pathological pathways of various pathologies. These studies can be broadly categorized into two approaches: untargeted and targeted. Untargeted metabolomics is the non-biased approach, which aims to study as many metabolites as possible to discover changes among the groups of samples, while targeted metabolomics focuses on specific metabolites, offering better sensitivity and specificity. Combining both approaches allows for hypothesis generation (untargeted) and the validation of findings (targeted). Workflows and methodologies for both approaches have subtle differences (Patti et al., 2012).

## 2 Objectives and literature search

This review aims to identify potential VOC biomarkers that are consistent across different pathologies and to consolidate and discuss the methodologies employed for exhaled breath sampling and analysis. To achieve this, a literature search was conducted, focusing on studies that analyzed human exhaled breath by GC-MS published since 2012. The search strategy utilized specific keywords, such as “volatile organic compounds,” “exhaled breath” or “breath test,” “gas chromatography,” and “mass spectrometry.” The databases employed were Scopus and Web of Science. Initially, 377 articles were obtained, which were then narrowed down to 152 after title and abstract evaluation, and the articles were sorted according to the pathology studied. Finally, 70 articles focusing on 10 pathologies of significant interest were included in this review, and categorized in: cancer (such as lung, gastric, colorectal, and breast cancers), other pulmonary pathologies (comprising asthma, COPD, OSA, and cystic fibrosis), and infectious pathologies (encompassing community-acquired pneumonia (CAP)/hospital-acquired pneumonia (HAP)/ventilator-associated pneumonia (VAP) and COVID-19).

## 3 VOCs in exhaled breath in health and pathology

**FIGURE 3:**
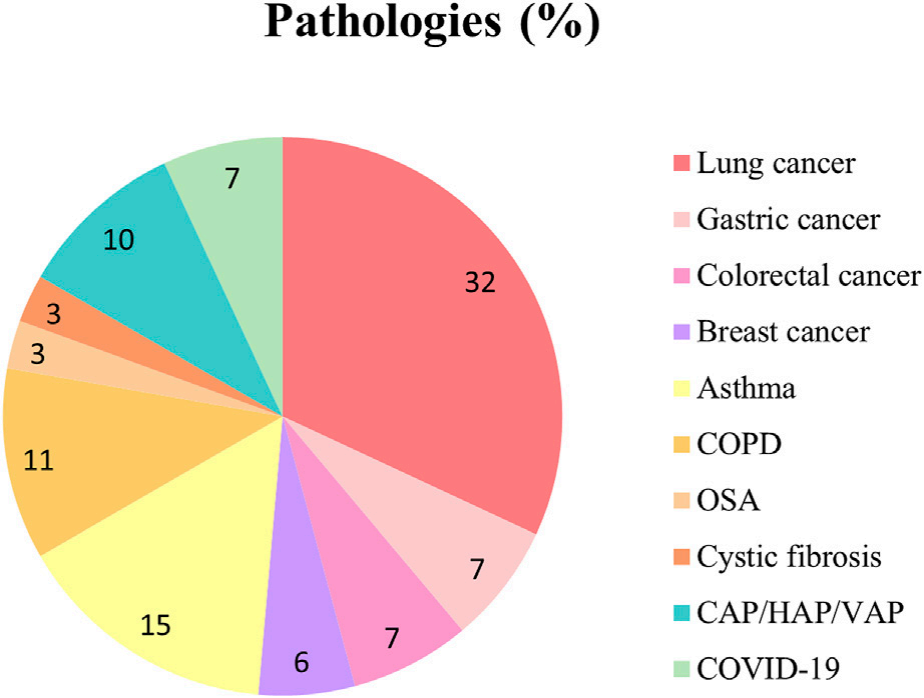
Pie plot depicting the proportion of studies for each pathology included in this review. CAP, community-acquired pneumonia; COPD, chronic obstructive pulmonary disease; HAP, hospital-acquired pneumonia; OSA, obstructive sleep apnea; VAP, ventilator-associated pneumonia.

In the following sections, selected studies for each pathology are discussed, along with the identified candidate VOCs reported as pathology-specific biomarkers. Figure 3 illustrates the distribution of studies, showing that lung cancer has been the most extensively studied, followed by asthma, COPD, and CAP/HAP/VAP.

### 3.1 VOCs in cancer

#### 3.1.1 Lung cancer

Lung cancer (LC) is the second most diagnosed cancer, and the leading cause of cancer-related deaths (Ferlay et al., 2021). LC comprises two major histological types: small-cell lung cancer (SCLC) and non-small-cell lung cancer (NSCLC) (Rodak et al., 2021). The 5-year relative survival rates for localized NSCLC and SCLC are 65% and 30%, dropping to 9% and 3% when metastasized (2012–2018), respectively (Lung Cancer Survival Rates, 2023). Symptoms may be absent, non-specific, or easily confused with other pulmonary pathologies (Balata et al., 2022).

Low-dose computed tomography (LDCT) is the main screening tool, although it exhibits a high false-positive rate (Nooreldeen and Bach, 2021). Lung tissue biopsy, the gold standard procedure for diagnosis, determines malignancy, histological type, and TNM (tumor, nodule, and metastases) stage. However, this procedure is highly invasive and can lead to complications, such as pneumothorax and pneumonia (Zhang et al., 2020b). Indeed, the development of rapid and non-invasive early diagnostic tests is urgently required, and breath tests offer promising alternatives. Among the pathologies studied in exhaled breath, LC is the most prevalent, as this pathology is directly related to the respiratory tract.

Twenty-three studies focusing on potential biomarkers for LC are summarized in Supplementary Tables S1–S3, referring to metabolomic methodology, group comparisons, and VOC biomarkers, respectively. A Chinese group performed two untargeted studies on the same data, comparing LC patients and healthy controls (HCs). The first study (Zou et al., 2021) developed a prediction model based on the whole breath profile (308 peaks), achieving 85.0% accuracy, 83.0% sensitivity, and 85.0% specificity. Twenty-two discriminative VOCs were annotated, styrene being also found downregulated in LC patients who responded partially to treatment or remained stable (Supplementary Table S3), along with two other VOCs (dodecane, 4-methyl and α-phellandrene) (Nardi-Agmon et al., 2016). The second study (Zou et al., 2022) selected 31 VOCs as biomarkers in the univariate analysis (UVA), which showed 0.787 AUC in the multivariate analysis (MVA) after cross-validation. Additionally, eight VOCs were found to be involved in a total of 18 metabolic pathways, of which 11 were significantly altered.

A Polish group compared LC patients with HCs. Buszewski et al. (2012) divided both groups according to smoking habits, identifying 12 significant VOCs between non-smokers, 7 being upregulated when compared to active smokers. Rudnicka et al. (2014) measured 43 VOCs and developed a model with 88 features, yielding 0.970 AUC, 74.0% sensitivity, and 73.0% specificity, with dimethyl sulfide as the main discriminating VOC. Ligor et al. (2015) applied machine learning algorithms, and the final model formed by eight compounds showed an value (e.i. 0.650) AUC. In a subsequent study (Rudnicka et al., 2019), the model containing seven VOCs selected from the UVA presented an improved performance, showing 86.4% sensitivity and specificity in the test group. Twelve VOCs were found in common between these four abovementioned studies (Supplementary Table S3).

Schallschmidt et al. (2016) focused on 24 VOCs previously selected as potential LC biomarkers, 20 being also reported in other studies (Supplementary Table S3). In the UVA, 11 and 7 VOCs were significantly altered between LC patients and HCs (non-smokers and active smokers, respectively), 8 VOCs seemingly unrelated to smoking. Moreover, four models were constructed with different subsets of the targeted VOCs, achieving the highest sensitivity (92.0%) with a subset of four VOCs, and the highest specificity (96.0%) with seven VOCs. Ethanol and octane were two target VOCs proposed as potential biomarkers in other studies (Supplementary Table S3).

Sakumura et al. (2017) reported ethanol, along with other four VOCs, in a study classifying LC and HCs using a support vector machine (SVM) algorithm, achieving 89.0% accuracy, a 94.4% true-positive ratio, and a 89.7% true-negative ratio when combining different subsets of five VOCs. Furthermore, the distance to the SVM classification boundary provided information on the cancer stage, with early-stage LC located closer to the boundary than advanced-stage LC.

Two research groups from China and Greece conducted several studies comparing LC patients, pulmonary non-malignant disease (PNMD) patients, and HCs. The Chinese group conducted 4 studies, sharing 27 VOCs (Supplementary Table S3). Wang et al. (2012) found 23 significant VOCs with AUCs >0.6, unrelated to smoking, as potential biomarkers, of which five VOCs were significant between squamous carcinoma and adenocarcinoma LC patients. The discrimination model for LC, PNMD, and HCs could correctly classify 96.5% of LCs. Zou et al. (2014) selected five VOCs as LC-specific biomarkers, achieving AUCs ranging from 0.672 to 1 in a validation cohort, with hexadecanal being the most discriminative. Additionally, Chen et al. (2021) annotated 19 VOCs that could discriminate LC from PNMD, as well as 20 VOCs that differentiated LC from HCs with AUCs of 0.809 and 0.987, respectively. Moreover, LC patients could be distinguished by histology (NSCLC and SCLC) using 20 VOCs value (e.i. 0.939) AUC and stage (early and advanced) with 19 VOCs value (e.i. 0.827) AUC. The Greek group used both targeted and untargeted approaches on the same data set. The targeted study (Koureas et al., 2020) included 19 VOCs, of which 17 VOCs were also found in other studies (Supplementary Table S3). In the UVA, seven VOCs showed significance when comparing LC, PNMD, and HCs, although no single VOC was altered between LC and PNMD. However, LC and HCs were correctly classified by either including 19 VOCs, nine VOCs selected in the UVA (LC vs. HCs), or a subset of VOCs identified by feature selection (FS) (AUCs 0.769–0.970). In the untargeted study (Koureas et al., 2021), 29 features were considered for the analysis, 18 features (12 VOCs annotated) showing significance between LC and HCs, and only 2 (1 VOC annotated) among LC and PNMD. Moreover, LC and HCs were correctly classified using either 29 features or a subset of eight features identified by FS (AUCs 0.940 and 0.960, respectively). In the case of LC and PNMD, three VOCs achieved 75.0% discrimination accuracy value (e.i. 0.820) AUC. Among the features/VOCs from both approaches, three VOCs (one from the targeted and two from the untargeted) achieved an accuracy of 72.0% in discriminating LC and PNMD value (e.i. 0.780) AUC. However, the VOC from the targeted study was detected in extremely low frequencies. Another targeted study focusing on 21 VOCs identified four upregulated VOCs in LC compared to PNMD (Corradi et al., 2015), of which two (hexane and ethylbenzene) were also included in the targeted study by Koureas et al. (2020) (Supplementary Table S3), showing elevated increased levels in adenocarcinoma LC (hexane) and in advanced-stage LC (ethylbenzene) patients.

Furthermore, both untargeted and targeted approaches were performed in the same study comparing LC, COPD, asthmatic patients, and HCs. Monedeiro et al. (2021) built an RF model with the 12 most important VOCs from the untargeted analysis, achieving an overall accuracy of 85.7%. In the following targeted approach, 29 VOCs were preselected, of which 9 were used to build the classification model that provided 91.0% overall accuracy. Additionally, Callol-Sanchez et al. (2017) identified nonanoic acid significantly altered in LC patients compared to both COPD patients and HCs in a targeted study, and Muñoz-Lucas et al. (2020) found elevated levels of propionic acid in LC patients with COPD, mainly detected in advanced-stage LC.

#### 3.1.2 Gastric cancer

Gastric cancer (GaC) is among the five deadliest cancers in 2020, according to the World Health Organization (WHO) (Cancer, 2023). The 5-year relative survival rate is 72% when localized and decreases to 6% when distant at the time of diagnosis (2012–2018) (American Cancer Society, 2017). The main risk factors for GaC, which is predominantly sporadic (90%), include smoking, high meat intake, alcohol consumption, obesity, and *Helicobacter pylori* infection (Conti et al., 2023). Persistent *H. pylori* infection causes chronic inflammation, leading to precursor lesions associated with GaC: atrophy, metaplasia, dysplasia, and carcinoma (Conti et al., 2023).

The gold standard diagnostic technique is upper endoscopy, followed by a biopsy, although it is invasive and requires specialists (Hamashima, 2016). While high-incidence countries have implemented screening programs, low-incidence countries require cost-effective alternatives (Herrera-Pariente et al., 2021). Serum biomarkers, which include carcinoembryonic antigen (CEA), alpha-fetoprotein (AFP), and carbohydrate antigens (CA19-9 or CA72-4), have been used for early diagnosis, but their lack of specificity results in low positive rates and the inability to detect precancerous lesions (Feng et al., 2017).

Five studies focusing on biomarkers for GaC, all employing an untargeted metabolomics approach, are included in Supplementary Tables S1–S3. Two studies, conducted in China and Latvia, compared GaC patients, peptic ulcer disease (PUD) patients, and controls. Xu et al. (2013) identified three upregulated VOCs in GaC and four VOCs upregulated in PUD compared to HCs, with one VOC (furfural) shared among comparisons. Likewise, Amal et al. (2013) found four VOCs upregulated in GaC of which two were also upregulated in PUD. However, no single discriminating VOC between GaC and PUD was identified in any of the studies. Only one VOC was found to be common to different geographical areas, 6-methyl-5-hepten-2-one (Supplementary Table S3).

Tong et al. (2017) reported 11 candidate GaC biomarkers comparing GaC patients with PUD, gastritis patients, and HCs, using UVA and MVA. One VOC, nonanal, was also found by Amal et al. (2013) to be significantly altered between GaC, PUD, and an additional group stratified based on the operative link on gastric intestinal metaplasia (OLGIM), which classifies patients according to the presence/absence and stage of precancerous lesions. Among the multiple comparisons, eight VOCs showed alterations among groups, seven of which were upregulated in GaC compared to OLGIM, only one VOC being altered between GaC and OLGIM III-IV, and three VOCs in PUD compared to OLGIM.

Lastly, Bhandari et al. (2023) explored the correlation between the fecal microbiome and exhaled breath VOCs. Two VOCs (1-octanol and dioctyl ether) were significantly altered and exclusively present in GaC. Moreover, 14 VOCs from GaC patients were correlated with 33 fecal bacterial taxa, and 7 VOCs from HCs were correlated with 17 bacterial taxa, with no common VOCs between groups.

#### 3.1.3 Colorectal cancer

Colorectal cancer (CRC) ranks among the most common cancers worldwide (Ferlay et al., 2021). The 5-year relative survival drops from 65% to 15.6% when diagnosed at later stages (2013–2019), which represents 23% of cases, as early symptoms are not pathology-specific (Colorectal Cancer—Cancer Stat Facts, 2023). The most applied screening tools are the fecal immunochemical test (FIT) and colonoscopy (Helsingen and Kalager, 2022). The FIT test is based on the measurement of the amount of hemoglobin in feces, and one-third of stage I cancers are missed (Niedermaier et al., 2020). Colonoscopy, while effective, is invasive, time-consuming, and expensive and is performed with conscious sedation, carrying the risk of colonic perforation and major bleeding (Qaseem et al., 2019; Helsingen and Kalager, 2022). Other CRC screening tests such as the guaiac-based fecal occult blood test (gFOBT), sigmoidoscopy, fecal biomarker panel test, and computed tomography (CT) colonography have several limitations, such as false-positive results, invasiveness, and high cost (Qaseem et al., 2019).

Five studies focusing on potential biomarkers for CRC are summarized in Supplementary Tables S1–S3, all employing an untargeted metabolomics approach. The studies that analyzed mixed breath sampled the same cohort of CRC patients. The first study (Altomare et al., 2013) compared CRC and HCs by selecting a pattern of 15 VOCs by UVA to construct the probabilistic neural network (PNN) model, which yielded 76.0% accuracy in the validation cohort. In a subsequent study (Altomare et al., 2015), the data were reprocessed, and 32 of 52 CRC patients were resampled after cancer removal. The PNN model was constructed with 31 VOCs selected by UVA, yielding 97.5% and 97.7% accuracies discriminating pre- and post-surgery CRC patients, and post-surgery CRC and HCs, respectively. Additionally, 11 VOCs shared with the previous study could discriminate pre- and post-surgery CRC patients with 98.8% accuracy. These results demonstrate the metabolic change in exhaled VOC patterns due to cancer cell metabolism and suggest that metabolism does not return to the pre-cancer state after cancer removal.

Another study by the aforementioned research group investigated potential biomarkers of cancer stages (early/I–II or advanced/III–IV) (Altomare et al., 2020). Fifteen VOCs were selected by UVA comparing CRC and HCs, to build a model that included age class (>65 vs. ≤65 year olds). Fourteen identified VOCs could discriminate CRC from HCs, with a 93.0% overall positive predictive value (PPV) after cross-validation, whereas eight and five VOCs could discriminate early-CRC from HCs with an 86.0% PPV and advanced-CRC from HCs with a 91.0% PPV. Three common VOCs between UVA and MVA, namely, ethylbenzene, methylbenzene, and tetradecane, were quantified to establish the threshold concentration values. However, none of these compounds were reported in other studies. Nevertheless, five out of the 15 VOCs were common with previous studies (Altomare et al., 2013, 2015), and three were reported as significantly altered between CRC and HCs: 4-methyloctane and ethanol (research group from Latvia) (Amal et al., 2016) and dodecane (research group from China) (Wang et al., 2014a) (Supplementary Table S3).

Likewise, Amal et al. (2016) found four significantly altered VOCs between CRC and HCs, which were identified by UVA and subsequently quantified: 4-methyloctane and ethanol were downregulated, whereas acetone and ethyl acetate were upregulated. Likewise, Wang et al. (2014a) found nine potential biomarkers (eight upregulated and one downregulated) for CRC patients with adenocarcinoma by MVA.

#### 3.1.4 Breast cancer

Breast cancer (BC) is the most diagnosed type of cancer and the fifth cause of cancer-related mortality (Cancer; Ferlay et al., 2021). While the 5-year relative survival rate stands at 90.8%, it drops dramatically to 31% when diagnosed at a distant stage (2012–2019) (Female Breast Cancer, 2023). The current gold standard screening methods include annual mammography and clinical breast examination for women over the age of 40. Unfortunately, physical breast examinations, even when performed by a physician, fail to reduce mortality (Barba et al., 2021). Regarding mammography, the sensitivity is compromised by breast density (Boyd et al., 2007), and the procedure requires X-ray examination and may lead to overdiagnosis, resulting in unnecessary procedures and treatments (Løberg et al., 2015). Alternative screening approaches, such as digital breast tomosynthesis (DBT), ultrasonography, magnetic resonance imaging (MRI), and positron emission tomography/computed tomography (PET/CT), are hampered by high costs, discomfort, the requirement for trained technicians, and radiation exposure (Barba et al., 2021). Therefore, there is an urgent requirement for innovative screening tools that can overcome these drawbacks, and breath tests show promise as a potential approach.

Four studies focusing on potential BC biomarkers are summarized in Supplementary Tables S1–S3. These studies were conducted in the same geographical area (China). The targeted study by Li et al. (2014) focused on four aldehydes and their potential to discriminate between BC patients, breast non-malignant disease (BNMD) patients, and HCs. All the targeted aldehydes were significantly upregulated in BC, while hexanal was upregulated in BNMD, both compared to HC. Furthermore, nonanal was increased in BC when compared to BNMD. The combination of these VOCs showed 91.7% sensitivity and 95.8% specificity (0.934 AUC) in discriminating early-stage BC from HCs, and the predictive model achieved 80.4% correct classification after leave-one-out cross-validation (LOOCV). Hexanal was also identified as a potential biomarker in a different study (Supplementary Table S3).

Two untargeted studies compared BC with HCs and BNMD. Barash et al. (2015) identified 23 VOCs by UVA, 21 of which showed significant differences between HCs and patients with breast lesions [BC, BNMD, and an additional group of patients with ductal carcinoma *in situ* (DCIS)], and four VOCs were significant between BC and DCIS. The MVA revealed 14 VOCs that could discriminate BC from HC and BNMD, and from DCIS, yielding 72.0% and 81.0% accuracies after LOOCV, respectively. Additionally, two of these 14 VOCs were consistent with findings from other studies (Supplementary Table S3). Wang et al. (2014b) annotated 28 potential biomarkers, of which 21, 6, and 8 VOCs were significantly altered in BC when compared separately to HCs, BNMD (cyclomastopathy and mammary gland fibroma), and DCIS, respectively. Among these, three VOCs, namely, cyclohexanone, 1,4-dimethoxy-2,3-butanediol, and 2,5,6-trimethyloctane, were upregulated in BC compared to both HCs and BNMD. Only cyclohexanone was again reported by Zhang et al. (2020a) (Supplementary Table S3).

Furthermore, Zhang et al. (2020a) subdivided the BC group into DCIS, lymph node metastasis-negative (LNMN), and lymph node metastasis-positive (LNMP), annotating 13, 12, and 17 significant VOCs when compared to HC, respectively. An additional group of GaC patients was included for comparison with BC, yielding 17 significant VOCs. The set of seven overlapping VOCs among all comparisons could discriminate BC and the different subgroups from HCs value (e.i. 0.864–0.943) AUC, sensitivity 80.8%–96.2%, and specificity 71.6%–100%.

### 3.2 VOCs in other pulmonary pathologies

#### 3.2.1 Asthma

Asthma is a chronic and heterogeneous lung pathology characterized by inflammation and airway obstruction, manifesting with variable symptoms that include cough, wheezing, shortness of breath, and chest tightness (Asthma, 2023; Asthma-Diagnosis, 2023). This pathology places a significant economic burden on healthcare systems, affecting approximately 292 million people worldwide, typically being developed during childhood. Asthma’s impact on patients’ quality of life and the risk of premature death are major concerns (The Global Asthma Report, 2022).

Diagnosis relies on spirometry, bronchoprovocation tests, peak expiratory flow tests, allergy skin or blood tests, and FeNO tests (Asthma-Diagnosis, 2023). Patients may experience a loss of pathology control and acute exacerbations of symptoms, leading to significant morbidity and a progressive loss of lung function (Castillo et al., 2017). Moreover, the heterogeneity of asthma, concerning severity and response to treatment, is a consequence of the underlying pathophysiological mechanisms. Patients can be classified into different phenotypes based on observable characteristics (steroid response, obesity, allergies, etc.), or endotypes based on the underlying cellular and molecular mechanisms (Kuruvilla et al., 2019). In this context, breath tests offer a non-invasive and easy-to-perform approach for early diagnosis and exacerbation prediction, especially suitable for children, and could be also used to define phenotypes and endotypes by analyzing the profile of endogenous VOCs, which reflects the inflammatory state of the bronchia and underlying molecular mechanisms involved, allowing a significant improvement in treatment effectiveness.

Eleven untargeted studies focused on asthma are included in Tables 1, 2; Supplementary Table S4. Several studies were conducted by the same research group focusing on asthmatic children. Van Vliet et al. (2016, 2017) studied the loss of asthma control and exacerbation episodes over a period of 1 year. In the first study (Van Vliet et al., 2016), a combination of 15 VOCs (10 annotated) showed 86.0% accuracy in classifying persistently controlled and uncontrolled asthma, although no association was found between different exhaled inflammatory markers [FeNO, exhaled breath condensate (EBC), and VOCs] and asthma control. Subsequently (Van Vliet et al., 2017), in a larger cohort of asthmatic children, the combination of seven VOCs used to construct the RF model could predict 88.0% of asthma exacerbation episodes within 14 days. These two studies shared only two VOCs: 1,2-dimethylcyclohexane and 2-methylfuran (Table 2). Additionally, Robroeks et al. (2013) annotated 30 VOCs related to asthma exacerbation, and the models combining six and seven VOCs could correctly classify 96.0% of baseline and exacerbation samples taken from the same patient (100% sensitivity and 93.0% specificity) and 91.0% of patients who would have future exacerbations or not, respectively. These results suggest that the profile of VOCs can identify exacerbations and could be used to predict which patients will suffer these episodes. Additionally, Smolinska et al. (2014) studied a cohort of wheezing children with HCs between the ages of 2 and 4 years until the age of 6 years, to find potential biomarkers for preclinical asthma. A total of 17 VOCs (13 annotated) were selected by comparing asthmatic children with HCs and with transient wheezers, which could correctly classify 80.0% of the wheezing children at inclusion, differentiating those who would develop asthma from those who were transient wheezers. Notably, three VOCs reported in these studies (2-methylfuran, 3-methylfuran, and m-cymene) were also identified by Monedeiro et al. (2021) when comparing LC, COPD asthmatic patients, and HCs. In this study, the model built with 12 VOCs from the untargeted data presented 85.7% overall accuracy, and another with 9 of the 29 targeted VOCs provided 91.0% overall accuracy.

**TABLE 1:** Summary of studies focused on asthma, chronic obstructive pulmonary disease, obstructive sleep apnea, and cystic fibrosis. AB, alveolar breath; ABS, adaptive breath sampler; CF, cystic fibrosis; COPD, chronic obstructive pulmonary disease; GC, gas chromatography; GC×GC, two-dimensional gas chromatography; LC, lung cancer; MB, mixed breath; MS, mass spectrometry; na, not applicable; nd, not detailed; NIST, National Institute of Standards and Technology; NTD, needle-trap device; OSA, obstructive sleep apnea; SPME, solid-phase microextraction; TD, thermal desorption tube; TOF, time-of-flight; UI, ultra-inert, VOCs, volatile organic compounds.

Reference	Pathology	Methodology	Sample	Sampling	Analysis technique	Sorbent material	Column	IS	Identification	
									Library	Authentic STD
Gahleitner et al. (2013)	Asthma	Untargeted	AB	ABS	TD-GC-MS	Tenax/Carbotrap	nd	No	NIST	Yes
Sola-Martínez et al. (2021)	Asthma	Untargeted	MB	Tedlar^®^ bag	TD-GC-MS	Tenax TA	HP-5MS UI (30 m × 0.25 mm × 0.25 μm) (Agilent)	No	NIST	No
Schleich et al. (2019)	Asthma	Untargeted	MB	Tedlar^®^ bag	TD-GC-TOF-MS/TD-GCxGC-TOF-MS	Carbograph 1TD/Carbopack X and Tenax TA/Carbopack B	RTX-5MS (30 m × 0.25 mm × 1 μm) (Restek) and Rxi-624Sil MS (30 m × 0.25 μm × 1.4 μm) (Restek) 1D and Stabilwax (2 m × 0.25 μm × 0.5 μm) (Restek) 2D	No	NIST	Yes
Brinkman et al. (2017)	Asthma	Untargeted	MB	Tedlar^®^ bag	TD-GC-MS	Tenax GR	VF1-MS column (30 m × 0.25 mm × 1 μm) (Varian)	No	NIST	No
Van Vliet et al. (2017)	Asthma	Untargeted	MB	Tedlar^®^ bag	TD-GC-TOF-MS	Carbograph 1TD/Carbopack X	nd	No	NIST	No
Van Vliet et al. (2016)	Asthma	Untargeted	MB	Tedlar^®^ bag	TD-GC-TOF-MS	Carbograph 1TD/Carbopack X	nd	No	NIST	No
Meyer et al. (2014)	Asthma	Untargeted	MB	Tedlar^®^ bag	TD-GC-TOF-MS	Carbograph 1TD/Carbopack X	RTX-5MS (30 m × 0.25 mm × 1 μm) (Restek)	No	nd	No
Smolinska et al. (2014)	Asthma	Untargeted	MB	Tedlar^®^ bag	TD-GC-TOF-MS	Carbograph 1TD/Carbopack X	RTX-5MS (30 m × 0.25 mm × 1 μm) (Restek)	No	NIST	No
Robroeks et al. (2013)	Asthma	Untargeted	MB	Tedlar^®^ bag	TD-GC-TOF-MS	Active carbon	RTX-5MS (30 m × 0.25 mm × 1 μm) (Restek)	No	NIST	No
Caldeira et al. (2012)	Asthma	Untargeted	MB	Tedlar^®^ bag	SPME-GC×GC-TOF-MS	DVB/Car/PDMS	HP-5 (30 m × 0.32 mm × 0.25 μm) (Agilent) 1D and DB-FFAP (0.79 m × 0.25 mm × 0.25 μm) (Agilent) 2D	No	In-house library and Wiley and NIST	No
Monedeiro et al. (2021)	LC/COPD/Asthma	Untargeted/targeted	MB	Tedlar^®^ bag	NTD-GC-MS	PDMS/Carbopack/Carboxen	DB-624 capillary column (60 m × 0.32 mm × 1.8 μm) (Agilent)	No	NIST	Yes
Pizzini et al. (2018)	COPD	Untargeted	AB	Glass syringe	TD-GC-TOF-MS	Carbotrap B 80 mg/Carbopack X 260 mg	Restek-Q-Bond (30 m × 0.25 mm × 8 μm) (Restek)	No	NIST	Yes
Basanta et al. (2012)	COPD	Untargeted	AB	—	TD-GC-TOF-MS	Tenax TA/Carbotrap	DB5-MS column (30 m × 0.25 mm x 0.25 μm) (Agilent)	D5-Bromobenzene	NIST	No
Phillips et al. (2012)	COPD	Untargeted	AB	Bio-VOC^®^	TD-GC-MS	Carbograph 1TD/Carbopack X	HP-5MS (30 m × 0.25 mm × 0.25 μm) (Agilent)	No	NIST	No
van Velzen et al. (2019)	COPD	Untargeted	MB	Tedlar^®^ bag	TD-GC-TOF-MS	Tenax GR	VF1-MS column (30 m × 0.25 mm × 1 μm) (Varian)	No	NIST	No
Gaida et al. (2016)	COPD	Untargeted	MB	Stainless steel tube	TD-GC-MS	Tenax TA	nd	No	NIST	Yes
Cazzola et al. (2015)	COPD	Untargeted	MB	Tedlar^®^ bag	SPME-GC-MS	DVB/Car/PDMS 50/30 μg	Equity-5 capillary column (30 m × 0.25 mm × 0.25 μm) (Supelco)	No	NIST	No
Jareño-Esteban et al. (2017)	COPD	Targeted	AB	Bio-VOC^®^	TD-GC-MS	Tenax TA/graphitized carbon black/carbonized molecular sieve	DB-1 (30 m × 0.25 mm × 1 μm) (Agilent)	Hexamethylcyclotrisiloxane	na	Yes
Bayrakli et al. (2016)	OSA	Targeted	AB	Bio-VOC^®^	TD-GC-MS	Tenax TA 200 mg	DB-5 (30 m × 0.25 mm) (Agilent)	No	na	Yes
Aoki et al. (2017)	OSA	Targeted	MB	DuPont™ Tedlar^®^ bag	TD/NTD-GC-MS	nd	nd	No	na	Yes
Woollam et al. (2022b)	CF	Untargeted	MB	Tedlar^®^ bag	SPME-GC-MS	DVB/Car/PDMS	HP-5MS (30 m × 0.25 mm × 0.25 μm) (Agilent)	No	nd	No
van Horck et al. (2021)	CF	Untargeted	MB	Tedlar^®^ bag	TD-GC-TOF-MS	Carbograph 1TD/Carbopack X	nd	No	NIST	No

**TABLE 2:** VOCs reported in asthma and chronic obstructive pulmonary disease (≥2 studies). *EO*, eosinophilic asthma; *na*, not applicable; *NEO*, neutrophilic asthma; *ppbv*, parts per billion by volume; *LOD, Limit of detection.

Asthma									
No	Compound name	CAS-N	Formula	Chemical class	Sign of alteration	Concentration (patients)	Concentration (controls)	Unit	Reference
1	1-Propanol	71-23-8	C_3_H_8_O	Alcohol	Upregulated	9.94	14.59	ppbv	Monedeiro et al. (2021)
					Downregulated (EO)/Upregulated (NEU)	na	na	na	Schleich et al. (2019)
2	Phenol	108-95-2	C_6_H_6_O	Alcohol	Downregulated	na	na	na	Meyer et al. (2014)
					Upregulated	<1.43*	<1.43*	ppbv	Monedeiro et al. (2021)
3	Nonanal	124-19-6	C_9_H_18_O	Aldehyde	Altered	na	na	na	Van Vliet et al. (2017)
					Altered	na	na	na	Caldeira et al. (2012)
					Upregulated	na	na	na	Schleich et al. (2019)
4	Octanal	124-13-0	C_8_H_16_O	Aldehyde	Downregulated	na	na	na	Meyer et al. (2014)
					Altered	na	na	na	Van Vliet et al. (2017)
5	Benzene	71-43-2	C_6_H_6_	Aromatic hydrocarbon	Upregulated	na	na	na	Meyer et al. (2014)
					Altered	na	na	na	Robroeks et al. (2013)
6	m-Cymene	535-77-3	C_10_H_14_	Aromatic hydrocarbon	Upregulated	na	na	na	Gahleitner et al. (2013)
					Altered	na	na	na	Van Vliet et al. (2016)
					Upregulated	0.32	0.61	ppbv	Monedeiro et al. (2021)
7	2,4-Dimethylheptane	2213-23-2	C_9_H_20_	Branched hydrocarbon	Downregulated	na	na	na	Meyer et al. (2014)
					Upregulated	na	na	na	Smolinska et al. (2014)
8	2-Methylpentane	107-83-5	C_6_H_14_	Branched hydrocarbon	Upregulated	4.59	1.24	ppbv	Monedeiro et al. (2021)
					Upregulated	na	na	na	Smolinska et al. (2014)
9	3-Methylpentane	96-14-0	C_6_H_14_	Branched hydrocarbon	Upregulated	1.07	0.24	ppbv	Monedeiro et al. (2021)
					Altered	na	na	na	Robroeks et al. (2013)
10	1,2-Dimethylcyclohexane	583-57-3	C_8_H_16_	Cyclic hydrocarbon	Altered	na	na	na	Van Vliet et al. (2016)
					Altered	na	na	na	Van Vliet et al. (2017)
11	2-Methylfuran	534-22-5	C_5_H_6_O	Ether	Altered	na	na	na	Van Vliet et al. (2016)
					Altered	na	na	na	Van Vliet et al. (2017)
12	Decane	124-18-5	C_10_H_22_	Hydrocarbon (saturated)	Altered	na	na	na	Caldeira et al. (2012)
					Altered	na	na	na	Sola-Martínez et al. (2021)
13	Dodecane	112-40-3	C_12_H_26_	Hydrocarbon (saturated)	Downregulated	na	na	na	Meyer et al. (2014)
					Upregulated	6.27	5.18	ppbv	Monedeiro et al. (2021)
					Altered	na	na	na	Caldeira et al. (2012)
14	Tetradecane	629-59-4	C_14_H_30_	Hydrocarbon (saturated)	Altered	na	na	na	Monedeiro et al. (2021)
					Altered	na	na	na	Caldeira et al. (2012)
15	Undecane	1120-21-4	C_11_H_24_	Hydrocarbon (saturated)	Upregulated	1.78	0.80	ppbv	Monedeiro et al. (2021)
					Downregulated	na	na	na	Schleich et al. (2019)
16	Acetone	67-64-1	C_3_H_6_O	Ketone	Altered	na	na	na	Sola-Martínez et al. (2021)
					Downregulated	na	na	na	Smolinska et al. (2014)
17	Acetonitrile	75-05-8	C_2_H_3_N	Nitrogen-containing	Altered	na	na	na	Brinkman et al. (2017)
					Altered	na	na	na	Monedeiro et al. (2021)

Chronic obstructive pulmonary disease									
No	Compound name	CAS-N	Formula	Chemical class	Sign of alteration	Concentration (patients)	Concentration (controls)	Unit	Reference
1	Isopropanol	67-63-0	C_3_H_8_O	Alcohol	Downregulated	na	na	na	Cazzola et al. (2015)
					Upregulated	258.37	10.55	ppbv	Monedeiro et al. (2021)
2	Phenol	108-95-2	C_6_H_6_O	Alcohol	Altered	na	na	na	Gaida et al. (2016)
					Altered	na	na	na	Phillips et al. (2012)
3	Decanal	112-31-2	C_10_H_20_O	Aldehyde	Altered	na	na	na	Basanta et al. (2012)
					Altered	na	na	na	Phillips et al. (2012)
4	Hexanal	66-25-1	C_6_H_12_O	Aldehyde	Upregulated	na	na	na	Jareño-Esteban et al. (2017)
					Altered	na	na	na	Basanta et al. (2012)
					Altered	na	na	na	Phillips et al. (2012)
5	Nonanal	124-19-6	C_9_H_18_O	Aldehyde	Upregulated	na	na	na	Jareño-Esteban et al. (2017)
					Altered	na	na	na	Basanta et al. (2012)
6	Benzene	71-43-2	C_6_H_6_	Aromatic hydrocarbon	Upregulated	na	na	na	Gaida et al. (2016)
					Altered	na	na	na	Phillips et al. (2012)
7	Toluene	108-88-3	C_7_H_8_	Aromatic hydrocarbon	Upregulated	na	na	na	Gaida et al. (2016)
					Altered	na	na	na	Phillips et al. (2012)
					Altered	na	na	na	van Velzen et al. (2019)
8	Limonene	138-86-3	C_10_H_16_	Cyclic hydrocarbon	Downregulated	na	na	na	Cazzola et al. (2015)
					Altered	na	na	na	van Velzen et al. (2019)
					Altered	na	na	na	Phillips et al. (2012)
					Upregulated	1.71	1.57	ppbv	Monedeiro et al. (2021)
9	Butane	106-97-8	C_4_H_10_	Hydrocarbon (saturated)	Altered	na	na	na	Phillips et al. (2012)
					Downregulated	na	na	na	Pizzini et al. (2018)
10	Tridecane	629-50-5	C_13_H_28_	Hydrocarbon (saturated)	Altered	ns	ns	ns	Gaida et al. (2016)
					Upregulated	28.36	3.43	ppbv	Monedeiro et al. (2021)
11	Acetic acid	64-19-7	C_2_H_4_O_2_	Organic acid	Altered	na	na	na	Gaida et al. (2016)
					Altered	na	na	na	Phillips et al. (2012)

Likewise, two other studies included a cohort of asthmatic children, in this case, compared to HC. Gahleitner et al. (2013) identified a panel of eight candidate VOCs, all of which were upregulated in asthmatic children. Moreover, Caldeira et al. (2012) built a model with the full data set of metabolites (134), yielding a classification rate of 98.0% (96.0% sensitivity and 95.0% specificity). Among these metabolites, six alkanes were related to allergic asthma and four aldehydes and one alkene to HC. The new model that included nine alkanes and aldehydes showed a classification rate of 96.0% (98.0% sensitivity and 93.0% specificity). One VOC from the latter study, decane, was also reported by Sola-Martínez et al. (2021). In this study, a population of women 3 months postpartum was recruited and divided into asthmatics with other coexisting atopic diseases (A-AD) and non-asthmatics, and the latter were further divided into those with and without other atopic diseases (NA-AD and NA-NAD, respectively). Several models were built to compare the different groups, selecting a total of nine VOCs, which could discriminate between asthmatic and non-asthmatic patients, even in the validation cohort (AUCs 0.670–0.900, 71.0%–100% sensitivity, and 60.0%–70.0% specificity), although the accuracy decreased when asthmatic patients were compared to the non-asthmatic groups separately (AUCs 0.680–0.810 for NA-AD and 0.603–0.750 for NA-NAD).

Furthermore, two articles studied asthma phenotypes and endotypes. Schleich et al. (2019) conducted a study on a group of asthmatic patients classified by inflammatory subtypes. From all binary comparisons, 12 VOCs were selected, of which eight were identified as candidate biomarkers. Among them, two VOCs (hexane and 2-hexanone), along with 1-propanol, were selected from the comparison between eosinophilic and paucigranulocytic value (e.i. 0.680) AUC. Meanwhile, the comparison between neutrophilic and paucigranulocytic yielded two VOCs (3-tetradecene and pentadecene) in the discovery phase and another two (undecane and nonanal) in the replication value (e.i. 0.850 and 0.700, respectively) AUC. Furthermore, when comparing neutrophilic to eosinophilic, three VOCs (3,7-dimethylnonane, 1-propanol, and nonanal) were identified in the discovery phase value (e.i. 0.920) AUC, although only nonanal, along with hexane, showed the best classification performance in the replication phase value (e.i. 0.710) AUC. As a result, two (hexane and 2-hexanone) and three (nonanal, 1-propanol, and hexane) VOCs could discriminate eosinophilic and neutrophilic asthma from other phenotypes value (e.i. 0.720 and 0.730, respectively) AUC. Moreover, Meyer et al. (2014), besides building a model based on 16 VOCs that could discriminate asthmatic patients from HC (100% sensitivity and 91.1% specificity), performed a cluster analysis that included clinical, medication features, and four VOCs that were only present in asthmatic patients, to identify different asthma endotypes. As a result, seven clusters were formed, two with non-allergic asthma and five with allergic asthma. Some clusters presented high clinical similarity but different profiles of VOCs, as well as similar profiles and different clinical symptoms. Although no common VOCs were found between these two studies, eight VOCs were shared with others (Table 2).

#### 3.2.2 COPD

COPD is characterized by chronic respiratory symptoms, such as dyspnea, cough, production of sputum, and/or exacerbations, caused by abnormalities in the airways (bronchitis and bronchiolitis) and/or the alveoli (emphysema), resulting in persistent and progressive airflow obstruction. The causes of the pathology are environmental exposures (tobacco smoking, toxic particles, and gases) and/or genetic risk factors. According to the WHO, 3.23 million people died from COPD in 2019, with 90% of deaths (under the age of 70) occurring in low- and middle-income countries. COPD often coexists with chronic pathologies, such as lung infections and cancer, heart problems, depression, and anxiety (GOLDCOPD, 2023).

COPD diagnosis relies on spirometry, with weak specificity. Additional tests, lung imaging and arterial blood gas tests, can help assess pathology severity. The symptoms develop slowly, and even though COPD is not curable, different treatments can be applied. However, under- or misdiagnosis can lead to lack/incorrect treatment (GOLDCOPD, 2023), and most patients are diagnosed when the lung damage is irreversible (Fazleen and Wilkinson, 2020). Detecting early or pre-COPD cases, where clinical signs are absent or airflow obstruction is not evident in spirometry, can be challenging. Breath tests offer a valuable tool for identifying these cases that diagnostic tests may miss.

Eight studies are indicated in Tables 1, 2; Supplementary Table S4. Jareño-Esteban et al. (2017) targeted five VOCs (hexanal, heptanal, nonanal, propanoic acid, and nonanoic acid) as potential biomarkers. Although hexanal and nonanal were upregulated in COPD patients compared to non-smokers (HC), no significant VOCs were found between COPD patients and active smokers (HC). Both these VOCs were reported in previous studies (Table 2).

Four studies compared COPD patients with HCs, two being performed in the same geographical area (UK), in different research groups. Phillips et al. (2012) applied different machine learning methods, which included a step of FS, to compare the whole group of COPD with HCs, active with former smokers within the COPD group, and COPD with HCs (non-smokers). Of the automatically generated VOCs in the three comparisons (12, 13, and 10, respectively), six overlapped. Likewise, two of these six shared VOCs were reported by Gaida et al. (2016), and another six VOCs were reported in different studies (Table 2). Moreover, Basanta et al. (2012) built a classification model containing 11 VOCs after data reduction (UVA and PCA), with an accuracy of 70.0%. The groups were further divided and compared by smoking status, improving the performance of the model, especially when active smokers were compared (91.0% accuracy). Furthermore, four VOCs were correlated with sputum eosinophils ≥1%, one VOC with sputum eosinophils ≥2%, and four VOCs with exacerbation episodes (≥2/year). The prediction models showed an accuracy of 75.0% and 88.0% for sputum eosinophils ≥1% and sputum eosinophils ≥2%, respectively, and 83.0% for exacerbations, after LOOCV. Of these 11 VOCs, 3 aldehydes (decanal, hexanal, and nonanal) were shared with Jareño-Esteban et al. (2017) and Phillips et al. (2012) (Table 2). Additionally, Gaida et al. (2016) studied two cohorts of COPD patients and HCs from different locations, which were split by smoking habits. Overall, 14 VOCs showed potential as COPD biomarkers, with 4 being reported also by Phillips et al. (2012) (Table 2).

The study by Monedeiro et al. (2021) was previously mentioned in the LC/asthma section, with untargeted and targeted analyses to build classification models that distinguish COPD, LC, asthma patients, and HCs, yielding 85.7% and 91% overall accuracy (untargeted and targeted, respectively). Two of these VOCs (isopropyl alcohol and limonene) were shared with Cazzola et al. (2015), and two additional VOCs were common with other studies (Table 2).

The remaining studies focused on COPD exacerbations. Pizzini et al. (2018) applied UVA and *post hoc* analysis between pairwise combinations, resulting in 12 significant VOCs. Additionally, four VOCs were classified as discriminative for acute exacerbation (A) COPD, two VOCs were classified as discriminative for stable (S) COPD, and two VOCs as associated with COPD. The RF model containing these 12 VOCs could classify COPD patients value (e.i. 0.970, 78.0% sensitivity, and 91.0% specificity) AUC. Meanwhile, van Velzen et al. (2019) sampled the same cohort of COPD patients before (baseline), during, and after (recovery) an exacerbation episode. The UVA between the Clinical COPD Questionnaire (CCQ) symptom scores and VOCs resulted in 10 discriminative compounds. The subsequent MVA discriminated between baseline and exacerbation and between exacerbation and recovery with accuracies of 71.0% and 75.0% , respectively.

#### 3.2.3 OSA

OSA is a respiratory disorder with an incidence of 24% in men and 9% in women (30–60 years of age), affecting nearly 1 billion people worldwide (Lv et al., 2023). OSA is characterized by the repeated collapse of the pharynx, leading to episodes of apnea or hypopnea accompanied by decreased oxygen levels and interruptions in sleep. It is associated with poor sleep quality and daytime sleepiness, as well as an increased risk for several metabolic and cardiovascular pathologies (arterial hypertension, diabetes, etc.), and depression (Lévy et al., 2015; Schwarz et al., 2017; Nowak et al., 2021). Current OSA diagnosis relies on sleep examination (monitoring sleep stages and cycles), mainly through polysomnography, a costly, time-consuming, and inconvenient test. Although home tests are available, these devices are subject to more measurement errors compared to polysomnography (Kapur et al., 2017). Moreover, several nights should be monitored to obtain a more reliable diagnosis (Stöberl et al., 2017). Therefore, breath tests are presented as a potential tool for both the screening and diagnosis of OSA.

Two independent targeted studies focusing on OSA are included in Table 1 and Supplementary Table S4. Bayrakli et al. (2016) studied the levels of acetone and butanol in patients before and after sleep. Although butanol was upregulated in patients compared to HC (after sleep), this VOC was not significantly increased between patients (before vs. after sleep). Conversely, Aoki et al. (2017) focused on 14 VOCs, which included aromatic, alicyclic, chain hydrocarbons, isoprene, acetone, and ethanol, and classified OSA patients into moderate, severe, and most severe in terms of the apnea–hypopnea index (AHI). The UVA yielded four VOCs upregulated in all OSA patients, four VOCs in severe and most severe OSA patients, and three VOCs exclusively in the most severe OSA patients compared to HCs. Furthermore, four of these VOCs (ethylbenzene, p-xylene, phenylacetic acid, and nonane) showed increased levels according to OSA severity, being correlated with the AHI, arousal index, and duration of percutaneous oxygen saturation (SpO_2_) ≤ 90%. Additionally, the levels of acetone and isoprene decreased after continuous positive airway pressure treatment. Nevertheless, no common VOCs were found between these two studies.

#### 3.2.4 Cystic fibrosis

Cystic fibrosis (CF) is an autosomal recessive genetic pathology caused by a mutation in the *cystic fibrosis transmembrane conductance regulator* (CFTR) gene. This mutation disrupts the cells’ electrolyte transport system, affecting mainly organs with secretory functions, such as as the lungs, pancreas, and reproductive system (Cystic Fibrosis-Causes, 2023). In the lungs, altered sodium absorption results in thick, hardened secretions, increasing the risk of respiratory infections, inflammation, and oxidative stress (Roesch et al., 2018). Pulmonary exacerbations (PEx) are frequent events in the progression of the pathology, potentially leading to permanent lung function loss, reduced quality of life, and decreased survival. PEx treatment includes antibiotics, but delayed symptom onset worsens outcomes (Goss, 2019). The identification of PEx relies on symptomatology, clinical evaluation, and the measurement of changes in forced expiratory volume in one second (FEV1pp) using spirometry devices (Goss, 2019). The use of breath tests to predict PEx in CF is a promising approach. In two independent untargeted studies, CF PEx in children was studied, as shown in Table 1; Supplementary Table S4. van Horck et al. (2021) performed a 1-year observational pilot study, recruiting patients from three different centers. The RF model with the nine most discriminating VOCs could predict 79.0% of patients with stable or upcoming PEx (within 7 days) (79.0% sensitivity and 78.0% specificity). However, no single VOC was found significantly altered when applying UVA between stable and CF PEx patients. Meanwhile, Woollam et al. (2022b) divided the CF patients into CF baseline (not suffering from PEx) and CF PEx. Four VOCs were found to be correlated with FEV1pp at the time of breath collection, of which two VOCs (4-methyl-octane and 3,7-dimethyldecane) were further correlated with changes in FEV1pp. Moreover, four VOCs were found to be significantly different between CF baseline and CF PEx patients: 3,7-dimethyldecane, durene, and 5-methyltridecane were downregulated, and 2,4,4-trimethyl-1,3-pentanediol 1-isobutyrate was upregulated in PEx patients. Although both studies aimed to identify differential VOCs between CF stable and CF PEx patients, none of the reported were shared.

### 3.3 VOCs in infectious pathologies

#### 3.3.1 Pneumonia (CAP/HAP/VAP)

CAP, HAP, and VAP are lower respiratory tract infections associated with high morbidity, mortality, and healthcare costs (Ferreira-Coimbra et al., 2020; Munro et al., 2021; Alnimr, 2023). HAP is developed after 48 h of hospitalization, while VAP is the most frequent infection in the intensive care unit (ICU), developed after endotracheal intubation (Modi and Kovacs, 2020). The pathogens involved encompass Gram-positive bacteria (*Staphylococcus aureus* and *Streptococcus pneumoniae*), Gram-negative bacteria (*Pseudomonas aeruginosa*, *Haemophilus influenzae*, *Klebsiella pneumoniae*, and *Acinetobacter baumannii*), and fungi (*Aspergillus* spp. and *Candida* spp.) (Filipiak et al., 2013, 2015).

Current diagnostics rely on clinical, radiological, and microbiological cultures of respiratory samples [endotracheal aspirates, bronchoalveolar lavage (BAL), and protected specimen brush], which present high inter-variability and moderate sensitivity and specificity. The microbiological confirmation can take several days, leading to overtreatment with antibiotics until the specific pathogen is identified (Fernando et al., 2020; Modi and Kovacs, 2020). Therefore, there is an urgent requirement for less invasive and faster diagnostic techniques.

In the case of VAP, van Oort et al. (2017a) presented a protocol for a prospective multicenter study named BreathDx (Molecular Analysis of Exhaled Breath as Diagnostic Test for Ventilator-Associated Pneumonia), aiming to develop a breath test capable of distinguishing suspected VAP patients, with a 99% sensitivity for culture-positive cases. It also aimed to identify unique VOC patterns that could predict specific pathogen infections, holding promise for more efficient VAP diagnosis and treatment.

Seven studies focusing on VAP, and one on CAP/HAP, are summarized in Tables 3–5. To date, two studies have been conducted in relation to BreathDx. Van Oort et al. (2022) performed an untargeted study within a group of intubated and ventilated ICU patients with suspected VAP, further divided into culture-positive (CP) and culture-negative (CN) BAL samples. Moreover, two platforms were used to cover a wider range of compounds: GC-MS-1 for more volatile compounds and GC-MS-2 for heavier and cyclic volatile compounds. The discriminative model that included 20 VOCs previously selected by UVA and MVA showed 0.830–0.870 AUCs, even when applied to a different set of samples. Furthermore, Ahmed et al. (2023) performed a targeted study focusing on microbial VOCs (mVOCs) previously selected from bacterial species associated with VAP (*S. aureus*, *P. aeruginosa*, *K. pneumoniae*, and *Escherichia coli*). In the case of CP for *S. aureus*, two VOCs were upregulated compared to the other patients value (e.i. 0.790–0.870) AUC. In the case of CP for *P. aeruginosa*, two VOCs were downregulated compared to CP for other pathogens, and one of these VOCs (identified as 3-methylbutanal) was common with CP for *S. aureus*. Moreover, those VAP patients with CP for bacteria known to metabolize tryptophan (*E. coli*, *Klebsiella oxytoca*, and *H. influenzae*) presented increased levels of indole. Despite the fact that both studies followed the same BreathDx protocol, no shared VOCs were identified. However, two VOCs (dimethyl sulfide and tetrahydrofuran) reported by Van Oort et al. (2022) and another two (3-methylbutanal and acetone) by Ahmed et al. (2023) were also found in other studies (Table 5).

**TABLE 3:** Summary of studies focused on pneumonia and COVID-19. AB, alveolar breath; BSG, breath-gas sampler; CAP, community-acquired pneumonia; GC×GC, two-dimensional gas chromatography; HAP, hospital-acquired pneumonia; MB, mixed breath; MS, mass spectrometry; na, not applicable; nd, not detailed; NIST, National Institute of Standards and Technology; QTOF, quadrupole time-of-flight; SPME, solid-phase microextraction; TD, thermal desorption tube; TOF, time-of-flight; VAP, ventilator-associated pneumonia.

Reference	Pathology	Methodology	Sample	Sampling	Analysis technique	Sorbent material	Column	IS	Identification	
									Library	Authentic STD
Van Oort et al. (2022)	VAP	Untargeted	MB	BGS	TD-GC-MS	Carbograph 5TD 300 mg/Tenax GR 90 mg	VF1-MS column (30 m × 0.25 mm × 1 μm) (Varian)	Acetone-D8, hexane-D14, toluene-D8, and xylene-D10	NIST	No
Van Oort et al. (2017a)	CAP/HAP	Untargeted	MB	—	TD-GC-MS	Tenax GR 250 mg	VF1-MS column (30 m × 0.25 mm × 1 μm) (Varian)	No	NIST	No
Gao et al. (2016)	VAP	Untargeted	MB	—	TD-GC-MS	Tenax TA	Rtx-5MS (30 m × 0.25 mm × 0.25 μm)	No	NIST	No
Fowler et al. (2015)	VAP	Untargeted	MB	—	TD-GC-TOF-MS	Tenax TA/Carbotrap	RTX-5 amine column (30 m × 0.25 mm × 0.5 μm) (Restek)	4-Bromofluorobenzene	NIST	No
Schnabel et al. (2015)	VAP	Untargeted	MB	Tedlar^®^ bag	TD-GC-TOF-MS	Carbograph 1TD/Carbopack X	RTX-5MS (30 m × 0.25 mm × 1 μm)	No	NIST	No
Filipiak et al. (2015)	VAP	Targeted	AB	Glass syringe	TD-GC-MS	Carbotrap B 80 mg/Carbopack X 260 mg	PoraBOND Q (25 m × 0.32 mm × 5 μm) (Varian)	No	NIST	No
Ahmed et al. (2023)	VAP	Targeted	MB	BGS	TD-GC-MS	Tenax GR 200 mg	DB-5MS (30 m × 0.25 mm × 0.25 μm) (Agilent)	4-Bromofluorobenzene	na	Yes
Cen et al. (2023)	COVID-19	Untargeted	AB	ReCIVA	TD-GC×GC-TOF-MS	Tenax TA/Carbograph 5TD	DB-624 (30 m × 0.25 mm × 1.4 μm) (Agilent) 1D and DB-WAX column (5 m × 0.25 mm × 0.25 μm) (Agilent) 2D	Bromochloromethane, chlorobenzene-d5, and 1,4-dichlorobenzene-d4	NIST	Yes
Myers et al. (2023)	COVID-19	Untargeted	MB	Tedlar^®^ bag	TD-GC-TOF-MS	Tenax TA/Carbograph 1TD	nd	Toluene-D8	nd	Yes
Woollam et al. (2022a)	COVID-19	Untargeted	MB	Tedlar^®^ bag	SPME-GC-QTOF-MS	DVB/Car/PDMS	HP-5MS (30 m × 0.25 mm × 0.25 µm) (Agilent)	No	nd	Yes
Ibrahim et al. (2021)	COVID-19	Untargeted	MB	Tedlar^®^ bag	TD-GC-MS	Carbograph 1TD	DB-5MS (60 m × 0.25 mm × 0.25 μm) (Agilent)	Toluene-d8, phenanthrene-d10, and n-octane-d18	In-house library	Yes
Berna et al. (2021)	COVID-19	Targeted	MB	SamplePro FlexFilm Sample Bag	TD-GC×GC-TOF-MS	Tenax/Carbograph/Carboxen	Stabilwax (30 m × 250 μm × 0.25 μm) (Restek) 1D and Rtx-200MS (5 m × 250 μm × 0.1 μm) (Restek) 2D	Bromochloromethane, 1,4-difluorobenzene, chlorobenzene-D5, and 4-bromofluorobenzene	na	Yes

**TABLE 4:** Summary of group comparisons, statistical approaches, and identified VOCs in the studies focused on pneumonia and COVID-19. CAP, community-acquired pneumonia; CLZ, airway colonized; CN, culture-negative; CP, culture-positive; CTR, controls; FU COVID-19, follow-up samples of COVID-19 patients; HAP, hospital-acquired pneumonia; MVA, multivariate analysis; non-VAC, non-vaccinated; non-VAP, non-ventilator–associated pneumonia; PR CAP/HAP, probable community-acquired pneumonia/hospital-acquired pneumonia; PS CAP/HAP, possible community-acquired pneumonia/hospital-acquired pneumonia; UVA, univariate analysis; VAC, vaccinated; VAP, ventilator-associated pneumonia; VOCs, volatile organic compounds.

Reference	Pathology	Comparison	Statistical approach	Significant VOC	Detail
Van Oort et al. (2022)	VAP	CP (n = 52) vs. CN (n = 56)	UVA/MVA	1-Propenylbenzene (down), 2-bromophenol (down), 2-propenylbenzene (down), 2-methyldecane (up), 2,2-dimethyldecane* (up), 2,2,4,4-tetramethyloctane* (up), 2,6-difluorobenzaldehyde (up), 2,6,7-trimethyldecane (up), 3-methylheptane** (down), 6-methyl-5-hepten-2-one (up), cyclohexane (down), cyclohexanol (up), dimethyl sulfide* (up), enflurane (up), formaldehyde* (up), isopropylbenzene (down), m-di-tert-butylbenzene (down), and tetrahydrofuran (up)	*Significant VOCs in the UVA; **VOC reported in both platforms
Van Oort et al. (2017a)	CAP/HAP	PR CAP/HAP (n = 12) vs. PS CAP/HAP (n = 21) vs. CLZ (n = 13) vs. CTR (n = 47)/CP (n = 25) vs. CN (n = 68)	UVA/MVA	1-Pentanol* (down), 1-propanol** (down), 2-ethoxy-2-methyl-propane** (down), 2-methylcyclopentanone* (down), 5-methyl-2-heptanone* (down), acetone (down), carbon disulfide (down), cyclohexene (down), cyclohexanone* (down), hexafluoroisopropanol (down), methyl isobutyl ketone (down), and sevoflurane (down)	*VOCs colonized vs. non-colonized; **common VOCs PR CAP/HAP vs. CTR and colonized vs. non-colonized
Gao et al. (2016)	VAP	VAP (n = 20) vs. CLZ (n = 20) vs. CTR (n = 20)	UVA/MVA	1,5-Dimethyl-naphthalene (a), 1-undecene** (up), 2,6,10-trimethyl-dodecane* (up), 2-butyl-1-octanol* (up), 2-ethyl-1-hexanol (a), 5-methyl-5-propyl-nonane* (up), benzaldehyde (a), butylated hydroxytoluene (a), cyclohexanone (a), decanal** (up), ethanol (a), isoprene (a), longifolene** (up), n-nonylcyclohexane (a), nonanal* (up), tetradecane** (up), toluene (a), α-cedrene (a), and α-funebrene (a)	*Significant VOCs derived from *Acinetobacter baumannii*; **common VOCs *in vitro* and *in vivo*
Fowler et al. (2015)	VAP	CP (n = 15–26) vs. CN (n = 31–20)	MVA	2,6,11,15-Tetramethyl-hexadecane (up), 2-methyl cyclopentanone (down), 3-carene (up), ethanol (down), heptane (down), n-butyric acid 2-ethylhexyl ester (up), N-cyclohexyl-N′(2-hydroxyethyl)thiourea (down), and nonanal (up)	
Schnabel et al. (2015)	VAP	VAP (n = 32) vs. non-VAP (n = 68)	MVA	Acetone (down), acrolein (down), butane, 2-methyl (up), carane (up), dodecane (down), ethanol (up), ethylbenzene (up), tetrahydrofuran (down), heptane (up), isopropyl alcohol (down), tetradecanal (up), and tetradecane (up)	
Filipiak et al. (2015)	VAP	VAP (n = 22) vs. non-VAP (n = 6)	-	(E)-2-Butene (a), (Z)-2-butene (a), 1,3-butadiene (a), 1-undecene***** (a), 2-methyl-1-butene****** (a), 2-methylpropene (a), 2-pentanone*** (a), 3-methylbutanal (a), 3-methyl-1-butene**** (a), 4-heptanone** (a), acetaldehyde (a), acetic acid (a), acetonitrile*** (a), benzaldehyde (a), butane* (a), dimethyl sulfide*** (a), ethanol (a), ethyl acetate (a), hexanal (a), hexane****** (a), iso-butane****** (a), methacrolein (a), methanol (a), methyl vinyl ketone (a), propanal (a), and propane* (a)	*VOCs related to the course of infection with *Staphylococcus aureus*; **VOCs related to the course of infection with *Candida albicans*; ***VOCs related to the course of infection with *Escherichia coli*; ****VOCs related to the course of infection with *Haemophilus influenzae*; *****VOCs related to the course of infection with *Pseudomonas aeruginosa*; *******VOCs related to the course of infection with *Streptococcus pneumoniae*
Ahmed et al. (2023)	VAP	CP (n = 45) vs. CN (n = 59)	UVA	3-Methylbutanoic acid (up), 3-methylbutanal* (down/up*), acetone* (down), and indole** (up)	*Significant VOCs *P. aeruginosa* vs. other pathogen-positive culture; **significant VOC in patients with positive culture for bacteria that can metabolize tryptophan; down/up* different alterations between group comparisons
Cen et al. (2023)	COVID-19	VAC (n = 54) vs. non-VAC (n = 50)	UVA/MVA	2-Methyloctane* (down), 6-methyl-5-hepten-2-one (up), acetonitrile* (down), benzene (down), benzothiazole (up), cyclopentanone (up), hexanal* (down), methanesulfonyl chloride (up), and phenol* (down)	*VOCs in UVA
Myers et al. (2023)	COVID-19	COVID-19 (n = 69) vs. FU COVID-19 (n = 22) vs. CTR (n = 58) vs. HC (n = 21)	UVA/MVA	1-Propene, 1-(methylthio)-, (E)- (down), 2,2,4,6,6-pentamethylheptane**/*** (up), 2,2,4-trimethylpentane*/*** (up), 2-methyldecane*** (down/up*), 2-methylpentane**/*** (up), 2-pentanone*** (up), 3-methylheptane*/*** (up), allyl methyl sulfide*/*** (down/up*), cyclohexanone*** (up), dimethyl disulfide (down), ethyl acetate**/*** (up), heptanal (up), hexane**/*** (up), indole*** (up), methyl acetate**/*** (down), methyl butyrate**/*** (up), sulcatone*/*** (down/up*), α-phellandrene**/*** (down), and γ-terpinene**/*** (down)	*Common VOCs between comparisons: COVID-19 vs. FU COVID-19 and COVID-19 vs. CTR; **significant VOCs in COVID-19 vs. FU COVID-19; ***significant VOCs in the UVA; down/up* different alterations between group comparisons
Woollam et al. (2022a)	COVID-19	COVID-19 (n = 14) vs. HC (n = 12)	UVA/MVA	3,5,5-Trimethylhexanal (up), cedrene (up), and hexyl acetate (up)	
Ibrahim et al. (2021)	COVID-19	COVID-19 (n = 52) vs. CTR (n = 29)	MVA	1-Propanol** (up), 2,2-dimethyl-1-propanol* (a), 3-heptene* (a), 3,6-dimethylundecane (up), 4-ethenyl-1,2-dimethyl-benzene* (a), acetic acid methyl ester* (a), acetoin* (a), benzaldehyde** (a), camphene (up), cyclohexene* (a), iodobenzene (up), octanal* (a), pentadecane* (a), tetrachloroethylene* (a), and β-cubebene (up)	*VOCs identified in clinical suspicion comparison; **VOC identified in both comparisons (COVID-19 test and clinical suspicion)
Berna et al. (2021)	COVID-19	COVID-19 (n = 22) vs. CTR (n = 27)	UVA/MVA	2-Pentyl-furan (up), dodecane (up), heptanal (up), nonanal (up), octanal (up), and tridecane (up)	

**TABLE 5:** VOCs reported in pneumonia and COVID-19 (two or more studies). na, not applicable; ppbv, parts per billion by volume. *Downregulated in culture positive for *P. aeruginosa* ventilator-associated pneumonia patients and upregulated in culture positive for *S. aureus* ventilator-associated pneumonia patients; **downregulated in COVID-19 patients compared to follow-up COVID-19 patients and upregulated in COVID-19-positive patients compared to COVID-19-negative patients and healthy controls.

Pneumonia (CAP/HAP/VAP)									
No.	Compound name	Cas-N	Formula	Chemical class	Sign of alteration	Concentration (patients)	Concentration (controls)	Unit	Reference
1	Ethanol	64-17-5	C_2_H_6_O	Alcohol	Upregulated	na	na	na	Schnabel et al. (2015)
					Altered	na	na	na	Gao et al. (2016)
					Altered	na	na	na	Filipiak et al. (2015)
					Downregulated	na	na	na	Fowler et al. (2015)
2	3-Methylbutanal	590-86-3	C_5_H_10_O	Aldehyde	Altered	na	na	na	Filipiak et al. (2015)
					Downregulated/upregulated*	na	na	na	Ahmed et al. (2023)
3	Benzaldehyde	100-52-7	C_7_H_6_O	Aldehyde	Altered	na	na	na	Gao et al. (2016)
					Altered	na	na	na	Filipiak et al. (2015)
4	Nonanal	124-19-6	C_9_H_18_O	Aldehyde	Upregulated	na	na	na	Fowler et al. (2015)
					Upregulated	na	na	na	Gao et al. (2016)
5	Tetrahydrofuran	109-99-9	C_4_H_8_O	Ether	Downregulated	na	na	na	Schnabel et al. (2015)
					Upregulated	na	na	na	Van Oort et al. (2022)
6	Heptane	142-82-5	C_7_H_16_	Hydrocarbon (saturated)	Upregulated	na	na	na	Schnabel et al. (2015)
					Downregulated	na	na	na	Fowler et al. (2015)
7	Tetradecane	629-59-4	C_14_H_30_	Hydrocarbon (saturated)	Upregulated	na	na	na	Schnabel et al. (2015)
					Upregulated	na	na	na	Gao et al. (2016)
8	1-Undecene	821-95-4	C_11_H_22_	Hydrocarbon (unsaturated)	Upregulated	na	na	na	Gao et al. (2016)
					Altered	na	na	na	Filipiak et al. (2015)
9	2-Methylcyclopentanone	1120-72-5	C_6_H_10_O	Ketone	Downregulated	na	na	na	Fowler et al. (2015)
					Downregulated	na	na	na	Van Oort et al. (2017a)
10	Acetone	67-64-1	C_3_H_6_O	Ketone	Downregulated	na	na	na	Van Oort et al. (2017a)
					Downregulated	na	na	na	Schnabel et al. (2015)
					Downregulated	na	na	na	Ahmed et al. (2023)
11	Cyclohexanone	108-94-1	C_6_H_10_O	Ketone	Altered	na	na	na	Gao et al. (2016)
					Downregulated	na	na	na	Van Oort et al. (2017a)
12	Dimethyl sulfide	75-18-3	C_2_H_6_S	Sulfur-containing	Upregulated	na	na	na	Van Oort et al. (2022)
					Altered	0–101.5	na	ppbv	Filipiak et al. (2015)

COVID-19									
No	Compound name	CAS-N	Formula	Chemical class	Sign of alteration	Concentration (patients)	Concentration (controls)	Unit	First author/year
1	Heptanal	111-71-7	C_7_H_14_O	Aldehyde	Upregulated	na	na	na	Berna et al. (2021)
					Upregulated	na	na	na	Myers et al. (2023)
2	Octanal	124-13-0	C_8_H_16_O	Aldehyde	Upregulated	na	na	na	Berna et al. (2021)
					Altered	na	na	na	Ibrahim et al. (2021)
3	Methyl acetate	79-20-9	C_3_H_6_O_2_	Ester	Altered	na	na	na	Ibrahim et al. (2021)
					Downregulated	na	na	na	Myers et al. (2023)
4	6-Methyl-5-hepten-2-one	110-93-0	C_8_H_14_O	Ketone	Upregulated	na	na	na	Cen et al. (2023)
					Downregulated/upregulated**	na	na	na	Myers et al. (2023)

Additionally, several research groups participating in BreathDx had previously conducted studies focusing on CAP/HAP/VAP, one aiming at possible biomarkers for CAP/HAP (Van Oort et al., 2017b). In this study, patients were categorized based on their clinical suspicion, namely, probable CAP/HAP patients (high clinical suspicion), possible CAP/HAP patients (low clinical suspicion), colonized patients (without symptoms of pneumonia), and controls. Additionally, the entire patient cohort was divided into CP and CN. In the UVA, probable CAP/HAP patients and those who were CP presented 11 and 52 downregulated VOCs, respectively, and the classification models could discriminate between groups based on their clinical suspicion, and among CP and CN, even after LOOCV value (e.i. 0.730 and 0.690, respectively) AUC. While this study differed from the others, since they focused on CAP/HAP, several VOCs were shared, such as acetone, which was described by Ahmed et al. (2023), and 2-methylcyclopentanone, as reported by Fowler et al. (2015) (Table 5). The aforementioned study (Fowler et al., 2015) was performed by another research group involved in BreathDx, where ventilated ICU patients were sampled over their stay at five different time points to identify the VOCs that could be used to predict the risk of developing VAP. The model could separate CP and CN patients (sensitivity 98.0% and specificity 97.0%), and eight VOCs were selected as potential predictors (four downregulated and four upregulated). Several of these VOCs were common in different studies, such as ethanol, which was reported in a total of four independent studies (Table 5). In this regard, Schnabel et al. (2015) constructed an RF model based on 12 VOCs, such as ethanol, which correctly classified 74.2% of VAP and non-VAP patients (75.8% sensitivity, 73.0% specificity, and 0.870 AUC). Furthermore, when searching these VOCs in human and VAP-causing bacteria pathways, ethanol was found to be involved in six distinct pathways. Although ethanol seems to be a promising biomarker, its involvement in VAP development should be further studied, as this VOC participates in many physiological and pathological processes, such as OS, and its origin can be attributed to alcohol consumption.

Additionally, two studies focused on mVOCs previously detected *in vitro* from different cultures of pathogens associated with VAP. Filipiak et al. (2015) annotated 13 mVOCs in CP for *S. aureus* and 11 mVOCs in CP for *Candida albicans*. Considering the possible coexistence of VAP-causing pathogens, the study further aimed to explore and assess differential mVOCs that could potentially be associated with the progression of VAP caused by each pathogen. In this regard, 4-heptanone was found to be possibly related to *C. albicans*; propane and butane to *S. aureus*; acetanilide, 2-pentanone, and dimethyl sulfide to *E. coli*; 3-methyl-1-butene to *H. influenzae*; 1-undecene to *P. aeruginosa*; and n-hexane, iso-butane, and 2-methyl-1-butene to *S. pneumoniae*. Likewise, Gao et al. (2016) studied the presence of mVOCs in VAP patients, focusing on *A. baumannii*. For this purpose, *A. baumannii* VAP patients, *A. baumannii* colonized patients, and controls were compared, yielding 19 significant VOCs by UVA, 4 being also detected in *in vitro A. baumannii* cultures. Moreover, 8 of these VOCs were considered derived from *A. baumannii*, being able to differentiate *A. baumannii* VAP patients from colonized patients, as well as from controls (0.880 and 0.890 AUCs, respectively). Both studies reported three VOCs in common. Additionally, one VOC was reported by Filipiak et al. (2015). 3-Methylbutanal was also found in the BreathDx study (Ahmed et al., 2023) (Tables 5).

#### 3.3.2 COVID-19

In the past 3 years, COVID-19 has led to approximately 750 million confirmed cases and nearly 7 million deaths worldwide according to the WHO (WHO Coronavirus Dashboard, 2023). Several diagnostic tests were developed to contain the outbreak, such as reverse-transcription polymerase chain reaction (RT-PCR) for SARS-CoV-2 RNA detection in nasopharyngeal or oral swab samples, and antigen tests for spike (S) protein and nucleocapsid (N) protein detection. However, these tests have variable false-negative rates (Kucirka et al., 2020), with antigen tests being less sensitive and specific than RT-PCR (Scohy et al., 2020). Furthermore, these tests require multiple reagents, and in the case of RT-PCR tests, specialized equipment and trained technicians are required.

Despite the vaccination of over 13 million people worldwide (WHO Coronavirus Dashboard, 2023), COVID-19 remains an ongoing public health challenge. The potential emergence of more transmissible variants, changes in clinical symptoms, immune evasion (even in vaccinated individuals), and the possibility of reinfection are significant concerns. Additionally, distinguishing COVID-19 from other upper respiratory infections is crucial for isolation and transmission prevention. Consequently, breath tests, particularly in resource-limited settings, could offer a rapid means of diagnosing COVID-19.

Five studies that focused on COVID-19 are included in Tables 3 and 4. Two studies were conducted within a cohort of hospitalized patients. The targeted study by Berna et al. (2021) was performed in a cohort of pediatric patients. Six of the 84 targeted VOCs were upregulated in COVID-19 patients, which were further validated in an independent cohort. Moreover, the cumulative abundance of these six VOCs was evaluated as a diagnostic strategy (0.920 AUC, 91.0% sensitivity, and 75.0% specificity). Likewise, Ibrahim et al. (2021) identified six VOCs (seven features) that could discriminate COVID-19-positive test patients and COVID-19-negative test patients (0.836 AUC, 68.0% sensitivity, and 85.0% specificity), although the model based on 11 VOCs showed 0.659 AUC, discriminating patients based on clinical suspicion. In both comparisons, only two VOCs, 1-propanol and benzaldehyde, were common, suggesting that the specific metabolic alterations caused by COVID-19 are not necessarily related to symptomatology, especially if the symptoms are shared with other upper respiratory infections. These two studies presented one VOC in common, octanal (Table 5).

Conversely, two other studies were conducted on non-hospitalized COVID-19-positive patients. Woollam et al. (2022a) enrolled a cohort undergoing COVID-19 testing due to symptom onset, contact with symptomatic individuals, or mitigation testing. When COVID-19 patients were compared with HC, 41 VOCs were found to be significantly altered, mostly upregulated. Curiously, COVID-19 patients were divided into two subclasses based on their VOC profiles, one of which presented 4 of the 41 VOCs upregulated compared to the other subclass and HCs. Furthermore, the set of 41 VOCs could distinguish among groups with 96.0% accuracy, increasing to 100% when the 16 most significant VOCs were selected. The predictive classification model based on three VOCs (hexyl acetate, cedrene, and 3,5,5-trimethylhexanal) presented 100% sensitivity and 92.0% specificity value (e.i. 0.990) AUC. Lastly, 11 COVID-19 patients were sampled after recovery, and 34 VOCs recovered baseline levels, although five were still upregulated. When including this group in the final model, recovered COVID-19 patients clustered with controls and could be distinguished from COVID-19 patients with 90.0% accuracy. Myers et al. (2023) included patients presenting upper respiratory infections from two different ambulatory care settings. Moreover, some COVID-19 patients infected with Alpha, Beta, or Delta variants were resampled after 8–12 weeks (FU COVID-19). In the MVA, 12 VOCs could discriminate between COVID-19 and FU COVID-19 patients value (e.i. 0.825–0.862) AUC. Furthermore, COVID-19 patients and controls (COVID-19-negative test patients presenting symptoms) could be distinguished by 11 VOCs, which were further validated in an independent cohort value (e.i. 0.960, 80.0% sensitivity, and 90.0% specificity) AUC. From both comparisons, four common VOCs (2,2,4-trimethylpentane, sulcatone, allyl methyl sulfide, and isobutyric acid) were identified.

Additionally, Cen et al. (2023) investigated the metabolic reprogramming triggered by the inactivated COVID-19 vaccine, comparing the VOC profiles of COVID-19 vaccinated and unvaccinated subjects. The discriminative model based on nine VOCs (from 21 identified in both UVA and MVA), which included 6-methyl-5-hepten-2-one already found by Myers et al. (2023) (Table 5), exhibited 94.4% overall accuracy, 91.3% sensitivity, and 98.6% specificity value (e.i. 0.995) AUC. Furthermore, the examination of the biomarkers’ metabolic pathways demonstrated that the protective metabolic regulation induced by the vaccine influences enzymatic activity and microbial metabolism within the lungs, liver, and gastrointestinal tract.

### 3.4 Searching for pathology-specific VOCs in human exhaled breath

The search for potential biomarkers in exhaled breath is challenging due to the substantial variability in the concentration of VOCs. This variability is due to metabolic activity but also depends on lifestyle choices (smoking, exercise, diet, etc.) and/or exposure to exogenous factors, such as pollutants and other environmental compounds, among others. Despite this challenge, numerous studies have focused on identifying specific VOCs associated with a wide range of pathologies. Nevertheless, the results of these studies should be interpreted with caution. In most instances, the origin of these VOCs remains unidentified, which can lead to false discoveries.

**FIGURE 4:**
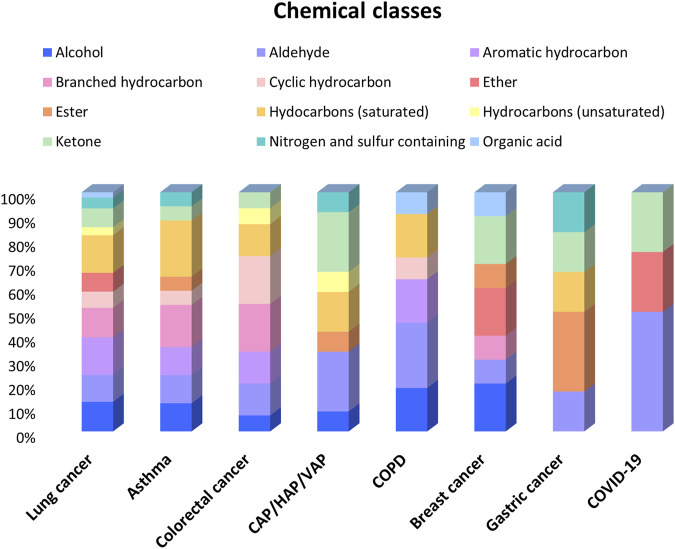
Bar chart of the chemical classes of VOCs (referred by two or more studies) reported in each pathology. CAP, community-acquired pneumonia; COPD, chronic obstructive pulmonary disease; HAP, hospital-acquired pneumonia; VAP, ventilator-associated pneumonia.

The VOCs included in Tables 2, 5; Supplementary Table S3, as classified by Drabińska et al. (2021), are illustrated in Figure 4. As noted, the analysis of exhaled breath covers a wide range of chemical species, although the distribution of these is variable across pathologies. Aldehydes are the most abundant, mainly derived from alcohol metabolism in the liver or the reduction of hydroperoxides during lipid peroxidation (Murray et al., 2009; Hakim et al., 2012), although aldehydes can also come from cigarette smoking or tobacco components’ detoxification by cytochrome P450 (Furge and Guengerich, 2006; Papaefstathiou et al., 2020). This chemical group is predominant in CAP/HAP/VAP, COVID-19, and COPD.

Ketones are also strongly represented in CAP/HAP/VAP and COVID-19, mainly resulting from the liver’s synthesis of ketone bodies (acetoacetate, acetone, etc.) during conditions like diabetes, fasting, or alcoholism, formed through the metabolism of proteins and/or as secondary products of lipid peroxidation (Vaz and Coon, 1987; Murray et al., 2009). Remarkably, a significant proportion of ethers is observed in COVID-19 and BC, although its origin is commonly attributed to exogenous sources. Other abundant compounds in BC are alcohols, which may come from the gastrointestinal tract or are formed through the hydrocarbon’s metabolism or lipid peroxidation (Ortiz De Montellano, 2010; Ratcliffe et al., 2020).

Additionally, hydrocarbons are widely reported in exhalates, primarily saturated, aromatic, branched and cyclic. These compounds are highly represented in LC, asthma, and CRC. Hydrocarbons are mainly produced by lipid peroxidation, in an abnormal metabolic state. Branched-chain hydrocarbons may be of an endogenous origin from bacterial metabolism (Ratcliffe et al., 2020).

The concept of the exposome is gaining popularity, encompassing not only external exposures (chemical agents, radiation, etc.) and associated physiological responses but also internal sources, such as microbiota, and “psychosocial components” (Vineis et al., 2020). Several studies have focused on identifying metabolites related to the exposome, such as the database developed by Neveu et al. (2023), which includes microbial metabolites and is supported by evidence on their origin, and the method developed by González-Domínguez et al. (2020) for exposome research. Furthermore, the effect of the exposome on human health has been widely studied (Morales et al., 2022). Nevertheless, many metabolites associated with the exposome overlap with those produced by human cells/tissues, making it a difficult task to establish what can be considered truly endogenous. This issue is especially challenging for VOCs detected in exhaled breath, since the pulmonary tract is closely associated with environmental exposure.

**FIGURE 5:**
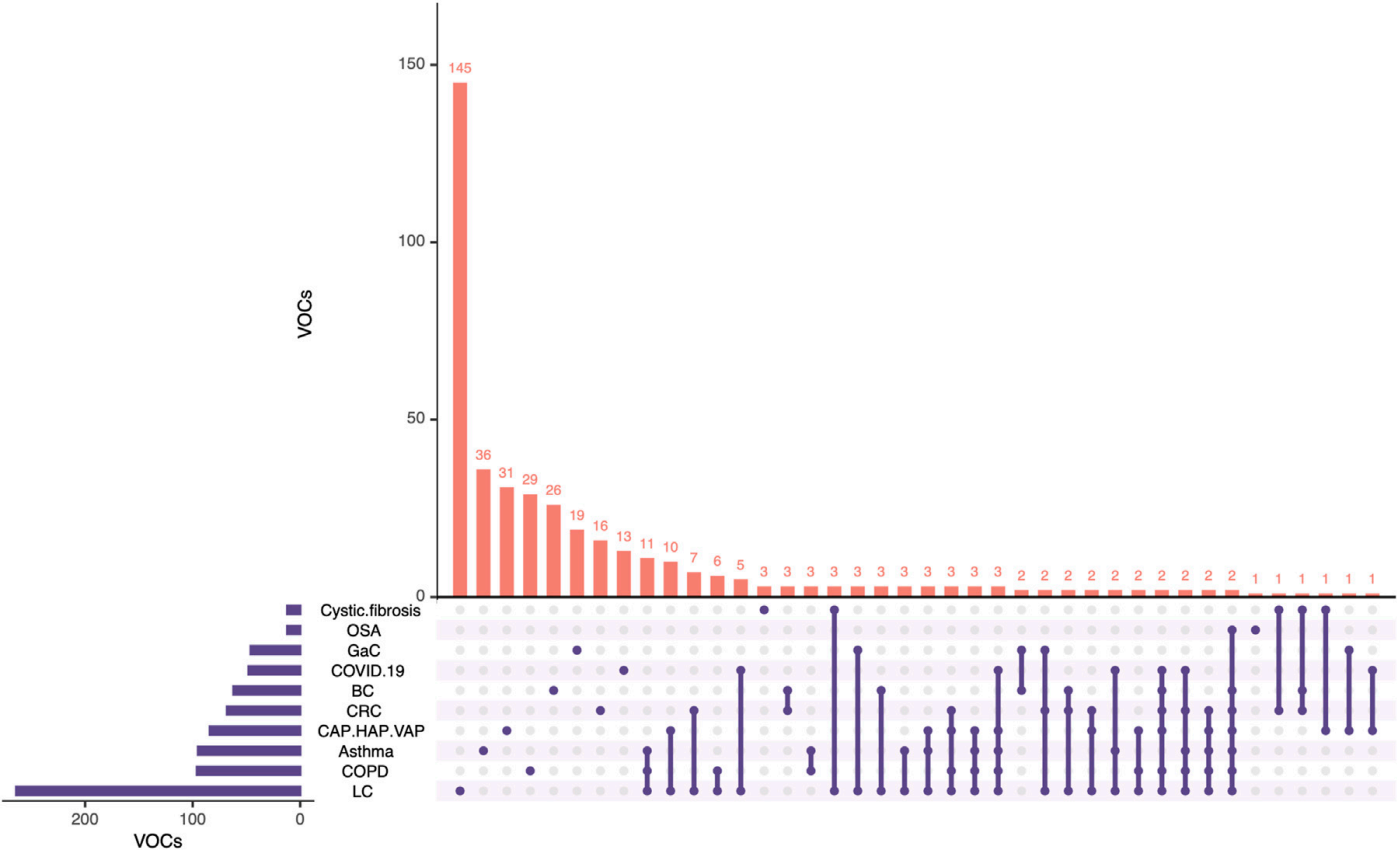
Upset plot of the VOCs reported in the reviewed studies. BC, breast cancer; CAP, community-acquired pneumonia; COPD, chronic obstructive pulmonary disease; CRC, colorectal cancer; GaC, gastric cancer; HAP, hospital-acquired pneumonia; LC, lung cancer; OSA, obstructive sleep apnea; VAP, ventilator-associated pneumonia. Created with RStudio (Conway et al., 2017).

The full list of reported VOCs was used to identify pathology-specific compounds (Figure 5). The overlapping VOCs may come from exogenous sources (exposome), such as cigarette smoking, environmental pollution, or diet, as well as shared endogenous origins like the ones derived from OS or common VOCs found in breath, such as isoprene and acetone.

Regarding pathology-specific possible biomarkers, several unique VOCs were found (Figure 5), especially in LC, according to the literature reviewed herein. Furthermore, the obtained list of unique pathology-specific VOCs was submitted to searches on KEGG and BioCyc databases with the aim of excluding the VOCs mainly coming from exogenous sources. Upon exclusion of such exogenous VOCs, the final list of pathology-specific possible biomarkers is compiled in Table 6. It is worth mentioning that although these candidate biomarkers might provide useful information, further research is required to establish associations with metabolic alterations in each pathology, as well as to discern between the VOCs that may be related to the exposome and the ones that are truly endogenous.

**TABLE 6:** Pathology-specific proposed biomarkers. BC, breast cancer; CAP, community-acquired pneumonia; COPD, chronic obstructive pulmonary disease; CRC, colorectal cancer; GaC, gastric cancer; HAP, hospital-acquired pneumonia; LC, lung cancer; OSA, obstructive sleep apnea; VAP, ventilator-associated pneumonia. *Possible exogenous origin.

Pathology	PubChem ID	Compound*
LC	650	2,3-Butanedione
LC	261	Butanal
LC	1032	Propionic acid
LC	984	Hexadecanal
GaC	225936	2,3-Butanediol
CRC	243	Benzoic acid
CRC	2969	Decanoic acid
BC	10413	4-Hydroxybutanoic acid
Asthma	11005	Myristic acid
Asthma	637540	2-Hydroxycinnamic acid
COPD	2879	p-Cresol
OSA	999	Phenylacetic acid
CAP/HAP/VAP	10430	Isovaleric acid

Furthermore, the correct metabolite identification is a highly important aspect in metabolomics, and different levels can be distinguished based on the reliability of the identification. In this regard, the Metabolomics Standard Initiative (MSI) levels can range from 1 to 4, level 1 being the most rigorous (Sumner et al., 2007).

In the case of LC, four VOCs from Table 6 were reported as potential biomarkers in at least two different studies (Supplementary Table S3). In this regard, 2,3-butanedione and butanal (both MSI level 1) were found to be upregulated. The remaining candidate biomarkers were reported as altered; thus, the trend of their levels should be assessed. Propionic acid (MSI levels 1 and 2) was reported as upregulated and downregulated in two different studies; therefore, it is not an adequate candidate due to the contradictory findings. The remaining metabolites included in Table 6 were reported only once, requiring further study for their use as pathology-specific biomarkers. Additional candidate biomarkers whose endogenous origin has not been established include 2-nonenal (MSI levels 1 and 2), 3-methylhexane (MSI levels 1 and 2), butanal (MSI level 1), pentane (MSI level 1), and propylene (MSI levels 1 and 2) for LC, all of which are reported several times and show a trend toward increased levels; acrylonitrile (MSI level 1) for GaC is reported as upregulated in two independent studies; methacrylic acid (MSI level 2) for BC presents decreased levels; and 1,2-dimethylcyclohexane (MSI level 2) for asthma and 2-methylcyclopentanone (MSI level 2) for CAP/HAP/VAP are reported as downregulated (Tables 2, 5; Supplementary Table S3).

## 4 Methodologies

**FIGURE 6:**
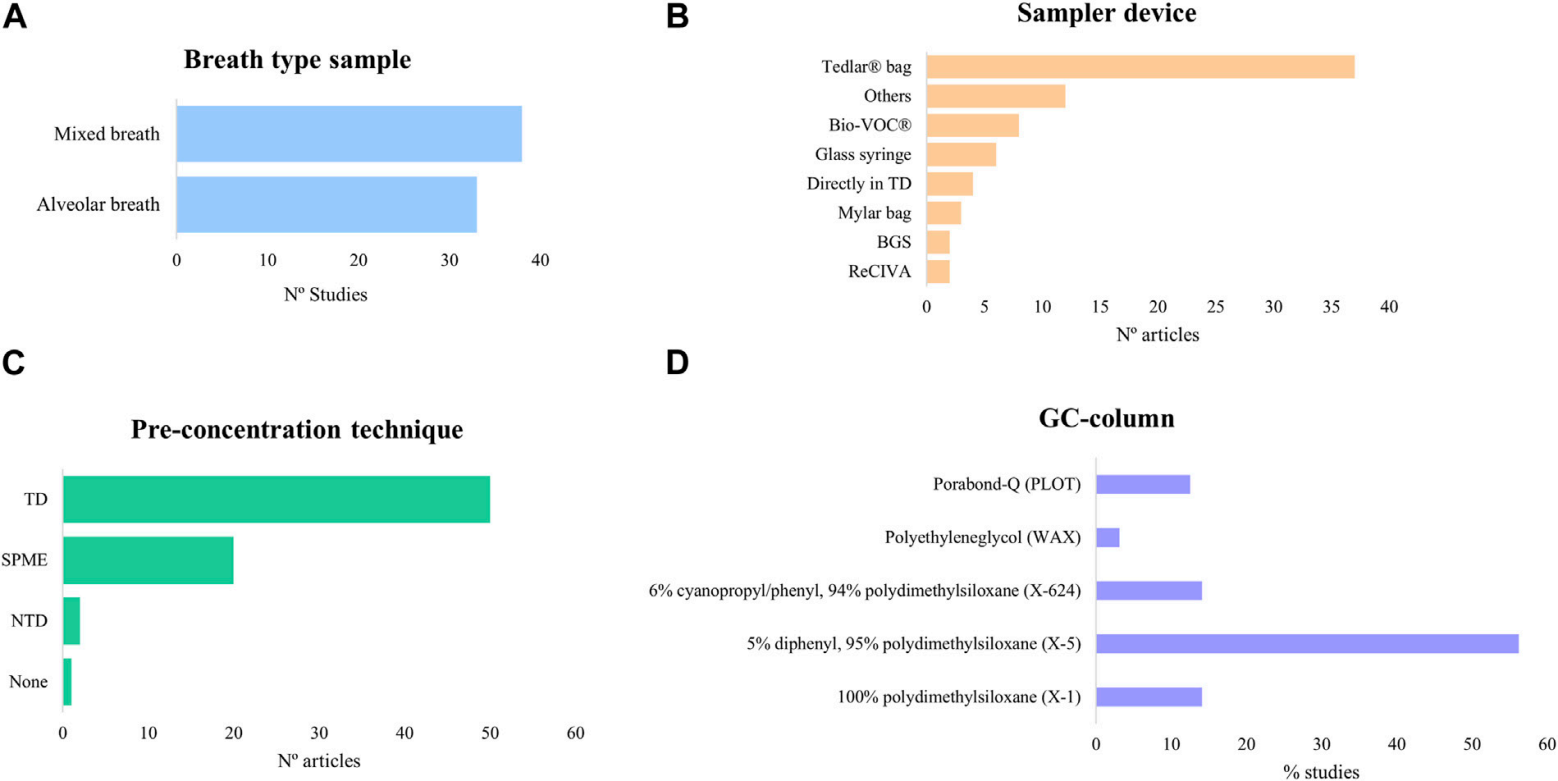
Bar charts of the exhaled breath sampling and pre-concentration methods from the reviewed studies. **(A)** Breath-type sample; **(B)** sampler device; **(C)** pre-concentration technique; **(D)** GC-column. BGS, breath-gas sampler; GC, gas chromatography; NTD, needle-trap device; SPME, solid-phase microextraction; TD, thermal desorption tube.

The methodologies used for breath sampling and VOCs' pre-concentration and separation in the reviewed studies are presented in Tables 1, 3; Supplementary Table S1 and illustrated in Figure 6.

### 4.1 Exhaled breath sampling

Breath samples were categorized in mixed or alveolar breath, as late expiratory breath sampling was not specified in any study, being usually confused with alveolar breath. The lack of distinction may be due to the absence of standardized protocols or guidelines for the collection of late expiratory breath. Devices that discard dead space air may not ensure true alveolar breath sampling; therefore, only those with CO_2_ or pressure sensors should be used for this type of sample.

Exhaled breath sampling devices can collect from a few milliliters to 10 L, which depends not only on the device's capacity but also on the fraction of breath sampled, since alveolar breath represents approximately 350 mL of the total expiratory volume. Even though breath samples can be taken from a single expiration or multiple expirations, VOC profiles can vary from breath to breath (Khoubnasabjafari et al., 2022), and the concentrations differ significantly in hypoventilation, hyperventilation, and normal ventilation (Cope et al., 2004).

The selection of sample type depends on the compounds of interest. When studying endogenous VOCs, late expiratory or alveolar breath are preferred, the latter being more convenient due to the higher concentration of VOCs and reduced contamination (Miekisch et al., 2008). However, as depicted in Figure 6A, mixed breath was the most analyzed sample type, probably because the devices used for this type of sample are more affordable and easier to use. However, due to the increasing interest in endogenous VOCs, alveolar breath was analyzed in a significant number of studies, especially in those focused on cancer.

The most common collection device was Tedlar^®^ bags (Figure 6B), consistent with previous reviews (Lawal et al., 2017; Westphal et al., 2023). While these bags are subject to contamination and have limited sample storage time, they are affordable and reusable, with several cleaning protocols available (Westphal et al., 2023). Additionally, devices such as Bio-VOC^®^, breath-gas sampler, and ReCIVA are gaining popularity, although they are not as widely used.

Sampling methodologies are organized per type of pathology in this section as the severity of the pathology may justify specific approaches.

#### 4.1.1 Cancer

In LC, 14 studies analyzed alveolar breath, while the remaining 9 analyzed mixed breath. The alveolar breath samples were collected using various techniques, such as Bio-VOC^®^, BCA, Tedlar^®^/Mylar bags, or other devices (analytical barrier bag and breath reservoir). For mixed breath samples, Tedlar^®^/Mylar bags were predominantly used, and some studies employed self-developed devices and glass bulbs.

In GaC, alveolar breath was the main type, sampled either with Mylar/Tedlar^®^ bags, GaSampler collection bags (QuinTron), or a custom-built in-house breath sampler. One study used gas-tight syringes for mixed breath sampling. In CRC, two studies sampled mixed breath using Tedlar^®^ bags, and the remaining alveolar breath employed ReCIVA^®^ or other devices [GaSampler Collection Bag (QuinTron) and gas-tight syringes]. In BC, all studies collected alveolar breath, using various devices such as gas-tight syringes, Tedlar^®^ bags, and Bio-VOC^®^.

#### 4.1.2 Other pulmonary pathologies

In asthma, all the studies collected mixed breath samples using Tedlar^®^ bags, except for one that analyzed alveolar breath and used an ABS. In COPD, alveolar breath and mixed breath were selected with equal frequency. The samplers employed were either Tedlar^®^ bags or stainless steel tubes for mixed breath, and Bio-VOC^®^ or glass syringes for alveolar breath. Furthermore, in one study, alveolar breath was also collected directly into the pre-concentration device. In OSA, alveolar and mixed breath samples were sampled with Bio-VOC^®^ and Tedlar^®^ bags, respectively, and in CF, mixed breath was collected using Tedlar^®^ bags.

#### 4.1.3 Infectious pathologies

In CAP/HAP/VAP, one of the targeted studies sampled alveolar breath employing glass syringes, while the remaining studies analyzed mixed breath samples. Since the patients were intubated and mechanically ventilated, most of the sampling was performed directly in sorbent tubes, except for three studies that employed either Tedlar^®^ bags or a breath-gas sampler. In COVID-19, most of the studies analyzed mixed breath, collecting the sample either using Tedlar^®^ bags or SamplePro FlexFilm Sample Bags. Only one study sampled alveolar breath employing ReCIVA.

### 4.2 Pre-concentration techniques

TD were the most utilized pre-concentration technique, followed by SPME (Figure 6C), consistent with previous reviews (Lawal et al., 2017; Westphal et al., 2023). The widespread use of TD can be attributed to their suitability for long-term sample storage, ease of transport, and the stability of the entrapped compounds. However, SPME requires smaller sample volumes and is less affected by humidity, offering a similar extraction range to TD. Notably, NTD was used in only a few studies, despite presenting aspects of both SPME and TD.

The choice of the sorbent material depends on the chemical nature of the analytes of interest, including polarity and molecular weight (MW). The most used SPME fiber coating material was Car/PDMS (mainly 75 µm thickness), followed by DVB/Car/PDMS, and lastly, PDMS 100 µm and PDMS/DVB (Tables 1, 3; Supplementary Table S1). These fibers are bipolar, except for PDMS (non-polar), with the latter compromising the extraction of polar metabolites (Vas and Vékey, 2004). The wider use of Car/PDMS fiber could be due to the ability of this coating material to better extract low-molecular-weight volatiles (MW 30–225 g/mol) compared to PDMS 100 µm (MW 60–275 g/mol) and DVB/PDMS (MW 80–300 g/mol) (Lawal et al., 2017). However, in a recent study (Schulz et al., 2023), DVB/Car/PDMS turned out to be the most adequate for untargeted studies compared to Car/PDMS and PDMS fibers due to the higher number of extracted VOCs and the stronger overall GC-MS signal.

Regarding TD, Tenax TA is the most used sorbent material (Tables 1, 3; Supplementary Table S1). Although this material captures heavy- and less-volatile compounds, its low affinity to water and the broad sampling range (C6–C30) makes it adequate for untargeted analysis (Wilkinson et al., 2020). Similarly, a few studies have opted for Tenax GR. Other sorbent materials encompass carbon black adsorbents, such as Carbograph 1TD, Carbograph 5TD, Carbopack X, Carbopack B, and Carbotrap. Additionally, carbon-based materials like Carboxen were employed. These alternatives offer a narrower range (C3–C20 and C2–C5), although they facilitate the capture of low-molecular-weight and more volatile compounds (Lawal et al., 2017; Westphal et al., 2023). Many studies have employed multi-bed sorbents, with the most prevalent being Carbograph 1TD/Carbopack X. Furthermore, combinations of the aforementioned materials have been used, such as Tenax TA/Carbograph 1TD and Tenax TA/Carbograph 5TD, both of which have been considered for exhaled breath analysis in previous studies (Wilkinson et al., 2020). Additional combinations, such as Tenax/Carbograph/Carboxen and Carbotrap B/Carbopack X, were also employed.

### 4.3 GC-MS methods

Considering that the main objective of most studies discussed in the present review was to obtain a snapshot of the VOC content in the breath samples, and also the analysis conditions and the pre-concentration techniques. Conditions of the GC-MS method such as the injector mode, chromatographic column, and type of detector employed for analysis are as important as the preceding pre-concentration technique.

Regarding GC injector parameters, from the 70 works reviewed, an astonishing 61% do not detail the type of injector or injection employed. Such a number is alarming given the fundamental difference between injecting gaseous and liquid samples. In fact, for gaseous injections choosing an injector glass liner of smaller inner diameter would provide a more efficient transfer of analytes onto the GC column, thus yielding more peak capacity efficiency. Nonetheless, of the 39% of works that mentioned the employment of a splitless/split injector, none mentioned the dimensions of the injector glass liner diameter employed. Out of these split/splitless injections, 70% of the injected samples are in the splitless mode, which would indeed be expected for pre-concentration techniques such as direct TD and SPME.

When it comes to GC columns as presented in Figure 6D (compiling the information from Tables 1, 3; Supplementary Table S1), over 50% of the studies herein reviewed employed 5% diphenyl/95% polydimethylsiloxane phases, here named as X-5 columns (DB-5, RTX-5, SLB-5, HP-5, VF-5, etc.). This type of stationary phase is considered the most versatile owing to the slight polarity imparted by the substitution of 5% of dimethyl groups by diphenyl. This addition also makes this stationary phase suitable for the separation of unsaturated hydrocarbons and aromatic compounds. Conversely, this stationary phase should not be the first choice regarding the analysis of VOCs. In fact, despite its slight polarity, it does not provide sufficient retention and efficiency for the separation of low-molecular-weight polar VOCs, such as alcohols, aldehydes, and organic acids.

The second most used types of GC stationary phase are X-624 and X-1, each being employed in 14% of the studies presented in Tables 1, 3; Supplementary Table S1. The X-624 stationary phase (DB-624, VP-624, SLB-624, etc.) consists of polydimethylsiloxane with 6% of the dimethyl substituted by cyanopropyl and phenyl groups. Therefore, as expected, this is a low-polarity phase, though of higher polarity than its X-5 counterparts. A key characteristic of these columns is the thickness of the stationary phase. While most X-5 columns employ stationary phases of 0.25 µm thickness, X-624 columns are coated with, at least, 1.4 µm of the stationary phase. Therefore, in addition to its chemistry allowing for better selectivity, there is also a considerable gain in retention for low-molecular-weight polar VOCs and a wider range of VOC classes could be successfully analyzed in breath samples. In fact, X-624 stationary phases are the most suitable for VOC analyses, as it is the official stationary phase for a variety of Environmental Protection Agency (EPA) methods dealing with VOCs. X-1 columns (DB-1, RTX-1, SLB-1, HP-1, VF-1, etc.) contain 100% polydimethylsiloxane, the most non-polar stationary phase. Similar to X-5 types of phases, this stationary phase does not provide sufficient selectivity for the separation of small and polar VOCs even when employing thicker phases.

Given the importance of aldehydes and alcohols in the studies herein reviewed, as presented in Figure 4, it may be surprising that only two studies employ polyethylene glycol (WAX) stationary phases, as they are highly selective for polar compounds such as alcohols. A plausible explanation in this type of application might be related to the presence of water in the breath sample. WAX columns are particularly sensitive to moisture in the sample, which may lead to the degradation of this stationary phase.

While all other GC columns mentioned here encompass wall-coated open tubular (WCOT) columns, porous layer open tube (PLOT) columns are highly retentive and, therefore, are primarily employed for the analysis of very low boiling point compounds that are gaseous at room temperature, such as sulfides. Applied in 12% of the studies herein reviewed, Q-PLOT columns are non-polar, as they employ 100% divinylbenzene as adsorbent, therefore imparting great selectivity and retention for low-molecular-weight hydrocarbons.

Most of the studies included in Tables 1, 3; Supplementary Table S1 were performed by one-dimensional (1D) GC-MS. Additionally, five studies applied comprehensive bidimensional gas chromatography (GC×GC). The most common GC×GC setup employs an orthogonal mechanism of separation, using two sequential GC columns with stationary phases of different polarities, with a modulator between them. In short, a narrow band eluting from the first dimension (1D) column is collected and focused on the modulator, and then sent to the second dimension (2D) column (which is much shorter than the 1D). In this way, for example, compounds of similar boiling points coeluting on the 1D could be separated according to their polarity differences on the 2D. This technique offers significant advantages: the extended peak capacity improves peak space separation and allows the detection of coeluting compounds that could be missed by conventional 1D-GC. Moreover, given the acquisition speed required by the narrow bands eluting from the 2D in GC×GC, this technique is often hyphenated with MS detectors with rapid MS analyzers such as TOF, providing also higher sensitivity than that obtained by 1D-GC employing single quadruple MS. As an example of the advanced capacities of GC×GC-TOF-MS, Caldeira et al. (2012) could detect eight-fold more compounds, especially alkanes, alkenes, aldehydes, and ketones, and the concentration range achieved was lower than that of a previous study performed with GC-qMS. Four out of the five studies presented the traditional apolar × polar configuration, employing stationary phases like X-624 and X-5 in the first dimension (1D) and a polar polyethylene glycol-based (WAX) phase in the second dimension (2D) (Caldeira et al., 2012; Pesesse et al., 2019; Schleich et al., 2019; Cen et al., 2023). This combination reduces the interaction of water with the polar stationary phase and provides information on both the volatility (1D) and polarity (2D) of the compounds in the sample (Wilde et al., 2019). Interestingly, Berna et al. (2021) employed a polar × polar setup, using a WAX-based column in the 1D and a trifluoropropylmethyl polysiloxane (RTX-200) column in the 2D. The 2D stationary phase offers a unique selectivity for electron-rich molecules and resolves compounds that could not be resolved by the Wax 1D column. The limited use of this technology can be attributed to the high costs of instrumentation, especially for cryo-based modulators that are the most adequate for applications such as breath analysis due to their ability to successfully trap very volatile compounds. Moreover, method optimization in GC×GC is not as straightforward as in 1D-GC, hence requiring specialized personnel from method development to data process and interpret (Pesesse et al., 2019).

Moreover, 20 studies have employed high-resolution mass spectrometers (TOF-MS), of which more than half were included in studies on other pulmonary diseases (Tables 1, 3; Supplementary Table S1). The high-resolution approach offers notable advantages, especially when performing an untargeted study. In this regard, sensitivity and selectivity are improved compared to the low-resolution approach. The spectral libraries used for compound identification include accurate mass, which further allows for the enhanced structural elucidation of unknown compounds. Nonetheless, the use of this equipment is more complex; data processing requires more time and space and the price is higher (Rey-Stolle et al., 2021).

### 4.4 Quality control

In breath analysis, as in any metabolomics study, evaluating the quality of the obtained data is crucial. This is essential not only for obtaining reproducible results but also to ensure that the differences observed between groups are attributable to the composition of the samples rather than analytical/instrumentation variations. Such assurance involves the analysis of blanks and the use of internal standards among other strategies detailed below (Dudzik et al., 2018).

The analysis of blanks allows for the identification of contaminants (e.g., Tedlar^®^ bag contaminants) and artifacts (e.g., polydimethylsiloxanes), and its elimination from the data matrix, avoiding false discoveries. The main blanks include collecting device blank, air blank, and vial/tube blank (Westphal et al., 2023). In this regard, several studies have reviewed and conducted analyses of ambient air, yet a considerable number of studies do not include this step, such as that of Gao et al. (2016), Aoki et al. (2017), Van Vliet et al. (2017), and Saidi et al. (2020). Moreover, the inclusion of the remaining blanks is not specified in most of the studies. The injection of a standard mixture over the sequence is recommended, although just a few studies include it in their workflow (Schleich et al., 2019; Koureas et al., 2021).

Despite being of utmost importance to obtain precise results, the addition of an internal standard (IS) to the samples was performed in few studies (Basanta et al., 2012; Corradi et al., 2015; Fowler et al., 2015; Berna et al., 2021; Ibrahim et al., 2021; Van Oort et al., 2022; Ahmed et al., 2023; Cen et al., 2023; Myers et al., 2023) (Tables 1, 3; Supplementary Table S1). Likewise, hexamethylcyclotrisiloxane, a desorption tube bleeding compound, was used as an internal reference compound (Callol-Sanchez et al., 2017; Jareño-Esteban et al., 2017; Muñoz-Lucas et al., 2020). The ISs used include not isotopically labeled compounds such as 1,4-difluorobenzene, 2-methylpentanal, 4-bromofluorobenzene, bromochloromethane, and stable isotopically labeled ones, such as acetone-d8, 1,4-dichlorobenzene-d4, chlorobenzene-d5, bromobenzene-d5, hexane-d14, n-heptane-d16, n-octane-d18, phenanthrene-d10, styrene-d8, toluene-d8, and xylene-d10. Additionally, ISs can be used for data normalization. However, most of the studies reviewed did not add an IS nor did they specify how the data normalization is performed. It is worth mentioning that for a pre-concentration technique based on equilibrium, such as SPME, isotopically labeled ISs present by far the best precision (and accuracy). In fact, inconsistencies during sampling can be normalized as the IS extraction will be influenced to the same extent as the analyte of interest.

Generally, to ensure quality in the analysis of exhaled breath samples by GC-MS, and therefore reliable findings, several key quality assurance and quality control measures are essential. These measures aim to guarantee the accuracy, reproducibility, and reliability of the entire analytical results and are as follows (Li et al., 2019; Becker, 2020; Westphal et al., 2023):

• Calibrants and reference materials: Calibrants and reference materials containing volatile marker compounds are used to establish detector stability, known detection limits, and instrument calibration. These materials should be available consistently during clinical trials and routine applications.
• Training and test samples: For the reproducible identification of specific odor patterns, the availability of appropriate sets of training and test samples is essential. These samples aid in electronic nose-based or canine-based identification methods.
• Standardization and harmonization: Standardization of breath sampling procedures is crucial to minimize inter-observer and intra-observer errors. Researchers involved in breath sampling should undergo certification to ensure uniform and accurate collection processes. Standardization also involves monitoring room air for potential VOC contamination.
• Instrument calibration: Regular instrument calibration using internal standards, such as stable isotope-labeled compounds, enhances QC/QA efforts. It helps in tracking instrument performance and ensuring the accuracy of results.
• Blank analysis: To identify and remove contaminants, blank analyses are essential. These blanks include air blanks, system blanks to identify instrument artifacts, and blanks to account for chemical backgrounds originating from sampling materials like Tedlar^®^ bags.
• Spiking samples with internal standards of known concentrations and different retention times aids in data normalization and enhances data quality.
• Inter-laboratory comparisons: For diagnostic purposes, it is important to compare data obtained from different laboratories and methods. It helps assess the reproducibility and relevance of potential biomarkers.

In summary, ensuring quality in exhalation analysis by GC-MS involves a comprehensive approach that encompasses quality control, standardization, instrument calibration, and data management. All these measures must be implemented without any exception in clinical applications where breath analysis holds potential for disease diagnosis and monitoring.

## 5 Conclusion

This comprehensive review aims to investigate the potential of GC-MS analysis of VOCs in breath as biomarkers for severe pathologies, such as cancer, pulmonary diseases, and infectious diseases. Critical aspects of the workflow are thoroughly considered and discussed, encompassing the type of exhaled breath, collection devices, pre-concentration techniques, and analysis, as well as the experimental designs, statistical analysis, identification strategies, and proposed potential VOCs biomarkers.

Tedlar^®^ bags and TD are by far the most extended for collection and pre-concentration, respectively. However, the choice of the type of breath sample was more diverse, spanning between mixed and alveolar breath, a critical consideration when aiming to accurately compare and establish levels of endogenous VOCs. Despite the wealth of studies, the conspicuous lack of standardization in the methodological approach and the scarce absolute quantitation of potential biomarkers delay their transference to clinics. Additionally, relatively small cohorts with only a limited model validation in an independent cohort, along with the lack of consensus in altered findings among different studies hindered the identification of a single pathology-specific VOC. A deeper understanding of the endogenous origin of VOCs is imperative to fully grasp the significance of each VOC in discriminating between healthy and pathological states.

Overall, this review underscores the substantial potential of VOCs as biomarkers in health and pathology. Nonetheless, to fully harness this potential, it is crucial to address the lack of standardization in methodological approaches, include larger and well-defined cohorts, and validate models in independent cohorts. As we delve deeper into the complexities of VOCs in exhaled breath, we are poised to advance personalized and non-invasive diagnostic strategies that can revolutionize the detection and management of the pathology, ultimately benefiting public health.

## References

[B1] Acharige M. J. T. Koshy S. Ismail N. Aloum O. Jazaerly M. Astudillo C. L. (2018). Breath-based diagnosis of fungal infections. J. Breath. Res. 12, 027108. 10.1088/1752-7163/aa98a1 29109305

[B2] Ahmed W. M. Fenn D. White I. R. Dixon B. Nijsen T. M. E. Knobel H. H. (2023). Microbial volatiles as diagnostic biomarkers of bacterial lung infection in mechanically ventilated patients. Clin. Infect. Dis. 76, 1059–1066. 10.1093/cid/ciac859 36310531 PMC10029988

[B3] Alnimr A. (2023). Antimicrobial resistance in ventilator-associated pneumonia: predictive microbiology and evidence-based therapy. Infect. Dis. Ther. 12, 1527–1552. 10.1007/s40121-023-00820-2 37273072 PMC10240484

[B4] Alonso M. Sanchez J. M. (2013). Analytical challenges in breath analysis and its application to exposure monitoring. TrAC - Trends Anal. Chem. 44, 78–89. 10.1016/j.trac.2012.11.011

[B5] Altomare D. F. Di Lena M. Porcelli F. Travaglio E. Longobardi F. Tutino M. (2015). Effects of curative colorectal cancer surgery on exhaled volatile organic compounds and potential implications in clinical follow-up. Ann. Surg. 262, 862–866. 10.1097/SLA.0000000000001471 26583677

[B6] Altomare D. F. Di Lena M. Porcelli F. Trizio L. Travaglio E. Tutino M. (2013). Exhaled volatile organic compounds identify patients with colorectal cancer. Br. J. Surg. 100, 144–150. 10.1002/bjs.8942 23212621

[B7] Altomare D. F. Picciariello A. Rotelli M. T. De Fazio M. Aresta A. Zambonin C. G. (2020). Chemical signature of colorectal cancer: case-control study for profiling the breath print. BJS Open 4, 1189–1199. 10.1002/bjs5.50354 32990407 PMC8444279

[B8] Amal H. Leja M. Broza Y. Y. Tisch U. Funka K. Liepniece-Karele I. (2013). Geographical variation in the exhaled volatile organic compounds. J. Breath. Res. 7, 047102. 10.1088/1752-7155/7/4/047102 24184568

[B9] Amal H. Leja M. Funka K. Lasina I. Skapars R. Sivins A. (2016). Breath testing as potential colorectal cancer screening tool. Int. J. Cancer 138, 229–236. 10.1002/ijc.29701 26212114

[B10] Amann A. Costello B. D. L. Miekisch W. Schubert J. Buszewski B. Pleil J. (2014). The human volatilome: volatile organic compounds (VOCs) in exhaled breath, skin emanations, urine, feces and saliva. J. Breath. Res. 8, 034001. 10.1088/1752-7155/8/3/034001 24946087

[B11] American Cancer Society (2017). Stomach (gastric) cancer survival rates. American Cancer Society. Available at: https://www.cancer.org/cancer/types/stomach-cancer/detection-diagnosis-staging/survival-rates.html (Accessed July 3, 2023).

[B12] Antoniou S. X. Gaude E. Ruparel M. Van Der Schee M. P. Janes S. M. Rintoul R. C. (2019). The potential of breath analysis to improve outcome for patients with lung cancer. J. Breath. Res. 13, 034002. 10.1088/1752-7163/ab0bee 30822771

[B13] Aoki T. Nagaoka T. Kobayashi N. Kurahashi M. Tsuji C. Takiguchi H. (2017). Editor's highlight: prospective analyses of volatile organic compounds in obstructive sleep apnea patients. Toxicol. Sci. 156, 362–374. 10.1093/toxsci/kfw260 28003437

[B14] Asthma (2023). World health organization. Available at: https://www.who.int/news-room/fact-sheets/detail/asthma (Accessed June 28, 2023).

[B15] Asthma-Diagnosis (2023). National heart, lung, and blood Institute. Available at: https://www.nhlbi.nih.gov/health/asthma/diagnosis (Accessed July 25, 2023).

[B16] Balata H. Quaife S. L. Craig C. Ryan D. J. Bradley P. Crosbie P. A. J. (2022). Early diagnosis and lung cancer screening. Clin. Oncol. 34, 708–715. 10.1016/j.clon.2022.08.036 36175244

[B17] Barash O. Zhang W. Halpern J. M. Hua Q. L. Pan Y. Y. Kayal H. (2015). Differentiation between genetic mutations of breast cancer by breath volatolomics. Oncotarget 6, 44864–44876. 10.18632/oncotarget.6269 26540569 PMC4792597

[B18] Barba D. León-Sosa A. Lugo P. Suquillo D. Torres F. Surre F. (2021). Breast cancer, screening and diagnostic tools: all you need to know. Crit. Rev. Oncol. Hematol. 157, 103174. 10.1016/j.critrevonc.2020.103174 33249359

[B19] Basanta M. Ibrahim B. Dockry R. Douce D. Morris M. Singh D. (2012). Exhaled volatile organic compounds for phenotyping chronic obstructive pulmonary disease: a cross-sectional study. Respir. Res. 13, 72. 10.1186/1465-9921-13-72 22916684 PMC3514190

[B20] Basanta M. Koimtzis T. Singh D. Wilson I. Thomas C. L. P. (2007). An adaptive breath sampler for use with human subjects with an impaired respiratory function. Analyst 132, 153–163. 10.1039/b608608J 17260076

[B21] Bayrakli I. Öztürk Ö. Akman H. (2016). Investigation of acetone, butanol and carbon dioxide as new breath biomarkers for convenient and noninvasive diagnosis of obstructive sleep apnea syndrome. Biomed. Chromatogr. 30, 1890–1899. 10.1002/bmc.3757 27185417

[B22] Beale D. J. Jones O. A. H. Karpe A. V. Dayalan S. Oh D. Y. Kouremenos K. A. (2016). A review of analytical techniques and their application in disease diagnosis in breathomics and salivaomics research. Int. J. Mol. Sci. 18, 24. 10.3390/ijms18010024 28025547 PMC5297659

[B23] Beauchamp J. Herbig J. Gutmann R. Hansel A. (2008). On the use of Tedlar® bags for breath-gas sampling and analysis. J. Breath. Res. 2, 046001. 10.1088/1752-7155/2/4/046001 21386188

[B24] Beauchamp J. D. Miekisch W. (2020). “Breath sampling and standardization,” in Breathborne biomarkers and the human volatilome. Editors Beauchamp J. Davis C. Pleil J. (Amsterdam, Netherlands: Elsevier), 23–41. 10.1016/B978-0-12-819967-1.00002-5

[B25] Becker R. (2020). Non-invasive cancer detection using volatile biomarkers: is urine superior to breath? Med. Hypotheses 143, 110060. 10.1016/j.mehy.2020.110060 32683218

[B26] Behrendorff J. B. Y. H. (2021). Reductive cytochrome P450 reactions and their potential role in bioremediation. Front. Microbiol. 12, 649273. 10.3389/fmicb.2021.649273 33936006 PMC8081977

[B27] Berna A. Z. Akaho E. H. Harris R. M. Congdon M. Korn E. Neher S. (2021). Reproducible breath metabolite changes in children with SARS-CoV-2 infection. ACS Infect. Dis. 7, 2596–2603. 10.1021/acsinfecdis.1c00248 34319698 PMC8353987

[B28] Bhandari M. P. Polaka I. Vangravs R. Mezmale L. Veliks V. Kirshners A. (2023). Volatile markers for cancer in exhaled breath—could they be the signature of the gut microbiota? Molecules 28, 3488. 10.3390/molecules28083488 37110724 PMC10141340

[B29] Boyd N. F. Guo H. Martin L. J. Sun L. Stone J. Fishell E. (2007). Mammographic density and the risk and detection of breast cancer. N. Engl. J. Med. 356, 227–236. 10.1056/NEJMOA062790 17229950

[B30] Brinkman P. van de Pol M. A. Gerritsen M. G. Bos L. D. Dekker T. Smids B. S. (2017). Exhaled breath profiles in the monitoring of loss of control and clinical recovery in asthma. Clin. Exp. Allergy 47, 1159–1169. 10.1111/cea.12965 28626990

[B31] Bruderer T. Gaisl T. Gaugg M. T. Nowak N. Streckenbach B. Müller S. (2019). On-line analysis of exhaled breath focus review. Chem. Rev. 119, 10803–10828. 10.1021/acs.chemrev.9b00005 31594311

[B32] Buszewski B. Grzywinski D. Ligor T. Stacewicz T. Bielecki Z. Wojtas J. (2013). Detection of volatile organic compounds as biomarkers in breath analysis by different analytical techniques. Bioanalysis 5, 2287–2306. 10.4155/bio.13.183 24053244

[B33] Buszewski B. Kesy M. Ligor T. Amann A. (2007). Human exhaled air analytics: biomarkers of diseases. Biomed. Chromatogr. 21, 553–566. 10.1002/bmc.835 17431933

[B34] Buszewski B. Ligor T. Jezierski T. Wenda-Piesik A. Walczak M. Rudnicka J. (2012). Identification of volatile lung cancer markers by gas chromatography-mass spectrometry: comparison with discrimination by canines. Anal. Bioanal. Chem. 404, 141–146. 10.1007/s00216-012-6102-8 22660158 PMC3389235

[B35] Caldeira M. Perestrelo R. Barros A. S. Bilelo M. J. Morête A. Câmara J. S. (2012). Allergic asthma exhaled breath metabolome: a challenge for comprehensive two-dimensional gas chromatography. J. Chromatogr. A 1254, 87–97. 10.1016/j.chroma.2012.07.023 22835687

[B36] Calenic B. Miricescu D. Greabu M. Kuznetsov A. V. Troppmair J. Ruzsanyi V. (2015). Oxidative stress and volatile organic compounds: interplay in pulmonary, cardio-vascular, digestive tract systems and cancer. Open Chem. 13, 1020–1030. 10.1515/chem-2015-0105

[B37] Callol-Sanchez L. Munoz-Lucas M. A. Gomez-Martin O. Maldonado-Sanz J. A. Civera-Tejuca C. Gutierrez-Ortega C. (2017). Observation of nonanoic acid and aldehydes in exhaled breath of patients with lung cancer. J. Breath. Res. 11, 026004. 10.1088/1752-7163/aa6485 28440225

[B38] Cancer (2023). World health organization. Available at: https://www.who.int/news-room/fact-sheets/detail/cancer (Accessed May 17, 2023).

[B39] Castillo J. R. Peters S. P. Busse W. W. (2017). Asthma exacerbations: pathogenesis, prevention, and treatment. J. Allergy Clin. Immunol. Pract. 5, 918–927. 10.1016/j.jaip.2017.05.001 28689842 PMC5950727

[B40] Cazzola M. Segreti A. Capuano R. Bergamini A. Martinelli E. Calzetta L. (2015). Analysis of exhaled breath fingerprints and volatile organic compounds in COPD. COPD Res. Pract. 1, 7. 10.1186/s40749-015-0010-1

[B41] Cen Z. Lu B. Ji Y. Chen J. Liu Y. Jiang J. (2023). Virus-induced breath biomarkers: a new perspective to study the metabolic responses of COVID-19 vaccinees. Talanta 260, 124577. 10.1016/j.talanta.2023.124577 37116359 PMC10122548

[B42] Chen S. Mahadevan V. Zieve L. (1970). Volatile fatty acids in the breath of patients with cirrhosis of the liver. J. Lab. Clin. Med. 75, 622–627. 10.5555/uri:pii:0022214370901605 5444347

[B43] Chen X. Muhammad K. G. Madeeha C. Fu W. Xu L. Hu Y. (2021). Calculated indices of volatile organic compounds (VOCs) in exhalation for lung cancer screening and early detection. Lung Cancer 154, 197–205. 10.1016/j.lungcan.2021.02.006 33653598

[B44] Colorectal Cancer—Cancer Stat Facts (2023). National cancer Institute. Available at: https://seer.cancer.gov/statfacts/html/colorect.html (Accessed May 23, 2023).

[B45] Conti C. B. Agnesi S. Scaravaglio M. Masseria P. Dinelli M. E. Oldani M. (2023). Early gastric cancer: update on prevention, diagnosis and treatment. Int. J. Environ. Res. Public Health 20, 2149. 10.3390/ijerph20032149 36767516 PMC9916026

[B46] Conway J. R. Lex A. Gehlenborg N. (2017). UpSetR: an R package for the visualization of intersecting sets and their properties. Bioinformatics 33, 2938–2940. 10.1093/bioinformatics/btx364 28645171 PMC5870712

[B47] Cope K. A. Watson M. T. Foster W. M. Sehnert S. S. Risby T. H. (2004). Effects of ventilation on the collection of exhaled breath in humans. J. Appl. Physiol. (1985) 96, 1371–1379. 10.1152/japplphysiol.01034.2003 14672964

[B48] Corradi M. Poli D. Banda I. Bonini S. Mozzoni P. Pinelli S. (2015). Exhaled breath analysis in suspected cases of non-small-cell lung cancer: a cross-sectional study. J. Breath. Res. 9, 027101. 10.1088/1752-7155/9/2/027101 25634546

[B49] Cystic Fibrosis-Causes (2023). National heart, lung, and blood Institute. Available at: https://www.nhlbi.nih.gov/health/cystic-fibrosis/causes (Accessed June 6, 2023).

[B50] Davidson L. S. P. (1949). Mercaptan in the breath of patients with severe liver disease. Lancet 2, 197. 10.1016/S0140-6736(49)91197-6 18136275

[B51] De Lacy Costello B. Amann A. Al-Kateb H. Flynn C. Filipiak W. Khalid T. (2014). A review of the volatiles from the healthy human body. J. Breath. Res. 8, 014001. 10.1088/1752-7155/8/1/014001 24421258

[B52] De Vos W. M. Tilg H. Van Hul M. Cani P. D. (2022). Gut microbiome and health: mechanistic insights. Gut 71, 1020–1032. 10.1136/gutjnl-2021-326789 35105664 PMC8995832

[B53] Drabińska N. Flynn C. Ratcliffe N. Belluomo I. Myridakis A. Gould O. (2021). A literature survey of all volatiles from healthy human breath and bodily fluids: the human volatilome. J. Breath. Res. 15, 034001. 10.1088/1752-7163/abf1d0 33761469

[B54] Dudzik D. Barbas-Bernardos C. García A. Barbas C. (2018). Quality assurance procedures for mass spectrometry untargeted metabolomics. a review. J. Pharm. Biomed. Anal. 147, 149–173. 10.1016/J.JPBA.2017.07.044 28823764

[B55] Eriksen S. P. Kulkarni A. B. (1963). Methanol in normal human breath. Science 141, 639–640. 10.1126/science.141.3581.639 17781761

[B56] Fazleen A. Wilkinson T. (2020). Early COPD: current evidence for diagnosis and management. Ther. Adv. Respir. Dis. 14, 1753466620942128. 10.1177/1753466620942128 32664818 PMC7394029

[B57] Female Breast Cancer (2023). Cancer Stat facts. National cancer Institute. Available at: https://seer.cancer.gov/statfacts/html/breast.html (Accessed May 26, 2023).

[B58] Feng F. Tian Y. Xu G. Liu Z. Liu S. Zheng G. (2017). Diagnostic and prognostic value of CEA, CA19-9, AFP and CA125 for early gastric cancer. BMC Cancer 17, 737. 10.1186/s12885-017-3738-y 29121872 PMC5679342

[B59] Ferlay J. Colombet M. Soerjomataram I. Parkin D. M. Piñeros M. Znaor A. (2021). Cancer statistics for the year 2020: an overview. Int. J. Cancer 149, 778–789. 10.1002/ijc.33588 33818764

[B60] Fernando S. M. Tran A. Cheng W. Klompas M. Kyeremanteng K. Mehta S. (2020). Diagnosis of ventilator-associated pneumonia in critically ill adult patients-A systematic review and meta-analysis. Intensive Care Med. 46, 1170–1179. 10.1007/s00134-020-06036-z 32306086 PMC7223448

[B61] Ferreira-Coimbra J. Sarda C. Rello J. (2020). Burden of community-acquired pneumonia and unmet clinical needs. Adv. Ther. 37, 1302–1318. 10.1007/s12325-020-01248-7 32072494 PMC7140754

[B62] Filipiak W. Beer R. Sponring A. Filipiak A. Ager C. Schiefecker A. (2015). Breath analysis for *in vivo* detection of pathogens related to ventilator-associated pneumonia in intensive care patients: a prospective pilot study. J. Breath. Res. 9, 016004. 10.1088/1752-7155/9/1/016004 25557917

[B63] Filipiak W. Sponring A. Filipiak A. Baur M. Ager C. Wiesenhofer H. (2013). “Volatile organic compounds (VOCs) released by pathogenic microorganisms *in vitro*: potential breath biomarkers for early-stage diagnosis of disease,” in Volatile biomarkers. Editors Amann A. Smith D. (Amsterdam, Netherlands: Elsevier), 463–512. 10.1016/B978-0-44-462613-4.00023-4

[B64] Fortes P. R. Petruci J. F. S. Raimundo I. M. Jr. (2017). Optical gas sensors for exhaled breath analysis. SPIE Press. 10.1117/3.2284712

[B65] Fowler S. J. Basanta-Sanchez M. Xu Y. Goodacre R. Dark P. M. (2015). Surveillance for lower airway pathogens in mechanically ventilated patients by metabolomic analysis of exhaled breath: a case-control study. Thorax 70, 320–325. 10.1136/thoraxjnl-2014-206273 25661115

[B66] Furge L. L. Guengerich F. P. (2006). Cytochrome P450 enzymes in drug metabolism and chemical toxicology: an introduction. Biochem. Mol. Biol. Educ. 34, 66–74. 10.1002/bmb.2006.49403402066 21638641

[B67] Gahleitner F. Guallar-Hoyas C. Beardsmore C. S. Pandya H. C. Thomas C. P. (2013). Metabolomics pilot study to identify volatile organic compound markers of childhood asthma in exhaled breath. Bioanalysis 5, 2239–2247. 10.4155/bio.13.184 24053239

[B68] Gaida A. Holz O. Nell C. Schuchardt S. Lavae-Mokhtari B. Kruse L. (2016). A dual center study to compare breath volatile organic compounds from smokers and non-smokers with and without COPD. J. Breath. Res. 10, 026006. 10.1088/1752-7155/10/2/026006 27082437

[B69] Gao J. Zou Y. Wang Y. Wang F. Lang L. Wang P. (2016). Breath analysis for noninvasively differentiating Acinetobacter baumannii ventilator-Associated pneumonia from its respiratory tract colonization of ventilated patients. J. Breath. Res. 10, 027102. 10.1088/1752-7155/10/2/027102 27272697

[B70] Ghosh C. Singh V. Grandy J. Pawliszyn J. (2020). Recent advances in breath analysis to track human health by new enrichment technologies. J. Sep. Sci. 43, 226–240. 10.1002/jssc.201900769 31826324

[B71] GOLDCOPD (2023). Global initiative for chronic obstructive lung disease. Available at: www.goldcopd.org.

[B72] González-Domínguez R. Jáuregui O. Queipo-Ortuño M. I. Andrés-Lacueva C. (2020). Characterization of the human exposome by a comprehensive and quantitative large-scale multianalyte metabolomics platform. Anal. Chem. 92, 13767–13775. 10.1021/acs.analchem.0c02008 32966057

[B73] Goss C. H. (2019). Acute pulmonary exacerbations in cystic fibrosis. Semin. Respir. Crit. Care Med. 40, 792–803. 10.1055/s-0039-1697975 31659730 PMC7528649

[B74] Grote C. Pawliszyn J. (1997). Solid-phase microextraction for the analysis of human breath. Anal. Chem. 69, 587–596. 10.1021/ac960749l 9043197

[B75] Haick H. Broza Y. Y. Mochalski P. Ruzsanyi V. Amann A. (2014). Assessment, origin, and implementation of breath volatile cancer markers. Chem. Soc. Rev. 43, 1423–1449. 10.1039/c3cs60329f 24305596 PMC4909138

[B76] Hakim M. Broza Y. Y. Barash O. Peled N. Phillips M. Amann A. (2012). Volatile organic compounds of lung cancer and possible biochemical pathways. Chem. Rev. 112, 5949–5966. 10.1021/cr300174a 22991938

[B77] Hamashima C. (2016). Benefits and harms of endoscopic screening for gastric cancer. World J. Gastroenterol. 22, 6385–6392. 10.3748/wjg.v22.i28.6385 27605874 PMC4968120

[B78] Hanna G. B. Boshier P. R. Markar S. R. Romano A. (2019). Accuracy and methodologic challenges of volatile organic compound–based exhaled breath tests for cancer diagnosis: a systematic review and meta-analysis. JAMA Oncol. 5, e182815. 10.1001/jamaoncol.2018.2815 30128487 PMC6439770

[B79] Helsingen L. M. Kalager M. (2022). Colorectal cancer screening — approach, evidence, and future directions. NEJM Evid. 1. 10.1056/EVIDra2100035 38319175

[B80] Herrera-Pariente C. Montori S. Llach J. Bofill A. Albeniz E. Moreira L. (2021). Biomarkers for gastric cancer screening and early diagnosis. Biomedicines 9, 1448. 10.3390/biomedicines9101448 34680565 PMC8533304

[B81] Hunt R. Williams D. (1977). Spectrometric measurement of ammonia in normal human breath. Int. Lab. 11.

[B82] Ibrahim W. Cordell R. L. Wilde M. J. Richardson M. Carr L. Dasi A. S. D. (2021). Diagnosis of COVID-19 by exhaled breath analysis using gas chromatography–mass spectrometry. ERJ Open Res. 7, 00139–02021. 10.1183/23120541.00139-2021 34235208 PMC8255539

[B83] Janssens E. van Meerbeeck J. P. Lamote K. (2020). Volatile organic compounds in human matrices as lung cancer biomarkers: a systematic review. Crit. Rev. Oncol. Hematol. 153, 103037. 10.1016/j.critrevonc.2020.103037 32771940

[B84] Jareño-Esteban J. J. Muñoz-Lucas M. Á. Gómez-Martín Ó. Utrilla-Trigo S. Gutiérrez-Ortega C. Aguilar-Ros A. (2017). Study of 5 volatile organic compounds in exhaled breath in chronic obstructive pulmonary disease. Arch. Bronconeumol 53, 251–256. 10.1016/j.arbres.2016.09.003 27780574

[B85] Kapur V. K. Auckley D. H. Chowdhuri S. Kuhlmann D. C. Mehra R. Ramar K. (2017). Clinical practice guideline for diagnostic testing for adult obstructive sleep apnea: an american academy of sleep medicine clinical practice guideline. J. Clin. Sleep. Med. 13, 479–504. 10.5664/jcsm.6506 28162150 PMC5337595

[B86] Karamanou M. Androutsos G. (2013). Antoine-Laurent de Lavoisier (1743-1794) and the birth of respiratory physiology. Thorax 68, 978–979. 10.1136/thoraxjnl-2013-203840 23723342

[B87] Khoubnasabjafari M. Mogaddam M. R. A. Rahimpour E. Soleymani J. Saei A. A. Jouyban A. (2022). Breathomics: review of sample collection and analysis, data modeling and clinical applications. Crit. Rev. Anal. Chem. 52, 1461–1487. 10.1080/10408347.2021.1889961 33691552

[B88] Koureas M. Kalompatsios D. Amoutzias G. D. Hadjichristodoulou C. Gourgoulianis K. Tsakalof A. (2021). Comparison of targeted and untargeted approaches in breath analysis for the discrimination of lung cancer from benign pulmonary diseases and healthy persons. Molecules 26, 2609. 10.3390/molecules26092609 33946997 PMC8125376

[B89] Koureas M. Kirgou P. Amoutzias G. Hadjichristodoulou C. Gourgoulianis K. Tsakalof A. (2020). Target analysis of volatile organic compounds in exhaled breath for lung cancer discrimination from other pulmonary diseases and healthy persons. Metabolites 10, 317. 10.3390/metabo10080317 32756521 PMC7464039

[B90] Kucirka L. M. Lauer S. A. Laeyendecker O. Boon D. Lessler J. (2020). Variation in false-negative rate of reverse transcriptase polymerase chain reaction–based SARS-CoV-2 tests by time since exposure. Ann. Intern Med. 173, 262–267. 10.7326/M20-1495 32422057 PMC7240870

[B91] Kuo T. C. Tan C. E. Wang S. Y. Lin O. A. Su B. H. Hsu M. T. (2020). Human breathomics database. Database (Oxford) 2020, baz139. 10.1093/database/baz139 31976536 PMC6978997

[B92] Kuruvilla M. E. Lee F. E. H. Lee G. B. (2019). Understanding asthma phenotypes, endotypes, and mechanisms of disease. Clin. Rev. Allergy Immunol. 56, 219–233. 10.1007/s12016-018-8712-1 30206782 PMC6411459

[B93] Lawal O. Ahmed W. M. Nijsen T. M. E. Goodacre R. Fowler S. J. (2017). Exhaled breath analysis: a review of “breath-taking” methods for off-line analysis. Metabolomics 13, 110. 10.1007/S11306-017-1241-8 28867989 PMC5563344

[B94] Lévy P. Kohler M. McNicholas W. T. Barbé F. McEvoy R. D. Somers V. K. (2015). Obstructive sleep apnoea syndrome. Nat. Rev. Dis. Prim. 1, 15015. 10.1038/nrdp.2015.15 27188535

[B95] Li J. Peng Y. Liu Y. Li W. Jin Y. Tang Z. (2014). Investigation of potential breath biomarkers for the early diagnosis of breast cancer using gas chromatography-mass spectrometry. Clin. Chim. Acta 436, 59–67. 10.1016/j.cca.2014.04.030 24815034

[B96] Li W. Dai W. Liu M. Long Y. Wang C. Xie S. (2019). VOC biomarkers identification and predictive model construction for lung cancer based on exhaled breath analysis: research protocol for an exploratory study. BMJ Open 9, e028448. 10.1136/BMJOPEN-2018-028448 PMC6701581 31399453

[B97] Ligor T. Pater Ł. Buszewski B. (2015). Application of an artificial neural network model for selection of potential lung cancer biomarkers. J. Breath. Res. 9, 027106. 10.1088/1752-7155/9/2/027106 25944812

[B98] Løberg M. Lousdal M. L. Bretthauer M. Kalager M. (2015). Benefits and harms of mammography screening. Breast Cancer Res. 17, 63. 10.1186/S13058-015-0525-Z 25928287 PMC4415291

[B99] Lung Cancer Survival Rates (2023). 5-Year survival rates for lung cancer. American Cancer Society. Available at: https://www.cancer.org/cancer/types/lung-cancer/detection-diagnosis-staging/survival-rates.html (Accessed June 22, 2023).

[B100] Lv R. Liu X. Zhang Y. Dong N. Wang X. He Y. (2023). Pathophysiological mechanisms and therapeutic approaches in obstructive sleep apnea syndrome. Signal Transduct. Target Ther. 8, 218. 10.1038/s41392-023-01496-3 37230968 PMC10211313

[B101] Mansurova M. Ebert B. E. Blank L. M. Ibáñez A. J. (2018). A breath of information: the volatilome. Curr. Genet. 64, 959–964. 10.1007/s00294-017-0800-x 29279954

[B102] Markar S. R. Wiggins T. Kumar S. Hanna G. B. (2015). Exhaled breath analysis for the diagnosis and assessment of endoluminal gastrointestinal diseases. J. Clin. Gastroenterol. 49, 1–8. 10.1097/MCG.0000000000000247 25319742

[B103] McKee H. C. Rhoades J. W. Campbell J. Gross A. L. (1962). Acetonitrile in body fluids related to smoking. Public Health Rep. 77, 553–554. 10.2307/4591551 19316418 PMC1914971

[B104] Meyer N. Dallinga J. W. Nuss S. J. Moonen E. J. C. van Berkel J. J. B. N. Akdis C. (2014). Defining adult asthma endotypes by clinical features and patterns of volatile organic compounds in exhaled air. Respir. Res. 15, 136. 10.1186/S12931-014-0136-8 25431084 PMC4264530

[B105] Miekisch W. Herbig J. Schubert J. K. (2012). Data interpretation in breath biomarker research: pitfalls and directions. J. Breath. Res. 6, 036007. 10.1088/1752-7155/6/3/036007 22854185

[B106] Miekisch W. Kischkel S. Sawacki A. Liebau T. Mieth M. Schubert J. K. (2008). Impact of sampling procedures on the results of breath analysis. J. Breath. Res. 2, 026007. 10.1088/1752-7155/2/2/026007 21383448

[B107] Mochalski P. Wzorek B. Śliwka I. Amann A. (2009). Suitability of different polymer bags for storage of volatile sulphur compounds relevant to breath analysis. J. Chromatogr. B Anal. Technol. Biomed. Life Sci. 877, 189–196. 10.1016/j.jchromb.2008.12.003 19109077

[B108] Modi A. R. Kovacs C. S. (2020). Hospital-acquired and ventilator-associated pneumonia: diagnosis, management, and prevention. Cleve Clin. J. Med. 87, 633–639. 10.3949/ccjm.87a.19117 33004324

[B109] Monedeiro F. Monedeiro-Milanowski M. Ratiu I. A. Brożek B. Ligor T. Buszewski B. (2021). Needle trap device-GC-MS for characterization of lung diseases based on breath VOC profiles. Molecules 26, 1789. 10.3390/molecules26061789 33810121 PMC8004837

[B110] Morales J. S. Valenzuela P. L. Castillo-García A. Butragueño J. Jiménez-Pavón D. Carrera-Bastos P. (2022). The exposome and immune health in times of the covid-19 pandemic. Nutrients 14, 24. 10.3390/nu14010024 PMC8746533 35010900

[B111] Muñoz-Lucas M. Á. Jareño-Esteban J. Gutiérrez-Ortega C. López-Guijarro P. Collado-Yurrita L. Quintana-Díaz M. (2020). Influence of chronic obstructive pulmonary disease on volatile organic compounds in patients with non-small cell lung cancer. Arch. Bronconeumol 56, 801–805. 10.1016/j.arbr.2020.10.004 35373775

[B112] Munro S. C. Baker Di. Giuliano K. K. Sullivan S. C. Haber J. Jones B. E. (2021). Nonventilator hospital-acquired pneumonia: a call to action. Infect. Control Hosp. Epidemiol. 42, 991–996. 10.1017/ice.2021.239 34103108 PMC10947501

[B113] Murray R. Rodwell V. Bender D. Botham K. M. Weil P. Anthony (2009). Harper’s illustrated biochemistry. 28th ed New York: Citiseer.

[B114] Myers R. Ruszkiewicz D. M. Meister A. Bartolomeu C. Atkar-Khattra S. Thomas C. L. P. (2023). Breath testing for SARS-CoV-2 infection. EBioMedicine 92, 104584. 10.1016/j.ebiom.2023.104584 37121096 PMC10140675

[B115] Nardi-Agmon I. Abud-Hawa M. Liran O. Gai-Mor N. Ilouze M. Onn A. (2016). Exhaled breath analysis for monitoring response to treatment in advanced lung cancer. J. Thorac. Oncol. 11, 827–837. 10.1016/j.jtho.2016.02.017 26968885

[B116] Netzker T. Shepherdson E. M. F. Zambri M. P. Elliot M. A. (2020). Bacterial volatile compounds: functions in communication, cooperation, and competition. Annu. Rev. Microbiol. 74, 409–430. 10.1146/annurev-micro-011320-015542 32667838

[B117] Neveu V. Nicolas G. Amara A. Salek R. M. Scalbert A. (2023). The human microbial exposome: expanding the Exposome-Explorer database with gut microbial metabolites. Sci. Rep. 13, 1946. 10.1038/s41598-022-26366-w 36732606 PMC9894932

[B118] Niedermaier T. Tikk K. Gies A. Bieck S. Brenner H. (2020). Sensitivity of fecal immunochemical test for colorectal cancer detection differs according to stage and location. Clin. Gastroenterol. Hepatol. 18, 2920–2928. 10.1016/j.cgh.2020.01.025 31988043

[B119] Nooreldeen R. Bach H. (2021). Current and future development in lung cancer diagnosis. Int. J. Mol. Sci. 22, 8661. 10.3390/ijms22168661 34445366 PMC8395394

[B120] Nowak N. Engler A. Thiel S. Stöberl A. S. Sinues P. Zenobi R. (2021). Validation of breath biomarkers for obstructive sleep apnea. Sleep. Med. 85, 75–86. 10.1016/j.sleep.2021.06.040 34280868

[B121] Papaefstathiou E. Stylianou M. Andreou C. Agapiou A. (2020). Breath analysis of smokers, non-smokers, and e-cigarette users. J. Chromatogr. B 1160, 122349. 10.1016/j.jchromb.2020.122349 32920481

[B122] Patti G. J. Yanes O. Siuzdak G. (2012). Innovation: metabolomics: the apogee of the omics trilogy. Nat. Rev. Mol. Cell Biol. 13, 263–269. 10.1038/NRM3314 22436749 PMC3682684

[B123] Pauling L. Robinson A. B. Teranishit R. Cary P. (1971). Quantitative analysis of urine vapor and breath by gas-liquid partition chromatography. Proc. Natl. Acad. Sci. U. S. A. 68, 2374–2376. 10.1073/pnas.68.10.2374 5289873 PMC389425

[B124] Pennazza G. Santonico M. Incalzi R. A. Scarlata S. Chiurco D. Vernile C. (2014). Measure chain for exhaled breath collection and analysis: a novel approach suitable for frail respiratory patients. Sens. Actuators B Chem. 204, 578–587. 10.1016/j.snb.2014.08.007

[B125] Pesesse R. Stefanuto P. H. Schleich F. Louis R. Focant J. F. (2019). Multimodal chemometric approach for the analysis of human exhaled breath in lung cancer patients by TD-GC × GC-TOFMS. J. Chromatogr. B 1114–1115, 146–153. 10.1016/j.jchromb.2019.01.029 30745111

[B126] Phillips C. O. Syed Y. Parthaláin N. Zwiggelaar R. M. Claypole T. C. Lewis K. E. (2012). Machine learning methods on exhaled volatile organic compounds for distinguishing COPD patients from healthy controls. J. Breath. Res. 6, 036003. 10.1088/1752-7155/6/3/036003 22759349

[B127] Phillips M. (1992). Breath tests in medicine. Sci. Am. 267, 74–79. 10.1038/scientificamerican0792-74 1502511

[B128] Phillips M. (1997). Method for the collection and assay of volatile organic compounds in breath. Anal. Biochem. 247, 272–278. 10.1006/abio.1997.2069 9177688

[B129] Pizzini A. Filipiak W. Wille J. Ager C. Wiesenhofer H. Kubinec R. (2018). Analysis of volatile organic compounds in the breath of patients with stable or acute exacerbation of chronic obstructive pulmonary disease. J. Breath. Res. 12, 036002. 10.1088/1752-7163/aaa4c5 29295966

[B130] Pleil J. D. Stiegel M. A. Risby T. H. (2013). Clinical breath analysis: discriminating between human endogenous compounds and exogenous (environmental) chemical confounders. J. Breath. Res. 7, 017107. 10.1088/1752-7155/7/1/017107 23445880

[B131] Qaseem A. Crandall C. J. Mustafa R. A. Hicks L. A. Wilt T. J. Clinical Guidelines Committee of the American College of Physicians (2019). Screening for colorectal cancer in asymptomatic average-risk adults: a guidance statement from the American College of Physicians. Ann. Intern Med. 171, 643–654. 10.7326/M19-0642 31683290 PMC8152103

[B132] Ratcliffe N. Wieczorek T. Drabińska N. Drabińska N. Gould O. Osborne A. (2020). A mechanistic study and review of volatile products from peroxidation of unsaturated fatty acids: an aid to understanding the origins of volatile organic compounds from the human body. J. Breath. Res. 14, 034001. 10.1088/1752-7163/ab7f9d 32163929

[B133] Ratiu I. A. Ligor T. Bocos-Bintintan V. Mayhew C. A. Buszewski B. (2020). Volatile organic compounds in exhaled breath as fingerprints of lung cancer, asthma and COPD. J. Clin. Med. 10, 32. 10.3390/JCM10010032 33374433 PMC7796324

[B134] Rey-Stolle F. Dudzik D. Gonzalez-Riano C. Fernández-García M. Alonso-Herranz V. Rojo D. (2021). Low and high resolution gas chromatography-mass spectrometry for untargeted metabolomics: a tutorial. Anal. Chim. Acta 1210, 339043. 10.1016/j.aca.2021.339043 35595356

[B135] Robroeks C. M. Van Berkel J. J. Jöbsis Q. Van Schooten F. J. Dallinga J. W. Wouters E. F. (2013). Exhaled volatile organic compounds predict exacerbations of childhood asthma in a 1-year prospective study. Eur. Respir. J. 42, 98–106. 10.1183/09031936.00010712 23645402

[B136] Rodak O. Peris-Díaz M. D. Olbromski M. Podhorska-Okołów M. Dzięgiel P. (2021). Current landscape of non-small cell lung cancer: epidemiology, histological classification, targeted therapies, and immunotherapy. Cancers (Basel) 13, 4705. 10.3390/cancers13184705 34572931 PMC8470525

[B137] Roesch E. A. Nichols D. P. Chmiel J. F. (2018). Inflammation in cystic fibrosis: an update. Pediatr. Pulmonol. 53, S30–S50. 10.1002/ppul.24129 29999593

[B138] Rudnicka J. Kowalkowski T. Buszewski B. (2019). Searching for selected VOCs in human breath samples as potential markers of lung cancer. Lung Cancer 135, 123–129. 10.1016/j.lungcan.2019.02.012 31446984

[B139] Rudnicka J. Walczak M. Kowalkowski T. Jezierski T. Buszewski B. (2014). Determination of volatile organic compounds as potential markers of lung cancer by gas chromatography-mass spectrometry versus trained dogs. Sens. Actuators B Chem. 202, 615–621. 10.1016/j.snb.2014.06.006

[B140] Saasa V. Malwela T. Beukes M. Mokgotho M. Liu C. P. Mwakikunga B. (2018). Sensing technologies for detection of acetone in human breath for diabetes diagnosis and monitoring. Diagn. (Basel) 8, 12. 10.3390/diagnostics8010012 PMC5871995 29385067

[B141] Saidi T. Moufid M. de Jesus Beleño-Saenz K. Welearegay T. G. El Bari N. Lisset Jaimes-Mogollon A. (2020). Non-invasive prediction of lung cancer histological types through exhaled breath analysis by UV-irradiated electronic nose and GC/QTOF/MS. Sens. Actuators B Chem. 311, 127932. 10.1016/j.snb.2020.127932

[B142] Sakumura Y. Koyama Y. Tokutake H. Hida T. Sato K. Itoh T. (2017). Diagnosis by volatile organic compounds in exhaled breath from lung cancer patients using support vector machine algorithm. Sensors (Basel) 17, 287. 10.3390/s17020287 28165388 PMC5335963

[B143] Schallschmidt K. Becker R. Jung C. Bremser W. Walles T. Neudecker J. (2016). Comparison of volatile organic compounds from lung cancer patients and healthy controls - challenges and limitations of an observational study. J. Breath. Res. 10, 046007. 10.1088/1752-7155/10/4/046007 27732569

[B144] Schleich F. N. Zanella D. Stefanuto P. H. Bessonov K. Smolinska A. Dallinga J. W. (2019). Exhaled volatile organic compounds are able to discriminate between neutrophilic and eosinophilic asthma. Am. J. Respir. Crit. Care Med. 200, 444–453. 10.1164/rccm.201811-2210OC 30973757

[B145] Schnabel R. Fijten R. Smolinska A. Dallinga J. Boumans M. L. Stobberingh E. (2015). Analysis of volatile organic compounds in exhaled breath to diagnose ventilator-associated pneumonia. Sci. Rep. 5, 17179. 10.1038/srep17179 26608483 PMC4660425

[B146] Schulz E. Woollam M. Grocki P. Davis M. D. Agarwal M. (2023). Methods to detect volatile organic compounds for breath biopsy using solid-phase microextraction and gas chromatography–mass spectrometry. Molecules 28, 4533. 10.3390/molecules28114533 37299010 PMC10254745

[B147] Schwarz E. I. Engler A. Kohler M. (2017). Exhaled breath analysis in obstructive sleep apnea. Expert Rev. Respir. Med. 11, 631–639. 10.1080/17476348.2017.1338950 28583012

[B148] Scohy A. Anantharajah A. Bodéus M. Kabamba-Mukadi B. Verroken A. Rodriguez-Villalobos H. (2020). Low performance of rapid antigen detection test as frontline testing for COVID-19 diagnosis. J. Clin. Virol. 129, 104455. 10.1016/j.jcv.2020.104455 32485618 PMC7240272

[B149] Sethi S. Nanda R. Chakraborty T. (2013). Clinical application of volatile organic compound analysis for detecting infectious diseases. Clin. Microbiol. Rev. 26, 462–475. 10.1128/CMR.00020-13 23824368 PMC3719490

[B150] Sharma A. Kumar R. Varadwaj P. (2023). Smelling the disease: diagnostic potential of breath analysis. Mol. Diagn Ther. 27, 321–347. 10.1007/s40291-023-00640-7 36729362 PMC9893210

[B151] Sidorova D. E. Plyuta V. A. Padiy D. A. Kupriyanova E. V. Roshina N. V. Koksharova O. A. (2021). The effect of volatile organic compounds on different organisms: agrobacteria, plants and insects. Microorganisms 10, 69. 10.3390/microorganisms10010069 35056518 PMC8781025

[B152] Simenhoff M. L. Burke J. F. Saukkonen J. J. Ordinario A. T. Doty R. Dunn S. (1977). Biochemical profile or uremic breath. N. Engl. J. Med. 297, 132–135. 10.1056/NEJM197707212970303 865584

[B153] Simrén M. Stotzer P. O. (2006). Use and abuse of hydrogen breath tests. Gut 55, 297–303. 10.1136/gut.2005.075127 16474100 PMC1856094

[B154] Smolinska A. Klaassen E. M. M. Dallinga J. W. Van De Kant K. D. G. Jobsis Q. Moonen E. J. C. (2014). Profiling of volatile organic compounds in exhaled breath as a strategy to find early predictive signatures of asthma in children. PLoS One 9, e95668. 10.1371/journal.pone.0095668 24752575 PMC3994075

[B155] Sola-Martínez R. A. Lozano-Terol G. Gallego-Jara J. Cánovas Díaz M. de Diego Puente T. (2022). “Offline breath analysis: standardization of breath sampling and analysis using mass spectrometry and innovative algorithms,” in Breath analysis. Bioanalytical reviews. Editor Weigl S. (Cham: Springer), 19–44. 10.1007/11663_2022_21

[B156] Sola-Martínez R. A. Lozano-Terol G. Gallego-Jara J. Morales E. Cantero-Cano E. Sánchez-Solís de Querol M. (2021). Exhaled volatilome analysis as a useful tool to discriminate asthma with other coexisting atopic diseases in women of childbearing age. Sci. Rep. 11, 13823. 10.1038/S41598-021-92933-2 34226570 PMC8257728

[B157] Stöberl A. S. Schwarz E. I. Haile S. R. Turnbull C. D. Rossi V. A. Stradling J. R. (2017). Night-to-night variability of obstructive sleep apnea. J. Sleep. Res. 26, 782–788. 10.1111/jsr.12558 28548301

[B158] Sumner L. W. Amberg A. Barrett D. Beale M. H. Beger R. Daykin C. A. (2007). Proposed minimum reporting standards for chemical analysis chemical analysis working group (CAWG) metabolomics standards initiative (MSI). Metabolomics 3, 211–221. 10.1007/S11306-007-0082-2 24039616 PMC3772505

[B159] Tang Z. Liu Y. Duan Y. (2015). Breath analysis: technical developments and challenges in the monitoring of human exposure to volatile organic compounds. J. Chromatogr. B Anal. Technol. Biomed. Life Sci. 1002, 285–299. 10.1016/j.jchromb.2015.08.041 26343020

[B160] The Global Asthma Report (2022). The global asthma Report 2022. Int. J. Tuberc. Lung Dis. 26, S1–S102. 10.5588/ijtld.22.1010 36303302

[B161] Tong H. Wang Y. Li Y. Liu S. Chi C. Liu D. (2017). Volatile organic metabolites identify patients with gastric carcinoma, gastric ulcer, or gastritis and control patients. Cancer Cell Int. 17, 108. 10.1186/s12935-017-0475-x 29200968 PMC5699190

[B162] Trefz P. Kischkel S. Hein D. James E. S. Schubert J. K. Miekisch W. (2012). Needle trap micro-extraction for VOC analysis: effects of packing materials and desorption parameters. J. Chromatogr. A 1219, 29–38. 10.1016/j.chroma.2011.10.077 22137782

[B163] Trujillo-Rodríguez M. J. Pacheco-Fernández I. Taima-Mancera I. Díaz J. H. A. Pino V. (2020). Evolution and current advances in sorbent-based microextraction configurations. J. Chromatogr. A 1634, 461670. 10.1016/j.chroma.2020.461670 33197845

[B164] Van Der Schee M. P. Paff T. Brinkman P. Van Aalderen W. M. C. Haarman E. G. Sterk P. J. (2015). Breathomics in lung disease. Chest 147, 224–231. 10.1378/chest.14-0781 25560860

[B165] van Horck M. Smolinska A. Wesseling G. de Winter-De Groot K. de Vreede I. Winkens B. (2021). Exhaled volatile organic compounds detect pulmonary exacerbations early in children with cystic fibrosis: results of a 1 year observational pilot study. J. Breath. Res. 15, 026012. 10.1088/1752-7163/abda55 33630756

[B166] Van Oort P. M. P. De Bruin S. Hans W. Knobel H. H. Schultz M. J. Bos L. D. (2017a). Exhaled breath metabolomics for the diagnosis of pneumonia in intubated and mechanically-ventilated intensive care unit (ICU)-patients. Int. J. Mol. Sci. 18, 449. 10.3390/ijms18020449 28218729 PMC5343983

[B167] van Oort P. M. P. Nijsen T. Weda H. Knobel H. Dark P. Felton T. (2017b). BreathDx - molecular analysis of exhaled breath as a diagnostic test for ventilator-associated pneumonia: protocol for a European multicentre observational study. BMC Pulm. Med. 17, 1. 10.1186/s12890-016-0353-7 28049457 PMC5210294

[B168] Van Oort P. M. P. Nijsen T. M. White I. R. Knobel H. H. Felton T. Rattray N. (2022). Untargeted molecular analysis of exhaled breath as a diagnostic test for ventilator-associated lower respiratory tract infections (BreathDx). Thorax 77, 79–81. 10.1136/thoraxjnl-2021-217362 34088787 PMC8685633

[B169] van Velzen P. Brinkman P. Knobel H. H. van den Berg J. W. K. Jonkers R. E. Loijmans R. J. (2019). Exhaled breath profiles before, during and after exacerbation of COPD: a prospective follow-up study. COPD 16, 330–337. 10.1080/15412555.2019.1669550 31588813

[B170] Van Vliet D. Smolinska A. Jöbsis Q. Rosias P. Muris J. Dallinga J. (2017). Can exhaled volatile organic compounds predict asthma exacerbations in children? J. Breath. Res. 11, 016016. 10.1088/1752-7163/aa5a8b 28102830

[B171] Van Vliet D. Smolinska A. Jöbsis Q. Rosias P. P. R. Muris J. W. M. Dallinga J. W. (2016). Association between exhaled inflammatory markers and asthma control in children. J. Breath. Res. 10, 016014. 10.1088/1752-7155/10/1/016014 26893372

[B172] Vas G. Vékey K. (2004). Solid-phase microextraction: a powerful sample preparation tool prior to mass spectrometric analysis. J. Mass Spectrom. 39, 233–254. 10.1002/jms.606 15039931

[B173] Vaz A. D. N. Coon M. J. (1987). Hydrocarbon formation in the reductive cleavage of hydroperoxides by cytochrome P-450. Proc. Natl. Acad. Sci. U.S.A. 84, 1172–1176. 10.1073/pnas.84.5.1172 3103131 PMC304388

[B174] Veith A. Moorthy B. (2018). Role of cytochrome P450s in the generation and metabolism of reactive oxygen species. Curr. Opin. Toxicol. 7, 44–51. 10.1016/j.cotox.2017.10.003 29527583 PMC5841237

[B175] Vineis P. Robinson O. Chadeau-Hyam M. Dehghan A. Mudway I. Dagnino S. (2020). What is new in the exposome? Environ. Int. 143, 105887. 10.1016/j.envint.2020.105887 32619912

[B176] Wang C. Ke C. Wang X. Chi C. Guo L. Luo S. (2014a). Noninvasive detection of colorectal cancer by analysis of exhaled breath. Anal. Bioanal. Chem. 406, 4757–4763. 10.1007/s00216-014-7865-x 24820062

[B177] Wang C. Sun B. Guo L. Wang X. Ke C. Liu S. (2014b). Volatile organic metabolites identify patients with breast cancer, cyclomastopathy, and mammary gland fibroma. Sci. Rep. 4, 5383. 10.1038/srep05383 24947160 PMC4064322

[B178] Wang Y. Hu Y. Wang D. Yu K. Wang L. Zou Y. (2012). The analysis of volatile organic compounds biomarkers for lung cancer in exhaled breath, tissues and cell lines. Cancer Biomarkers 11, 129–137. 10.3233/CBM-2012-00270 23144150 PMC13016210

[B179] Westphal K. Dudzik D. Waszczuk-Jankowska M. Graff B. Narkiewicz K. Markuszewski M. J. (2023). Common strategies and factors affecting off-line breath sampling and volatile organic compounds analysis using thermal desorption-gas chromatography-mass spectrometry (TD-GC-MS). Metabolites 13, 8. 10.3390/metabo13010008 PMC9866406 36676933

[B180] White I. R. Fowler S. J. (2019). “Capturing and storing exhaled breath for offline analysis,” in Breath analysis. Editors Pennazza G. Santonico M. (Amsterdam, Netherlands: Elsevier), 13–31. 10.1016/B978-0-12-814562-3.00002-3

[B181] WHO Coronavirus (COVID-19) Dashboard (2023). World health organization. Available at: https://covid19.who.int/ (Accessed June 13, 2023).

[B182] Wilde M. J. Cordell R. L. Salman D. Zhao B. Ibrahim W. Bryant L. (2019). Breath analysis by two-dimensional gas chromatography with dual flame ionisation and mass spectrometric detection – method optimisation and integration within a large-scale clinical study. J. Chromatogr. A 1594, 160–172. 10.1016/j.chroma.2019.02.001 30755317 PMC6491496

[B183] Wilkinson M. White I. R. Goodacre R. Nijsen T. Fowler S. J. (2020). Effects of high relative humidity and dry purging on VOCs obtained during breath sampling on common sorbent tubes. J. Breath. Res. 14, 046006. 10.1088/1752-7163/AB7E17 32153262

[B184] Wojnowski W. Dymerski T. Gębicki J. Namieśnik J. (2019). Electronic noses in medical diagnostics. Curr. Med. Chem. 26, 197–215. 10.2174/0929867324666171004164636 28982314

[B185] Woollam M. Angarita-Rivera P. Siegel A. P. Kalra V. Kapoor R. Agarwal M. (2022a). Exhaled VOCs can discriminate subjects with COVID-19 from healthy controls. J. Breath. Res. 16, 036002. 10.1088/1752-7163/ac696a 35453137

[B186] Woollam M. Siegel A. P. Grocki P. Saunders J. L. Sanders D. B. Agarwal M. (2022b). Preliminary method for profiling volatile organic compounds in breath that correlate with pulmonary function and other clinical traits of subjects diagnosed with cystic fibrosis: a pilot study. J. Breath. Res. 16, 027103. 10.1088/1752-7163/ac522f 35120338

[B187] Xu M. Tang Z. Duan Y. Liu Y. (2016). GC-based techniques for breath analysis: current status, challenges, and prospects. Crit. Rev. Anal. Chem. 46, 291–304. 10.1080/10408347.2015.1055550 26529095

[B188] Xu Z. Q. Broza Y. Y. Ionsecu R. Tisch U. Ding L. Liu H. (2013). A nanomaterial-based breath test for distinguishing gastric cancer from benign gastric conditions. Br. J. Cancer 108, 941–950. 10.1038/bjc.2013.44 23462808 PMC3590679

[B189] Zhang Y. Guo L. Qiu Z. Lv Y. Chen G. Li E. (2020a). Early diagnosis of breast cancer from exhaled breath by gas chromatography-mass spectrometry (GC/MS) analysis: a prospective cohort study. J. Clin. Lab. Anal. 34, e23526. 10.1002/jcla.23526 33150682 PMC7755810

[B190] Zhang Y. Shi L. Simoff M. J. Wagner O. J. Lavin J. (2020b). Biopsy frequency and complications among lung cancer patients in the United States. Lung Cancer Manag. 9, LMT40. 10.2217/lmt-2020-0022 33318758 PMC7729592

[B191] Zou Y. Hu Y. Jiang Z. Chen Y. Zhou Y. Wang Z. (2022). Exhaled metabolic markers and relevant dysregulated pathways of lung cancer: a pilot study. Ann. Med. 54, 790–802. 10.1080/07853890.2022.2048064 35261323 PMC8920387

[B192] Zou Y. Wang Y. Jiang Z. Zhou Y. Chen Y. Hu Y. (2021). Breath profile as composite biomarkers for lung cancer diagnosis. Lung Cancer 154, 206–213. 10.1016/j.lungcan.2021.01.020 33563485

[B193] Zou Y. Zhang X. Chen X. Hu Y. Ying K. Wang P. (2014). Optimization of volatile markers of lung cancer to exclude interferences of non-malignant disease. Cancer Biomarkers 14, 371–379. 10.3233/CBM-140418 25171479 PMC12928333

