# Supplementary material for: GC-MS-based metabolomics of volatile organic compounds in exhaled breath: applications in health and disease. A review
Source: Front Mol Biosci. 2024 Jan 8;10:1295955. doi: 10.3389/fmolb.2023.1295955 (PMC10828970; doi:10.3389/fmolb.2023.1295955)
Supplement: Supplementary file 1 [file Table1.DOCX]

Abbreviations

A-AD Asthma with atopic diseases

ABS Adaptive Breath sampler

AFP Alpha-fetoprotein

AHI Apnea-hypopnea index

AUC Areas under the curve

BAL Bronchoalveolar lavage

BC Breast cancer

BCA Breath Collection Apparatus

BNMD Breast non-malignant disease

CA10-9/CA72-4 Carbohydrate antigens

CAP Community-acquired pneumonia

Car Carboxen

CCQ Clinical chronic obstructive pulmonary disease Questionnaire

CEA Carcinoembryonic antigen

CF Cystic fibrosis

CFTR Cystic fibrosis transmembrane conductance regulator

CN culture-negative

COPD Chronic obstructive pulmonary disease

COPD (A) Acute exacerbation in chronic obstructive pulmonary disease

COPD (S) Stable chronic obstructive pulmonary disease

CP culture-positive

CRC Colorectal cancer

CT Computed tomography

CYP Cytochrome P450

DBT Digital breast tomosynthesis

DCIS Ductal carcinoma in situ

DVB Divinylbenzene

EBC Exhaled breath condensate

EPA Environmental Protection Agency

FeNO Fractional exhaled nitric oxide

FEV1pp Forced expiratory volume in one second

FIT Fecal immunochemical test

FS Feature selection

FU COVID-19 Follow-up samples of COVID-19 patients

GaC Gastric cancer

GC-MS Gas chromatography coupled to mass spectrometry

gFOBT Guaiac-based fecal occult blood test

HAP Hospital-acquired pneumonia

HBDB Human Breathomics Database

HC Healthy controls

ICU Intensive care unit

IMR-MS Ion molecule reaction-mass spectrometry

IMS Ion mobility spectrometry

IS Internal standard

LC Lung cancer

LDCT Low dose computed tomography

LNMN lymph node metastasis-negative

LNMP lymph node metastasis-positive

LOOCV Leave-one-out cross-validation

MRI Magnetic resonance imaging

MSI Metabolomics Standard Initiative

MVA Multivariate analysis

mVOCs Microbial volatile organic compounds

MW Molecular weight

NA-AD No-asthma with atopic diseases

NA-NAD No-asthma without atopic diseases

NSCLC Non-small cell lung cancer

NTD Needle-trap device

OLGIM Operative link on gastric intestinal metaplasia

OS Oxidative stress

OSA Obstructive sleep apnea

PDMS Polydimethylsiloxane

PET Positron emission tomography

PEx Pulmonary exacerbations (cystic fibrosis)

PNMD Pulmonary non-malignant disease

PNN Probabilistic neural network

ppmv Parts-per-million by volume

pptv Parts-per-trillion by volume

PPV Positive predictive value

PTR-MS Proton transfer reaction-mass spectrometry

PUD Peptic ulcer disease

PUFAs Polyunsaturated fatty acids

RF Random forest

ROS Reactive oxygen species

RT-PCR Reverse transcription polymerase chain reaction

SCLC Small cell lung cancer

SESI-MS Secondary electrospray ionization-mass spectrometry

SIFT-MS Selected ion flow tube-mass spectrometry

SP Stationary phase

SPME Solid-phase microextraction

SpO2 Percutaneous oxygen saturation

SVM Support vector machine

TD Thermal desorption tube

UVA Univariate analysis

VAP Ventilator-associated pneumonia

VOCs Volatile organic compounds

WHO World Health Organization
