# Supplementary material for: GC-MS-based metabolomics of volatile organic compounds in exhaled breath: applications in health and disease. A review
Source: Front Mol Biosci. 2024 Jan 8;10:1295955. doi: 10.3389/fmolb.2023.1295955 (PMC10828970; doi:10.3389/fmolb.2023.1295955)
Supplement: Supplementary file 2 [file Table2.docx]

Supplementary Material

**GC-MS-based metabolomics of volatile organic compounds in exhaled breath. Applications in health and disease. A review.**

**María Bajo-Fernández^1^, Érica A. Souza-Silva^1^, Coral Barbas^1^, María Fernanda Rey-Stolle^1^, Antonia García^1^***

^1^Centre for Metabolomics and Bioanalysis (CEMBIO), Faculty of Pharmacy, Universidad San Pablo CEU, CEU Universities. Campus Monteprincipe, Boadilla del Monte, 28668, Madrid, Spain.

*** Correspondence:**

**A**ntonia García Fernández
[antogar@ceu.es](mailto:antogar@ceu.es)

# Supplementary Figures and Tables

## Supplementary Tables

**Table S1**. Summary of cancer-focused studies.

**Table S2**. Summary of group comparisons, statistical approaches and reported VOCs in the cancer-focused studies.

**Table S3**. VOCs reported in lung cancer, gastric, colorectal and breast cancer (≥ 2 studies).

**Table S4**. Summary of group comparisons, statistical approaches and identified (VOC) in the studies focused on asthma, chronic obstructive pulmonary disease, obstructive sleep apnea and cystic fibrosis.

| **Table S1.** Summary of cancer-focused studies. *AB* alveolar breath, *BC* breast cancer, *COPD* chronic obstructive pulmonary disease, *CRC* colorectal cancer, *GaC* gastric cancer, *GC* gas chromatography, *GCxGC* two-dimensional gas chromatography, *IS* internal standard, *LC* lung cancer, *MB* mixed breath, *MS* mass spectrometry, *na* not applicable, *nd* not detailed, *NIST* National Institute of Standards and Technology, *SPME* solid-phase microextraction, *STD* standard, *TD* thermal desorption tube, *TOF* time-of-flight. | | | | | | | | | | |
| --- | --- | --- | --- | --- | --- | --- | --- | --- | --- | --- |
| **Reference** | **Pathology** | **Methodology** | **Sample** | **Sampling** | **Analysis technique** | **Sorbent material** | **Column** | **IS** | **Identification** | |
|  |  |  |  |  |  |  |  |  | **Library** | **Authentic STD** |
| (Chen et al., 2021) | LC | Untargeted | AB | Breath reservoir | TD- GC-MS | Tenax TA | Rtx-5 (30 m x 0.25 mm x 0.25 µm) (Restek) | No | NIST | No |
| (Saidi et al., 2020) | LC | Untargeted | AB | Bio-VOC® | SPME-GC-QTOF-MS | DVB/Car/PDMS | HP-5MSI (30 m x 0.25 mm x 0.25 µm) (Agilent) | No | nd | No |
| (Rudnicka et al., 2019) | LC | Untargeted | AB | Tedlar® bag | SPME-GC-MS | Car/PDMS 75 µm | CP-Porabond-Q (25 m × 0.25 mm × 3 μm) (Varian) | No | nd | Yes |
| (Wang et al., 2018) | LC | Untargeted | AB | Tedlar® bag | SPME/TD-GC-MS | PDMS 100 μm (SPME) and Tenax TA (TD) | Rtx-5 (30 m x 0.25 mm x 0.25 µm) (Restek) | No | NIST | No |
| (Sakumura et al., 2017) | LC | Untargeted | AB | Analytical Barrier Bag | TD-GC-MS | Tenax TA | DB-1 (Agilent) | No | nd | No |
| (Nardi-Agmon et al., 2016) | LC | Untargeted | AB | Mylar bag | TD-GC-MS | Tenax | SLB-5ms (30 m x 0.25 mm x 0.5 μm) (Supelco) | No | NIST | Yes |
| (Filipiak et al., 2014) | LC | Untargeted | AB | Tedlar® bag | TD-GC-MS | Tenax TA/Carboxen 569/Carboxen 1000 | PoraBond Q (25 m × 0.32 mm × 5 μm) (Varian) | No | NIST | Yes |
| (Rudnicka et al., 2014) | LC | Untargeted | AB | Tedlar® bag | SPME-GC-MS | Car/PDMS 75 µm | CP-Porabond-Q (25 m × 0.25 mm × 3 μm) (Varian) | No | nd | Yes |
| (Zou et al., 2014) | LC | Untargeted | AB | Tedlar® bag/BCA | SPME/TD-GC-MS | PDMS 100 µm (SPME) and Tenax TA 200 mg/Carbosieve S-III 50 mg (TD) | Rtx-5 (30.0 m x 0.25 mm x 0.25 μm) (Restek) | No | NIST | Yes |
| (Buszewski et al., 2012) | LC | Untargeted | AB | Tedlar® bag | SPME-GC-TOF-MS | Car/PDMS | CP-Porabond-Q (25 m × 0.25 m × 3 μm) (Agilent) | No | nd | Yes |
| (Wang et al., 2012) | LC | Untargeted | AB | Tedlar® bag | SPME-GC-MS | PDMS 100 µm | Rtx-1 (30 m × 0.25 mm × 0.25 µm) (Restek) | No | NIST | No |
| (Zou et al., 2022) | LC | Untargeted | MB | Self-Developed Device | TD-GC-MS | Tenax TA | Rtx-5 (30.0 m x 0.25 mm x 0.25 μm) (Restek) | No | NIST | No |
| (Gashimova et al., 2021) | LC | Untargeted | MB | Tedlar/Mylar bags | TD-GC-MS | Tenax TA | Supel-Q PLOT (30 m x 0.32 mm) (Supelco) | No | NIST | Yes |
| (Koureas et al., 2021) | LC | Untargeted | MB | Tedlar® bag | SPME-GC-MS | Car/PDMS 75 µm | DB-624 (30 m x 0.25 mm x 1.4 μm) (Agilent) | No | NIST / Restek database | Yes |
| (Zou et al., 2021) | LC | Untargeted | MB | Self-developed device | TD-GC-MS | Tenax TA | Rtx-5 (30.0 m x 0.25 mm x 0.25 μm) (Restek) | No | NIST | No |
| (Pesesse et al., 2019) | LC | Untargeted | MB | Tedlar® bag | TD-GCxGC-TOF-MS | Tenax GR/Carbopack B | Rxi-5Sil (30m × 0.25mm × 0.25 μm) (Restek) D^1^ and BPX-50 (1.2 m × 0.10mm × 0.10 μm) (SGE) D^2^ | No | Wiley and NIST | No |
| (Ligor et al., 2015) | LC | Untargeted | MB | Tedlar® bag | SPME-GC-MS | Car/PDMS | CP-Porabond-Q (25 m × 0.25 mm × 3 μm) (Varian) | No | NIST | No |
| (Monedeiro et al., 2021) | LC / COPD / Asthma | Untargeted / targeted | MB | Tedlar® bag | NTD-GC-MS | PDMS/Carbopack/Carboxen | DB-624 capillary column (60 m x 0.32 mm x 1.8 μm) (Agilent) | No | NIST | Yes |
| (Muñoz-Lucas et al., 2020) | LC | Targeted | AB | Bio-VOC® | TD-GC-MS | Tenax TA/Graphitized Carbon Black/Carbonized Molecular Sieve | DB-1 (30 m × 0.25 mm × 1 μm) (Agilent) | Hexamethylcyclotrisiloxane | NIST | Yes |
| (Callol-Sanchez et al., 2017) | LC | Targeted | AB | Bio-VOC® | TD-GC-MS | Tenax TA/Graphitized Carbon Black/Carbonized Molecular Sieve | DB-1 (30 m × 0.25 mm × 1 μm) (Agilent) | Hexamethylcyclotrisiloxane | NIST | Yes |
| (Corradi et al., 2015) | LC | Targeted | AB | Bio-VOC® | SPME-GC-MS | CAR/PDMS 75 µm and PDMS/DVB 65 µm | HP-5 MS (30 m × 0.25 mm x 0.50 µm) (Agilent) | n-Heptane-d16, Styrene-d8 and 2-Methylpentanal | na | Yes |
| (Koureas et al., 2021) | LC | Targeted | MB | Tedlar® bag | SPME-GC-MS | CAR/PDMS 75 µm | DB-624 (30 m x 0.25 mm x 1.4 μm) (Agilent) | No | na | Yes |
| (Schallschmidt et al., 2016) | LC | Targeted | MB | Gas bulb | SPME-GC-MS | CAR/PDMS 75 µm | VF-624ms (60 m x 0.32 mm x 1.8 μm) (Varian) | No | na | Yes |
| (Bhandari et al., 2023) | GaC | Untargeted | AB | In-house custom-made Breath Sampler | TD-GC-MS | Tenax TA | Rxi-624Sil MS (30 m × 0.32 mm x 1.8 µm) (Restek) | No | NIST | Yes |
| (Amal, Leja, Funka, Skapars, et al., 2016) | GaC | Untargeted | AB | GaSampler collection bag (QuinTron) | TD-GC-MS | Tenax TA | SLB-5ms (30 m x 0.25 mm x 0.5 µm) (Supelco) | No | NIST | Yes |
| (Amal et al., 2013) | GaC | Untargeted | AB | Mylar bag | TD-GC-MS | Tenax TA 100/50 mg | SLB-5 ms (30 m x 0.25 mm x 0.5 μm) (Supelco) | No | NIST | Yes |
| (Xu et al., 2013) | GaC | Untargeted | AB | Tedlar® bag | TD-GC-MS | Tenax TA | SLB-5ms capillary column (30 m x 0.25 mm x 0.5 μm) (Supelco) | No | NIST | Yes |
| (Tong et al., 2017) | GaC | Untargeted | MB | Gas-Tight Syringe | SPME-GC-MS | Car/PDMS 75 µm | DB-5MS (30 m x 0.25 mm x 0.25 µm) (Agilent) | No | NIST | No |
| (Altomare et al., 2020) | CRC | Untargeted | AB | ReCIVA® | TD-GC-MS | Tenax/Carbograph | DB-624 (30 m x 0.25 mm x 1.4 μm) (Agilent) | No | NIST | Yes |
| (Amal, Leja, Funka, Lasina, et al., 2016) | CRC | Untargeted | AB | GaSampler Collection Bag (QuinTron) | TD- GC-MS | Tenax TA 100/50 mg | SLB-5ms (30 m x 0.25 mm x 0.5 μm) (Supelco) | No | NIST | Yes |
| (C. Wang, Ke, et al., 2014) | CRC | Untargeted | AB | Gas-tight Syringe | SPME-GC-MS | Car/PDMS 75 µm | DB-5MS (30 m x 0.25 mm x 0.25 µm) (Agilent) | No | NIST | No |
| (Altomare et al., 2015) | CRC | Untargeted | MB | Tedlar® bag | TD-GC-MS | Carboxen/Carbopack | HP-5 MS (30 m × 0.25 mm x 0.50 µm) (Agilent) | No | NIST | No |
| (Altomare et al., 2013) | CRC | Untargeted | MB | Tedlar® bag | TD-GC-MS | Carboxen/Carbopack | SUPELCOWAX (30 m × 0.25 mm x 0.25 μm) (Supelco) | No | NIST | No |
| (Zhang et al., 2020) | BC | Untargeted | AB | Gas-tight syringe | SPME-GC-MS | 75 mm SPME fiber | DB-5MS (30 m x 0.25 mm x 0.25 µm) (Agilent) | No | nd | No |
| (Barash et al., 2015) | BC | Untargeted | AB | Tedlar® bag | TD-GC-MS | Tenax TA | SLB-5 ms capillary column (30 m x 0.25 mm x 0.5 μm) (Supelco) | No | NIST | Yes |
| (C. Wang, Sun, et al., 2014) | BC | Untargeted | AB | Gas-tight syringe | SPME-GC-MS | C/PDMS 75 µm | DB-5MS (30 m x 0.25 mm x 0.25 µm) (Agilent) | No | NIST | No |
| (Li et al., 2014) | BC | Targeted | AB | Bio-VOC®/ Tedlar® bag | GC-MS | na | DB-624 capillary column (60 m x 0.25 mm x 1.4 μm) (Agilent) | No | NIST | Yes |

| **Table S2**. Summary of group comparisons, statistical approaches and reported VOCs in the cancer-focused studies. *a* altered, *BC* breast cancer, *BL* baseline sample, *BNMD* breast non-malignant disease, *BS* breath sample, *CMP* cyclomastopathy, *COPD* chronic obstructive pulmonary disease , *CRC* colorectal cancer, CRC-AS colorectal cancer after surgery, *CRC-BS* colorectal cancer before surgery, *CTR* control, *DC* disease control, *DCIS* ductal carcinoma in situ, down downregulated, *GaC* gastric cancer, *HC* healthy controls, *LC - COPD* lung cancer without chronic obstructive pulmonary disease , *LC + COPD* lung cancer with chronic obstructive pulmonary disease , *LC* lung cancer, *LNMN* lymph node metastasis-negative, *LNMP* lymph node metastasis-positive, *MGF* mammary gland fibroma, *MVA* multivariate analysis, *non-LC* non-lung cancer subjects, *OLIGM 0-IV* Operative link on gastric intestinal metaplasia assessment stages 0-IV, *PD* progressive disease, *PNMD* pulmonary non-malignant disease, *PUD* peptidic ulcer disease, *up* upregulated, *UVA* univariate analysis, | | | | | |
| --- | --- | --- | --- | --- | --- |
| **Reference** | **Pathology** | **Comparison** | **Statistical approach** | **Significant VOCs** | **Details** |
| (Chen et al., 2021) | LC | LC (n=160) vs PNMD (n=70) vs HC (n=122) | UVA/MVA | (-)-Cedrene**** (a), (+)-Longifolene**** (a), (1-Octylnonyl) cyclohexane*** (a), (E)-371,115-Tetramethyl-2-hexadedcen-1-ol*** (a), 1,16-Dichlorohexadecane*** (a), 1,2,3,3a,4,9,10,10a-Octahydrobenz[f]azulene**** (a), 1,2,3-Trimethylbenzene (a), 1,2-Dichloroethane** (a), 1,4-Methanoazulen-9-ol, decahydro-1, 5,5,8a-tetramethyl-, (1R, 3aR, 4S, 8aS, 9S)** (a), 13-Isopropylpodocarpa-81,113-trien-19-al, 4-methyloctane**** (a), 1-Chlorooctadecane*** (a), 1-Chlorooctane*** (a), 1-Chlorotetradecane*** (a), 1-Hexanol, 5-methyl-2-(1-methylethyl)*** (a), 1-Hexoxyoctane**** (a), 1-Isopropyl-7-methyl-4-methylene-1,2,3,4,4a,5,6,8a-Octhydraonaphthalene*/***/***** (a), 1-Methoxy-4-(Z)-prop-1-enyl-benzene*** (a), 1-Methyl-3-propylbenzene (a), 1-Octanol*** (a), 2,2,3-Trimethylnonane**** (a), 2,4-Dimethylhexane** (a), 2,4-Hexadiyne** (a), 2,5-Dimethylfuran (a), 2-Ethyl-p-xylene*/***** (a), 2-Methylbutyric acid** (a), 2-Nonenal, (2E)** (a), 3-Methylidenenonane**** (a), 4-Ethylcumen*** (a), 5,5-Dibutylnonane**** (a), 6-Tert-Butyl-m-cresol**** (a), 8-Methylheptadecane**** (a), Acetophenone**** (a), Benzene*/** (a), Benzene, (1-methylethyl)*** (a), Benzene, 1-ethyl-2, 3-dimethyl*** (a), Benzene, 4-ethyl-1, 2-dimethyl (a), Benzoic acid, 3, 5-bis (1,1-dimethylethyl)** (a), Benzothiazole (a), Butyric acid** (a), Camphor**** (a), Cedrene*** (a), Cineole*** (a), Cyclohexane, heptyl** (a), Cyclononasiloxane, octadecamethyl -(8CI, 9CI);** (a), Decamethyltetrasiloxane**** (a), Dibutyl phthalate**** (a), Di-tert-butyl peroxide** (a), DL-sec-Butyl acetate (a), Dodecane, 4,6-Dimethyl*** (a), Ethylbenzene (a), Heptanal** (a), Heptane*/** (a), Heptane, 3-ethyl-3-methyl (a), Hexamethylcyclotrisiloxane (a), Hexanal (a), Hydroxybenzene**** (a), Methyl 10-methylundecanoate** (a), Methyl laurate**** (a), Methylcyclohexane (a), n-Nonane Nonane nonyl hydride (a), n-Propylbenzene (a), Octadecamethylcyclononasiloxane** (a), Octane, 4-methyl**** (a), O-Xylene (a), Propyl acetate (a), Propylcyclohexane*/** (a), p-Terphenyl** (a), Tetrachloroethylene** (a), Tetrapentacontane*** (a), Tridecane, 2-methyl*** (a), Undecane, 3-methyl*** (a), β-Methyllevulinic Acid*** (a) | *Common VOCs among comparisons; **Significant VOCs between LC and PNMD; ***Significant VOCs between NSCLC and SCLC; ****Significant VOCs early-stage LC and advanced-stage LC |
| (Saidi et al., 2020) | LC | LC (n=32) vs HC (n=12) | UVA | Pentanoic acid (up), N-[4-Bromo-n-butyl]-2-piperidinone (up), 2-Isopropyl-5-methyl-1-heptanol (up), 5,5-Dimethyl-undecane (up) | VOCs only detected in LC |
| (Rudnicka et al., 2019) | LC | LC (n=108) vs HC (n=121) | UVA/MVA | 1,4-Pentadiene* (a), 1-Pentene (a), 2,3,4-Trimethylhexane (a), 2,3-Butanedione (a), 2,4-Dimethylheptane* (a), 2-Methyl-2-butene (a), 2-Methylfuran* (a), 2-Methylheptane* (a), 2-Pentanone* (a), 2-Propanol (a), 4,7-Dimethylundecan (a), 4-Heptanone* (a), 4-Methyloctane* (a), Acetone* (a), Acetonitrile* (a), Carbon disulfide (a), Cyclohexane* (a), Cyclohexanone* (a), Dimethyl sulﬁde* (a), Dodecane* (a), Ethyl acetate* (a), Ethylbenzene (a), Hexanal (a), Hexane* (a), Isobutane* (a), Isoprene* (a), Limonene (a), Methyl acetate* (a), Methyl vinyl ketone* (a), Nonane (a), o-Xylene (a), Propane* (a), p-Xylene* (a), β-Pinene (a) | *Selected VOCs to build the MVA |
| (M. Wang et al., 2018) | LC | LC (n=233) vs PNMD (n=111) vs HC (n= 140) | UVA/MVA | 3-Ethyltoluene (a), 1,2,3-Trimethylbenzene (a), *n*-Propylbenzene (a), Propylcyclohexane (a), Indan (a), 1-Methyl-3-Propylbenzene (a), o-Xylene (a), 4-Methyl-2-pentanone (a), 5-Methylindan (a), Methylcyclohexane (a), Heneicosane (a) | *VOCs detected by SPME |
| (Sakumura et al., 2017) | LC | LC (n=107) vs HC (n=29) | UVA/MVA | 1-propanol (a), Acetonitrile (a), Acetonitrile (a), Chloroform (a), Ethanol (a), Hydrogen cyanide (a), Isoprene (a), Methanol (a) |  |
| (Nardi-Agmon et al., 2016) | LC | LC (n=39): BL (BS=39) vs DC (BS=85) vs PD (BS=11) | UVA | Dodecane,4-methyl (down), Styrene* (down), α-Phellandren (down) | *Significant VOC PD vs DC |
| (Filipiak et al., 2014) | LC | LC (n=36) vs HC (n=28) | UVA | 1(R)-a-Pinene (up), 2,3-butanedione (up), 2,4-Dimethyl-1-heptene (down), 2-Methylpentane (up), 2-Methylpropanal (up), 2-Propanol (up), 2-Propenal (acrolein) (up), 3-Methylhexane (up), 6-Methyl-5-heptene-2-one (down), Acetaldehyde* (up), Acetic acid (up), Butanedione (up), Decanal (down), Ethanol* (up), Hexanal (up), Hexane (up), Methanol (down), Methyl acetate (down), n-Dodecane (up), Nonane (up), Octanal (down), Octane* (up), Propanal (up), Propene (up) | *VOCs found significant in in vitro cultures |
| (Rudnicka et al., 2014) | LC | LC (n=108) vs PNMD (n=24) vs HC (n=121) | MVA | 1,4-Pentadiene (a), 2,4-Dimethylheptane (a), 2-Propanol (up), Acetyl ethyl (a), Dimethyl sulphide (up), Isobutane (a) |  |
| (Zou et al., 2014) | LC | LC (n=137) vs PNMD (n=54) vs HC (n=38) vs non-LC (n=20) | UVA/MVA | Dodecane, 2, 6, 11-trimethyl- (a), Hexadecanal (a), Nonane, 5-(2-methyl) propyl- (a), Pentadecane, 8-hexyl- (a), Phenol, 2,6-di-tert-butyl-, 4-methyl- (a), Tridecane* (a) | *Significant VOC between PNMD and HC |
| (Buszewski et al., 2012) | LC | LC (n=29) vs HC (n=44) | UVA | 1-Propanol* (up), 2-Butanone* (up), 2-Pentanone* (up), 2-Propanol* (up), 2-Propenal (up), Acetone (up), Benzene (up), Butanal* (up), Ethyl acetate* (up), Ethylbenzene* (up), Furan (up), Propanal (up) | * Significant VOCs LC vs HC smokers |
| (Y. Wang et al., 2012) | LC | LC (n=85) vs PNMD (n=70) vs HC (n=88) | UVA/MVA | 2,6- Di-tert-butyl-, 4-methylphenol (a), 2,6,10,14-Tetramethylpentadecane (a), 2,6,11-Trimethyldodecane (a), 2,6-Dimethylnaphthalene (a), 2-Methylhendecanal (a), 2-Methylnaphthalene (a), 2-Pentadecanone (a), 3,7-Dimethylpentadecane (a), 3,8-Dimethylhendecane (a), 4-Methyltetradecane (a), 5-(1-Methyl-)propylnonane (a), 5-(2-Methyl-)propylnonane (a), 5-Butylnonane (a), 5-Propyltridecane (a), 7-Methylhexadecane (a), 8-Hexylpentadecane (a), 8-Methylheptadecane (a), Eicosane (a), Hexadecanal (a), Nonadecane (a), Nonadecanol (a), Tridecane (a), Tridecanone (a) |  |
| (Zou et al., 2022) | LC | LC (n=60) vs HC (n=176) | UVA/MVA | L-Menthol* (down), 1-Methylnaphthalene* (down), 1-Octene (up), 2,5-Dimethylfuran (down), 2-Butoxyethanol (up), 2-Ethylhexanol (a), 3-(Methylthio)-1-propene (down), 3,3-Dimethylhexane (a), 3-Carene (down), 3-Ethyltoluene (down), 3-Methylbutanol (up), 3-Methylheptane (down), Acetic acid* (down), Camphene* (down), Dimethyldisulfide (down), Eucalyptol* (a), Hexanal* (up), Isopropylbenzene (down), Limonene (down), n-Butylacetate (up), n-Heptane (up), n-Nonanal (up), n-Nonane (a), n-Octanal (up), n-Octanol (up), n-Undecane (a), o-Cymene (down), o-Xylene* (a), p-Cymene* (a), Phytol (a), Propyl acetate (up) | *VOCs found to be involved in metabolic pathways |
| (Gashimova et al., 2021) | LC | LC (n=40) vs HC (n=40) | UVA/MVA | 1-Butanol (a), 1-Methylthiopropene (a), 2-Pentanone (a), Benzene (a), Butyl acetate (a), Dimethyl disulfide (a), Dimethyl trisulfide (a), Hexanal* (a), Hexane (a), Octanal (a), Toluene (a) | *Most important VOC in MVA |
| (Koureas et al., 2021) | LC | LC (n=49) vs PNMD (n=36) vs HC (n=52) | UVA/MVA | 1-Methoxy-2-propanol (down), 1-Methylthio-(E)- 1-propene** (down), 3-Methyl-furan (a), Acetaldoxime**/*** (down), Acetic acid (down), Benzene (down), Dimethyl furane (a), Ethylbenzene (up), Eucalyptol** (a), Methyl propyl sulfide* (down), Methylacetamide (a), N-2-Aminoethyl acetamide*** (down), p-Benzoquinone (a), Propionic acid (down), Styrene (up), Thiophene**** (a), Toluene (up), Xylene (p,o,m) (up) | *Significant VOC in LC vs HC and LC vs PNMD; ** Discriminant (MVA) VOCs between LC and HC; ***Discriminant (MVA) VOCs LC and PNMD; ****Discriminant VOC (MVA) from the targeted analysis between LC and PNMD |
| (Zou et al., 2021) | LC | LC (n=60) vs HC (n=176) | MVA | 3-Methyl-Hexane (a), 2,5-Dimethyl-Furan (a), Propyl Acetate (a), 2,4-Dimethyl-Heptane (a), 1,3,5,7-Cyclooctatetraene (a), Isopropyl-Benzene (a), Styrene (a), β-Pinene (a), 2,2,4,6,6-Pentamethyl-Heptane (a), 1,2,3-Trimethyl-Benzene (a), Octanal (a), 1,3-Dichloro-Benzene (a), 1-Ethyl-3-Methyl-Benzene (a), 2,2,4,4,6,8,8-Heptamethyl-Nonane (a), 3,7-Dimethyl-Decane (a), Decane (a), Phenylpropyne (a), 2-Ethyl-Hexene (a), 1-Ethyl-2,3-Dimethyl-Benzene (a), 15,19-Dimethyl-Tritriacontane (a), Isoamyl Acetate (a), Hexamethyldisiloxane (a) |  |
| (Pesesse et al., 2019) | LC | LC (n=15) vs HC (n=14) | UVA/MVA | 1-Decene (a), 1-Methyl-2-methylenecyclohexane* (a), 1-Undecene (a), 2,5-cyclohexadien-1-one, 2,6-bis(1,1-dimethylethyl)-4-hydroxy-4-methyl- (a), 2,5-Cyclohexadiene-1,4-dione, 2,6-bis(1,1-dimethylethyl)- (a), 2-Cyclohexen-1-ol, 1-methyl-4-(1-methylethenyl)-, trans- (a), 2-Hexene, 3,5,5-trimethyl- (a), 3-Heptene, 2-methyl-, (E)- (a), 3-Tridecene, (Z)- (a), 4,5-Nonadiene (a), 4-Amino-1-butanol (a), 4-Hepten-2-one, (E)- (a), 4-Undecene, 5-methyl-, (E)-* (a), 5-Hepten-2-one, 6-methyl- (a), 7-Oxabicyclo[4.1.0]heptane, 2-methylene-* (a), Acetic acid, phenyl ester* (a), Anethole* (a), α--Pinene (a), Cyclohexane, isothiocyanato- (a), Cyclopentane, ethyl- (a), Cyclopentane, methyl- (a), Cyclopropane, 1,2-dimethyl-1-pentyl- (a), Cyclotetradecane (a), Decane (a), Diglycolamine (a), Eicosane (a), Furfural (1)* (a), Furfural (2) (a), Heptadecane, 2-methyl-1-Methyl-2-* (a), Hexadecane (a), Isoborneol (a), Levomenthol (a), n-Tridecan-1-ol (a), Octanoic acid, ethyl ester (1) (a), Octanoic acid, ethyl ester (2) (a), Pentadecane (a), Propanoic acid, phenyl ester (a) | *VOCs that appear in both UVA and MVA |
| (Ligor et al., 2015) | LC | LC (n=123) vs HC (n=361) | MVA | 2-Pentanone (up), Butane (a), Butane, 2-methyl (up), Heptane, 2,4-dimethyl (up), Octane, 4-methyl (a), Propanal (up), Propane (a), Propene (a) |  |
| (Monedeiro et al., 2021) | LC / COPD / Asthma | LC (n=16) vs COPD (n=12) vs Asthma (n=8) vs HC (n=20) | UVA/MVA | 1-Pentanol (a), (E)-Ocimene* (up), 1,2,4-Trimethylbenzene (up), 1-Propanol (up), 2-Butanone (up), 2-Methyl-1-propanol (a), 2-Methyldecane (a), 2-Methylpentane (up), 2-Propanol* (up), 3,3-Dimethyl-butanamide (a), 3-Amino-butanoic acid (a), 3-Methylpentane* (up), Acetoin (up, down asthma), Acetonitrile (a), Benzonitrile* (up), Dodecane (up), Eucalytol (a), Isododecane (up), Limonene* (up), m-Cymene* (up), Methyl vinyl ketone (a), Ocimene (a), Phenol* (up), Styrene (up), Terpineol* (up), Tetradecane (a), Tridecane (up), Undecane* (up) | *VOCs quantified and used to build the final model |
| (Muñoz-Lucas et al., 2020) | LC | LC + COPD (n=67) vs LC - COPD (n=40) | UVA | Propanoic acid (up) |  |
| (Callol-Sanchez et al., 2017) | LC | LC (n=81) vs COPD (n=40) vs HC (n=89) | UVA | Nonanoic acid (up) |  |
| (Corradi et al., 2015) | LC | LC (n=71) vs PNMD (n=67) | UVA/MVA | 2-methyl pentane (up), Ethyl benzene (up), Heptanal (up), Hexane* (up), Octanal (up), Pentane (up), Trans-2-heptanal (up), Trans-2-hexanal (up), Trans-2-nonenal* (up) | *VOCs with discriminant power in the MVA |
| (Koureas et al., 2021) | LC | LC (n=51) vs PNMD (n=38) vs HC (n=53) | UVA/MVA | 1-Butanol (a), 1-Propanol* (down), 2-Butanone (a), Acetone (a), Benzene (a), Cyclohexane (a), Cyclohexanone (a), Ethyl butyrate* (down), Ethylbenzene* (up), Hexanal (a), Hexane* (down), Isoprene (a), Isopropanol* (down), n-Octanal (a), Nonanal (a), Octane (a), Styrene* (up), Toluene* (up) | *VOCs discriminative in UVA among all groups |
| (Schallschmidt et al., 2016) | LC | LC (n=37) vs HC (n=23) | UVA/MVA | 1-Butanol, 2-Butanone, 2-Pentanone, Cyclohexane, Ethylbenzene, n-butanal, n-decanal, n-dodecane, n-heptane, n-hexanal, n-octane, n-pentanal, n-propylbenzene, Propanal |  |
| (Bhandari et al., 2023) | GaC | GaC (n=16) vs HC (n=33) | UVA/MVA | 1-Decanol*** (a), 1-Dodecene** (a), 1-Octanol*/** (down), 2-Pentanone** (a), 2-Propanol, 1-(2-methoxy-1-methylethoxy)-** (a), 3-Methylcyclopentyl acetate*** (a), 4-Ethylbenzamide*** (a), Benzene** (a), Benzene, 1-methyl-3-(1-methylethyl)-** (a), Benzyl alcohol*** (a), Dimethyl disulfide** (a), dl-Erythro-1-phenyl-1,2-propanediol*** (a), Dodecane, 5,8-diethyl-** (a), Ethanol, 2-phenoxy-** (a), Methacrolein*** (a), n-Decane** (a), n-Hexane** (a), Nonane** (a), Octane,1,1′-oxybis-*/** (down), Phenol, 2-methyl-4-(1,1,3,3-tetramethylbutyl)-*** (a), Toluene** (a) | *Significant VOCs in UVA; ** VOCs correlated with faecal bacteria species in GaC; *** VOCs correlated with faecal bacteria species HC |
| (Amal, Leja, Funka, Skapars, et al., 2016) | GaC | GaC (n=99) vs OLIGM 0-IV (n=325) vs PUD (n=53) | UVA | 1,2,3-Trimethylbenzene* (up), 2-Butanone* (up), 2-Butoxy-ethanol* (up), 2-Propenenitrile* (up), 4-Methyl octane** (up), Furfural (up), Hexadecane (up), α-Methyl-styrene* (up) | *Significant VOCs exclusively in GaC vs OLGIM (0-IV); **Significant VOCs exclusively PUD vs OLGIM (0-IV) |
| (Amal et al., 2013) | GaC | GaC (n=37) vs PUD (n=32) vs CTR (n=61) | UVA | 2-Ethyl-1-hexanol* (up), 6-methyl-5-hepten-2-one (up), Nonanal (up), Styrene* (up) | *Significant VOCs in GaC vs HC and PUD vs HC |
| (Xu et al., 2013) | GaC | GaC (n=37) vs PUD (n=32) vs CTR (n=61) | UVA | 2-Butoxy-ethanol** (up), 2-Propenenitrile (up), 6-Methyl-5-hepten-2-one* (up), Furfural* (up), Isoprene** (up) | *Significant VOCs in GaC/PUD vs CTR; **Significant VOCs in PUD vs CTR |
| (Tong et al., 2017) | GaC | GaC (n=24) vs PUD (n=24) vs Gastritis (n=48) vs HC (n=32) | UVA/MVA | 1,3-Dioxolan-2-one* (down), 1,3-Dioxolane-2-methanol**/*** (up), 1,6-Dioxacyclododecane-7,12-dione*** (down), 2,3-Butanediol, [R-(R*,R*)]-* (up), 3,5-Decadien-7-yne, 6-t-butyl-2,2,9,9-tetramethyl-*** (down), 5,7-Octadien-2-one, 3-acetyl-*** (down), 5-Hepten-2-one, 6-methyl-*** (down), Benzothiazole*** (up), Caprolactam*** (down), Hexadecane* (up), N,N-Dimethylacetamide**/*** (down), Nonanal*** (down), Phosphonic acid, (p-hydroxyphenyl)-**/*** (down), Undecane, 3,8-dimethyl-* (up) | *Significant VOCs GaC vs HC; ** Significant VOCs GaC vs PUD ***Significant VOCs GaC vs gastritis |
| (Altomare et al., 2020) | CRC | CRC (n=83) vs HC (n=90) | UVA/MVA | 5,9-Undecadien-2-one, 6,10-dimethyl ** (a), Acetic acid (a), Benzaldehyde**/*** (a), Benzene, 1,3-bis(1-methylethenyl) (a), Benzoic acid*** (a), Butanoic acid (a), Butyl hydroxy toluene** (a), Decanal (a), Decane (a), Decanoic acid (a), Dimethyl heptane (a), Dodecane** (a), Ethanol (a), Ethanone, 1[4-(1-methylethenyl)phenyl] (a), Ethylbenzene* (down), Hexanal (a), Methylbenzene* (up), Nonadecane (a), Nonanal (a), Nonanoic acid (a), Octanal (a), 4-Methyloctane (a), Pentadecane (a), Tetradecane* (down), Tridecane** (a), Undecane (a), Xylene (a) | * VOCs present in both UVA and MVA; ** Discriminant VOCs early-stage CRC vs HC; *** Discriminant VOCs advanced-stage CRC vs HC |
| (Amal, Leja, Funka, Lasina, et al., 2016) | CRC | CRC (n=65) vs HC (n=122) | UVA | 4-Methyloctane (down), Acetone (up), Ethanol (down), Ethyl acetate (up) |  |
| (C. Wang, Ke, et al., 2014) | CRC | CRC (n=20) vs HC (n=20) | UVA/MVA | 2,2-Dimethyldecane (up), 3-Hydroxy-2,4,4-trimethylpentyl 2-Methylpropanoate (up), 4-Ethyl-1-octyn-3-ol (up), 6-t-Butyl-2,2,9,9-tetramethyl-3,5-decadien-7-yne (down), Cyclohexanone (up), Cyclooctylmethanol (up), Dodecane (up), Ethylaniline (up), Trans-2-Dodecen-1-ol (up) |  |
| (Altomare et al., 2015) | CRC | CRC-BS (n= 52) vs CRC-AS (n=32) vs HC (n=55) | MVA | 1-(1-Methylethenyl)-2-(1-methylethyl)benzene (a), 1,2,3-Trimethylbenzene (a), 1,2-Pentadiene (a), 1,3-Dimethyl-5-(1-methylethyl)benzene (a), 1,3-Dimethylbenzene (a), 1,4-Dimethylbenzene (a), 1-Ethyl-1,2,4-trimethylbenzene (a), 1-Ethyl-2,4,5-trimethylbenzene (a), 1-Methyl-3- (1-methylethyl)benzene (a), 1-Methylnaphthalene (a), 1-Octene (a), 2,3-Dihydro-1,6-dimethyl-1H-indene (a), 2,3-Dihydro-4,7-dimethyl-1H- indene (a), 2-Methylbutane (a), 2-Methylnaphthalene (a), 2-Methylpentane (a), 4-Methyl-2-pentanone (a), 5-Butylnonane (a), Beta-pinene (a), Cyclohexane (a), Decanal (a), Dodecane (a), Heptane (a), Methylcyclohexane (a), Methylcyclopentane (a), Nonanal (a), Octane (a), Propylbenzene (a), Undecane (a) |  |
| (Altomare et al., 2013) | CRC | CRC (n=52) vs HC (n=51) | UVA/MVA | 1,2-Pentadiene (a), 1,3-Dimethylbenzene (up), 1,4-Dimethylbenzene (a), 2-Methylbutane (a), 2-Methylpentane (a), 3-Methylpentane (a), 4-Methyl-2-pentanone (a), 4-Methyloctane (up), Cyclohexane (up), Decanal (a), Methylcyclohexane (up), Methylcyclopentane (a), Nonanal (a) |  |
| (Zhang et al., 2020) | BC | BC(n=71)(DCIS n=13; LNMN n=31; LNMP n=27) vs GaC (n=54) vs HC (n=78) | MVA | 1,1,3,3-Tetramethylurea**/*** (a), 2,6-Dimethyloctane** (a), 2-Butoxyethanol*** (a), 2-Ethylhexanol**/*** (a), 2-Phenyl-2-propanol**/*** (a), 3-Methoxy-1,2-propanediol* (a), 3-Methylpyridine* (a), Cyclohexanol, 2-(1-methylethyl)-*** (a), Cyclohexanone**/*** (a), Cyclopentanone*/**/*** (a), Ethylene carbonate*/**/*** (a), Hexamethyldisilane** (a), Methyl acrylic acid**/*** (a), Phenol*/**/*** (a), Propane, 2-methyl-1,2- bis (trimethylsiloxy)-** (a), (S)-1,2-Propanediol*/**/*** (a), Tetramethyl silicane*/**/*** (a) | *Discriminative VOCs BC vs GaC; **Discriminative VOCs DCIS vs HC; *** Discriminative VOCs LNMN BC vs HC; All VOCs reported are discriminant for LNMP BC vs HC |
| (Barash et al., 2015) | BC | BC (n=169) vs BNMD (n=52) vs DCIS (n=25) vs HC (n=30) | UVA/MVA | 1-hexanol, 2-ethyl* (a), 2-Propenoic acid, butyl ester*/**/*** (a), 5-Hepten-2- one, 6-methyl-*/*** (a), Acetic acid, butyl ester*/**/*** (a), Acetone*/*** (a), Benzene, (1-methylethyl)-*** (a), Benzene, 1,3-dimethyl-*/**/*** (a), Carbonic acid, dimethyl ester*/**/*** (a), Cyclohexane, 1,4-dimethyl-* (a), Cyclopentane*/**/*** (a), Cyclopentane, methyl-**/*** (a), Ethanol*/**/***/**** (a), Ethyl Acetate**/***/**** (a), Ethylbenzene**/*** (a), Heptane*/** (a), Heptane, 2, 4-dimethyl-**/*** (a), Heptane, 2,3,4-trimethyl-***/**** (a), Hexanal**/*** (a), Pentane, 2,3-dimethyl-*/**/*** (a), Pentane, 3-methyl-**/*** (a), Styrene***/***** (a), Toluene*/**/***/**** (a), α-Pinene*/*** (a) | *Discriminant VOCs in MVA; **Significant VOCs in UVA HC + BNMD vs BC; *** Significant VOCs in UVA HC vs BC + BNMD ****Significant VOCs in UVA BC vs DCIS |
| (C. Wang, Sun, et al., 2014) | BC | BC (n=39) vs BNMD (n=46) vs HC (n=45) | UVA/MVA | 1,2-Propanediol*** (up), 1,3,5,7-Tetroxane* (down), 1,4-Dimethoxy-2,3-butanediol**** (up), 2,2-Dimethyl decane* (down), 2,3,4-trimethylheptane* (down), 2,3,6-Trimethyloctane* (down), 2,5,6-Trimethyloctane**** (up), 2,5-Dimethylhexane-2,5-dihydroperoxide* (down), 2-Acetyl aminopropionic acid * (down), 3,4,5,6-Tetramethyloctane*** (up), 4-Hydroxybutanoic acid* (down), 5-Butylnonane* (down), 5-Methyl-3-hexanol* (down), Benzenemethanol, alpha,alpha-dimethyl* (down), Benzocyclobutene* (down), Butyl acetate* (down), Butyl glycol**/*** (up), Cyclohexanone**** (up), Cyclooctanemethanol** (down), Cyclopentanone*** (down), Dimethylacetamide* (up), Ethylaniline*** (up), Ethylene carbonate* (down), Hexadecane* (down), Methylacrylic acid* (down), Tetradecane* (down), Trans-2-Butene oxide* (up), Trans-2-Dodecen-1-ol** (down) | *Significant VOCs BC vs HC; **Significant VOCs BC vs CMP; *** BC vs MGF; ****Significant VOCs in the 3 comparisons |
| (Li et al., 2014) | BC | BC (n=22) vs BNMD (n=17) vs HC (n=24) | UVA/MVA | Heptanal (up), Hexanal** (up), Nonanal* (up), Octanal (up) | *Upregulated VOC BC vs BNMD; ** Upregulated BNMD vs HC |

| **Table S3**. VOCs reported in lung cancer, gastric, colorectal and breast cancer (≥ 2 studies). *na* not applicable, *ng/L* nanograms per liter, *pM* picomolar, *ppb* parts per billion, *ppbv* parts per billion by volume. ^a^Alveolar gradient (expired – inspired air), *LOD, limit of detection. | | | | | | | | | | |
| --- | --- | --- | --- | --- | --- | --- | --- | --- | --- | --- |
| **Lung cancer** | | | | | | | | | | |
| **Nº** | **Compound name** | **CAS-N** | **Formula** | **Chemical class** | | **Sign of alteration** | **Concentration (patients)** | **Concentration (controls)** | **Unit** | **Reference** |
| 1 | 1-Butanol | 71-36-3 | C_4_H_10_O | Alcohol | | Altered | na | na | na | (Gashimova et al., 2021) |
|  |  |  |  |  |  | Altered | <0.56*-1.05 | <0.56*-1.41 | ng/L | (Koureas et al., 2020) |
|  |  |  |  |  |  | Upregulated | 1.4–41.0 | 0.5–19.4 | ng/L | (Schallschmidt et al., 2016) |
| 2 | 1-Nonadecanol | 1454-84-8 | C_19_H_40_O | Alcohol | | Altered | na | na | na | (Y. Wang et al., 2012) |
|  |  |  |  |  |  | Altered | na | na | na | (Zou et al., 2014) |
| 3 | 1-Octanol | 111-87-5 | C_8_H_18_O | Alcohol | | Altered | na | na | na | (Chen et al., 2021) |
|  |  |  |  |  |  | Altered | na | na | na | (Zou et al., 2022) |
| 4 | 1-Propanol | 71-23-8 | C_3_H_8_O | Alcohol | | Upregulated | 34.1 | 14.59 | ppbv | (Monedeiro et al., 2021) |
|  |  |  |  |  |  | Altered | na | na | na | (Sakumura et al., 2017) |
|  |  |  |  |  |  | Upregulated | 4.37–13.15 | - | ppb | (Buszewski et al., 2012) |
|  |  |  |  |  |  | Downregulated | 7.14-57.81 | 38.46-103.63 | ng/L | (Koureas et al., 2020) |
| 5 | Butylated Hydroxytoluene | 128-37-0 | C_15_H_24_O | Alcohol | | Altered | na | na | na | (Y. Wang et al., 2012) |
|  |  |  |  |  |  | Altered | na | na | na | (Zou et al., 2014) |
| 6 | Ethanol | 64-17-5 | C_2_H_6_O | Alcohol | | Upregulated | −4.65^a^ | −619.49^a^ | ppb | (Filipiak et al., 2014) |
|  |  |  |  |  |  | Altered | na | na | na | (Sakumura et al., 2017) |
| 7 | Isopropanol | 67-63-0 | C_3_H_8_O | Alcohol | | Upregulated | -46.51^a^ | -393.56^a^ | ppb | (Filipiak et al., 2014) |
|  |  |  |  |  |  | Upregulated | 20-1007 | 19-725 | ppb | (Rudnicka et al., 2014) |
|  |  |  |  |  |  | Upregulated | 3.32–7.19 | 3.21–4.17 | ppb | (Buszewski et al., 2012) |
|  |  |  |  |  |  | Upregulated | 324-804 | 218-497 | ng/L | (Koureas et al., 2020) |
|  |  |  |  |  |  | Upregulated | 230.66 | 10.55 | ppbv | (Monedeiro et al., 2021) |
| 8 | Menthol | 89-78-1 | C_10_H_20_O | Alcohol | | Altered | na | na | na | (Pesesse et al., 2019) |
|  |  |  |  |  |  | Altered | na | na | na | (Zou et al., 2022) |
| 9 | Methanol | 67-56-1 | CH_4_O | Alcohol | | Downregulated | na | na | na | (Filipiak et al., 2014) |
|  |  |  |  |  |  | Altered | na | na | na | (Sakumura et al., 2017) |
| 10 | Phenol | 108-95-2 | C_6_H_6_OH | Alcohol | | Altered | na | na | na | (Chen et al., 2021) |
|  |  |  |  |  |  | Upregulated | 52.78 | nd | ppbv | (Monedeiro et al., 2021) |
|  |  |  |  |  |  | Altered | na | na | na | (M. Wang et al., 2018) |
| 11 | Phytol | 150-86-7 | C_20_H_40_O | Alcohol | | Altered | na | na | na | (Chen et al., 2021) |
|  |  |  |  |  |  | Altered | na | na | na | (Zou et al., 2022) |
|  |  |  |  |  |  | Altered | na | na | na | (M. Wang et al., 2018) |
| 12 | 2-Nonenal | 18829-56-6 | C_9_H_16_O | Aldehyde | | Altered | na | na | na | (Chen et al., 2021) |
|  |  |  |  |  |  | Upregulated | 2.6–9.9 | 1.8–5.0 | pM | (Corradi et al., 2015) |
| 13 | Acrolein | 107-02-8 | C_3_H_4_O | Aldehyde | | Upregulated | 6.84-94.36 | 5.10-9.57 | ppb | (Buszewski et al., 2012) |
|  |  |  |  |  |  | Upregulated | -0.13^a^ | -0.81^a^ | ppb | (Filipiak et al., 2014) |
| 14 | Butanal | 123-72-8 | C_4_H_8_O | Aldehyde | | Upregulated | 0.1–3.3 | 0.1–3.5 | ng/L | (Schallschmidt et al., 2016) |
|  |  |  |  |  |  | Upregulated | 1.32–2.55 | 1.35–1.87 | ppb | (Buszewski et al., 2012) |
| 15 | Decanal | 112-31-2 | C_10_H_20_O | Aldehyde | | Downregulated | na | na | na | (Filipiak et al., 2014) |
|  |  |  |  |  |  | Upregulated | 0.3–7.9 | 0.3–5.7 | ng/L | (Schallschmidt et al., 2016) |
|  |  |  |  |  |  | Altered | na | na | na | (M. Wang et al., 2018) |
| 16 | Heptanal | 111-71-7 | C_7_H_14_O | Aldehyde | | Altered | na | na | na | (Chen et al., 2021) |
|  |  |  |  |  |  | Upregulated | 10.4–21.3 | 8–20.3 | pM | (Corradi et al., 2015) |
| 17 | Hexadecanal | 629-80-1 | C_16_H_32_O | Aldehyde | | Altered | na | na | na | (Y. Wang et al., 2012) |
|  |  |  |  |  |  | Altered | na | na | na | (Zou et al., 2014) |
| 18 | Hexanal | 66-25-1 | C_6_H_12_O | Aldehyde | | Altered | na | na | na | (Chen et al., 2021) |
|  |  |  |  |  |  | Upregulated | na | na | na | (Filipiak et al., 2014) |
|  |  |  |  |  |  | Altered | 3.43-6.95 | 4.04-10.76 | ng/L | (Koureas et al., 2020) |
|  |  |  |  |  |  | Altered | na | na | na | (Rudnicka et al., 2019) |
|  |  |  |  |  |  | Upregulated | 0.5–60.5 | 0.3–27.3 | ng/L | (Schallschmidt et al., 2016) |
|  |  |  |  |  |  | Altered | na | na | na | (Gashimova et al., 2021) |
|  |  |  |  |  |  | Altered | na | na | na | (M. Wang et al., 2018) |
|  |  |  |  |  |  | Altered | na | na | na | (Zou et al., 2022) |
| 19 | Nonanal | 124-19-6 | C_9_H_18_O | Aldehyde | | Altered | 5.72-16.03 | 6.15-13.38 | ng/L | (Koureas et al., 2020) |
|  |  |  |  |  |  | Altered | 0.2–13.8 | 0.2–17.5 | ng/L | (Schallschmidt et al., 2016) |
|  |  |  |  |  |  | Altered | na | na | na | (Zou et al., 2022) |
|  |  |  |  |  |  | Altered | na | na | na | (M. Wang et al., 2018) |
| 20 | Octanal | 124-13-0 | C_8_H_16_O | Aldehyde | | Upregulated | 18–31 | 14–33 | pM | (Corradi et al., 2015) |
|  |  |  |  |  |  | Downregulated | na | na | na | (Filipiak et al., 2014) |
|  |  |  |  |  |  | Altered | na | na | na | (Gashimova et al., 2021) |
|  |  |  |  |  |  | Altered | 1.65-4.99 | 2.66-4.44 | ng/L | (Koureas et al., 2020) |
|  |  |  |  |  |  | Altered | 0.1–3.7 | 0.1–2.7 | ng/L | (Schallschmidt et al., 2016) |
|  |  |  |  |  |  | Altered | na | na | na | (Zou et al., 2021) |
|  |  |  |  |  |  | Altered | na | na | na | (Zou et al., 2022) |
|  |  |  |  |  |  | Altered | na | na | na | (M. Wang et al., 2018) |
| 21 | Propanal | 123-38-6 | C_3_H_6_O | Aldehyde | | Upregulated | 1.56-3.74 | 1.56-3.44 | ppb | (Buszewski et al., 2012) |
|  |  |  |  |  |  | Upregulated | -0.14^a^ | -7.13^a^ | ppb | (Filipiak et al., 2014) |
|  |  |  |  |  |  | Upregulated | 14.0 - 17.1 | 14.1 - 15.8 | ppb | (Ligor et al., 2015) |
|  |  |  |  |  |  | Upregulated | 24.2–179 | 3.1–214 | ng/L | (Schallschmidt et al., 2016) |
| 22 | 1,2,3-Trimethylbenzene | 526-73-8 | C_9_H_12_ | Aromatic hydrocarbon | | Altered | na | na | na | (Chen et al., 2021) |
|  |  |  |  |  |  | Altered | na | na | na | (M. Wang et al., 2018) |
|  |  |  |  |  |  | Altered | na | na | na | (Zou et al., 2021) |
| 23 | 1,2,4-Trimethylbenzene | 95-63-6 | C_9_H_12_ | Aromatic hydrocarbon | | Upregulated | 2.55 | 0.83 | ppbv | (Monedeiro et al., 2021) |
|  |  |  |  |  |  | Altered | na | na | na | (M. Wang et al., 2018) |
| 24 | 1-Ethyl-2,3-dimethylbenzene | 933-98-2 | C_10_H_14_ | Aromatic hydrocarbon | | Altered | na | na | na | (Chen et al., 2021) |
|  |  |  |  |  |  | Altered | na | na | na | (Zou et al., 2021) |
| 25 | 1-Methyl-3-Propylbenzene | 1074-43-7 | C_10_H_14_ | Aromatic hydrocarbon | | Altered | na | na | na | (Chen et al., 2021) |
|  |  |  |  |  |  | Altered | na | na | na | (M. Wang et al., 2018) |
| 26 | 2-Ethyl-p-xylene | 1758-88-9 | C_10_H_14_ | Aromatic hydrocarbon | | Altered | na | na | na | (Chen et al., 2021) |
|  |  |  |  |  |  | Altered | na | na | na | (M. Wang et al., 2018) |
| 27 | 3-Ethyltoluene | 620-14-4 | C_9_H_12_ | Aromatic hydrocarbon | | Altered | na | na | na | (M. Wang et al., 2018) |
|  |  |  |  |  |  | Altered | na | na | na | (Zou et al., 2021) |
|  |  |  |  |  |  | Altered | na | na | na | (Zou et al., 2022) |
| 28 | Benzene | 71-43 -2 | C_6_H_6_ | Aromatic hydrocarbon | | Upregulated | 1.29-3.82 | 1.38-14.97 | ppb | (Buszewski et al., 2012) |
|  |  |  |  |  |  | Altered | na | na | na | (Chen et al., 2021) |
|  |  |  |  |  |  | Altered | na | na | na | (Gashimova et al., 2021) |
|  |  |  |  |  |  | Altered | 0.66-3.17 | 1.21-5.15 | ng/L | (Koureas et al., 2020) |
|  |  |  |  |  |  | Upregulated | 0.1–38.4 | 0.1–38.2 | ng/L | (Schallschmidt et al., 2016) |
|  |  |  |  |  |  | Altered | na | na | na | (Zou et al., 2014) |
|  |  |  |  |  |  | Downregulated | na | na | na | (Koureas et al., 2021) |
| 29 | Cumene | 98-82-8 | C_9_H_12_ | Aromatic hydrocarbon | | Altered | na | na | na | (Chen et al., 2021) |
|  |  |  |  |  |  | Altered | na | na | na | (Zou et al., 2021) |
|  |  |  |  |  |  | Altered | na | na | na | (Zou et al., 2022) |
| 30 | Ethylbenzene | 100-41-4 | C_8_H_10_ | Aromatic hydrocarbon | | Altered | na | na | na | (Chen et al., 2021) |
|  |  |  |  |  |  | Altered | na | na | na | (Rudnicka et al., 2019) |
|  |  |  |  |  |  | Upregulated | 0.2–5.0 | 0.1–4.9 | ng/L | (Schallschmidt et al., 2016) |
|  |  |  |  |  |  | Upregulated | 5.8–26.3 | 4.3–15.3 | pM | (Corradi et al., 2015) |
|  |  |  |  |  |  | Upregulated | na | na | na | (Koureas et al., 2021) |
|  |  |  |  |  |  | Upregulated | 1.45–3.16 | 2.22–18.38 | ppb | (Buszewski et al., 2012) |
|  |  |  |  |  |  | Upregulated | 2.44-6.26 | 1.30-2.89 | ng/L | (Koureas et al., 2020) |
| 31 | o-Xylene | 95-47-6 | C_8_H_10_ | Aromatic hydrocarbon | | Altered | na | na | na | (Chen et al., 2021) |
|  |  |  |  |  |  | Altered | na | na | na | (Rudnicka et al., 2019) |
|  |  |  |  |  |  | Altered | na | na | na | (M. Wang et al., 2018) |
|  |  |  |  |  |  | Altered | na | na | na | (Zou et al., 2022) |
| 32 | Propylbenzene | 103-65-1 | C_9_H_12_ | Aromatic hydrocarbon | | Altered | na | na | na | (Chen et al., 2021) |
|  |  |  |  |  |  | Upregulated | 0.19–1.34 | 0–0.90 | ng/L | (Schallschmidt et al., 2016) |
|  |  |  |  |  |  | Altered | na | na | na | (M. Wang et al., 2018) |
| 33 | Styrene | 100-42-5 | C_8_H_8_ | Aromatic hydrocarbon | | Upregulated | 3.78 | 0.27 | ppbv | (Monedeiro et al., 2021) |
|  |  |  |  |  |  | Altered | na | na | na | (Zou et al., 2014) |
|  |  |  |  |  |  | Altered | na | na | na | (Zou et al., 2021) |
|  |  |  |  |  |  | Upregulated | na | na | na | (Koureas et al., 2021) |
|  |  |  |  |  |  | Upregulated | 2.36-7.87 | 1.25-3.53 | ng/L | (Koureas et al., 2020) |
|  |  |  |  |  |  | Downregulated | na | na | na | (Nardi-Agmon et al., 2016) |
| 34 | Toluene | 108-88-3 | C_7_H_8_ | Aromatic hydrocarbon | | Altered | na | na | na | (Gashimova et al., 2021) |
|  |  |  |  |  |  | Upregulated | na | na | na | (Koureas et al., 2021) |
|  |  |  |  |  |  | Upregulated | 15.35-66.04 | 18.14-51.17 | ng/L | (Koureas et al., 2020) |
| 35 | 2,4-Dimethylheptane | 2213-23-2 | C_9_H_20_ | Branched hydrocarbon | | Upregulated | 8.8 - 11.2 | 8.8-10.1 | ppb | (Ligor et al., 2015) |
|  |  |  |  |  |  | Altered | na | na | na | (Rudnicka et al., 2014) |
|  |  |  |  |  |  | Altered | na | na | na | (Zou et al., 2021) |
|  |  |  |  |  |  | Altered | na | na | na | (Rudnicka et al., 2019) |
| 36 | 2,6,11-Trimethyldodecane | 31295-56-4 | C_15_H_32_ | Branched hydrocarbon | | Altered | na | na | na | (Y. Wang et al., 2012) |
|  |  |  |  |  |  | Altered | na | na | na | (Zou et al., 2014) |
|  |  |  |  |  |  | Altered | na | na | na | (M. Wang et al., 2018) |
| 37 | 2-Methylpentane | 107-83-5 | C_6_H_14_ | Branched hydrocarbon | | Upregulated | 77–235 | 45–187 | pM | (Corradi et al., 2015) |
|  |  |  |  |  |  | Upregulated | na | na | na | (Filipiak et al., 2014) |
|  |  |  |  |  |  | Upregulated | 3.44 | 1.24 | ppbv | (Monedeiro et al., 2021) |
|  |  |  |  |  |  | Altered | 0.4–27 | 0.5–20.3 | ng/L | (Schallschmidt et al., 2016) |
|  |  |  |  |  |  | Altered | na | na | na | (Zou et al., 2014) |
| 38 | 3-Methylhexane | 589-34-4 | C_7_H_16_ | Branched hydrocarbon | | Upregulated | na | na | na | (Filipiak et al., 2014) |
|  |  |  |  |  |  | Altered | na | na | na | (Zou et al., 2021) |
| 39 | 3-Methylpentane | 96-14-0 | C_6_H_14_ | Branched hydrocarbon | | Altered | 0–7.2 | 0.3–7.7 | ng/L | (Schallschmidt et al., 2016) |
|  |  |  |  |  |  | Upregulated | 0.93 | 0.24 | ppbv | (Monedeiro et al., 2021) |
| 40 | 4-Methyloctane | 2216-34-4 | C_9_H_20_ | Branched hydrocarbon | | Altered | na | na | na | (Chen et al., 2021) |
|  |  |  |  |  |  | Altered | na | na | na | (Ligor et al., 2015) |
|  |  |  |  |  |  | Altered | na | na | na | (Rudnicka et al., 2019) |
| 41 | 5-Butylnonane | 17312-63-9 | C_13_H_28_ | Branched hydrocarbon | | Altered | na | na | na | (Y. Wang et al., 2012) |
|  |  |  |  |  |  | Altered | na | na | na | (M. Wang et al., 2018) |
| 42 | 8-Methylheptadecane | 13287-23-5 | C_18_H_38_ | Branched hydrocarbon | | Altered | na | na | na | (Chen et al., 2021) |
|  |  |  |  |  |  | Altered | na | na | na | (Y. Wang et al., 2012) |
|  |  |  |  |  |  | Altered | na | na | na | (M. Wang et al., 2018) |
| 43 | Isobutane | 75-28-5 | C_4_H_10_ | Branched hydrocarbon | | Altered | na | na | na | (Rudnicka et al., 2014) |
|  |  |  |  |  |  | Altered | na | na | na | (Rudnicka et al., 2019) |
| 44 | Pentadecane, 8-hexyl- | 13475-75-7 | C_21_H_44_ | Branched hydrocarbon | | Altered | na | na | na | (Y. Wang et al., 2012) |
|  |  |  |  |  |  | Altered | na | na | na | (Zou et al., 2014) |
| 45 | Pristane | 1921-70-6 | C_19_H_40_ | Branched hydrocarbon | | Altered | na | na | na | (Y. Wang et al., 2012) |
|  |  |  |  |  |  | Altered | na | na | na | (M. Wang et al., 2018) |
| 46 | (-)-β-Pinene | 18172-67-3 | C_10_H_16_ | Cyclic hydrocarbon | | Altered | na | na | na | (Rudnicka et al., 2019) |
|  |  |  |  |  |  | Altered | na | na | na | (Zou et al., 2021) |
| 47 | (+)-Longifolene | 475-20-7 | C_15_H_24_ | Cyclic hydrocarbon | | Altered | na | na | na | (Chen et al., 2021) |
|  |  |  |  |  |  | Altered | na | na | na | (M. Wang et al., 2018) |
| 48 | Cyclohexane | 110-82-7 | C_6_H_12_ | Cyclic hydrocarbon | | Altered | 0.43-2 | 0.43-2.48 | ng/L | (Koureas et al., 2020) |
|  |  |  |  |  |  | Upregulated | 0.4–24.8 | 0.4–19.0 | ng/L | (Schallschmidt et al., 2016) |
|  |  |  |  |  |  | Altered | na | na | na | (Rudnicka et al., 2019) |
| 49 | Limonene | 138-86-3 | C_10_H_16_ | Cyclic hydrocarbon | | Altered | na | na | na | (Rudnicka et al., 2019) |
|  |  |  |  |  |  | Altered | na | na | na | (Zou et al., 2022) |
|  |  |  |  |  |  | Upregulated | 1.87 | 1.57 | ppbv | (Monedeiro et al., 2021) |
| 50 | Methylcyclohexane | 108-87-2 | C_7_H_14_ | Cyclic hydrocarbon | | Altered | na | na | na | (Chen et al., 2021) |
|  |  |  |  |  |  | Altered | na | na | na | (M. Wang et al., 2018) |
| 51 | Propylcyclohexane | 1678-92-8 | C_9_H_18_ | Cyclic hydrocarbon | | Altered | na | na | na | (Chen et al., 2021) |
|  |  |  |  |  |  | Altered | na | na | na | (M. Wang et al., 2018) |
| 52 | Butyl acetate | 123-86-4 | C_6_H_12_O_2_ | Ester | | Altered | na | na | na | (Gashimova et al., 2021) |
|  |  |  |  |  |  | Altered | na | na | na | (Zou et al., 2022) |
| 53 | Ethyl acetate | 141-78-6 | C_4_H_8_O_2_ | Ester | | Altered | na | na | na | (Rudnicka et al., 2014) |
|  |  |  |  |  |  | Upregulated | 3.98 -22.89 | 1.12–8.22 | ppb | (Buszewski et al., 2012) |
|  |  |  |  |  |  | Altered | na | na | na | (Rudnicka et al., 2019) |
| 54 | Methyl acetate | 79-20-9 | C_3_H_6_O_2_ | Ester | | Downregulated | na | na | na | (Filipiak et al., 2014) |
|  |  |  |  |  |  | Altered | na | na | na | (Rudnicka et al., 2019) |
| 55 | Propyl acetate | 109-60-4 | C_5_H_10_O_2_ | Ester | | Altered | na | na | na | (Chen et al., 2021) |
|  |  |  |  |  |  | Altered | na | na | na | (Zou et al., 2021) |
|  |  |  |  |  |  | Altered | na | na | na | (Zou et al., 2022) |
| 56 | 2,5-Dimethylfuran | 625-86-5 | C_6_H_8_O | Ether | | Altered | na | na | na | (Chen et al., 2021) |
|  |  |  |  |  |  | Altered | na | na | na | (Zou et al., 2021) |
|  |  |  |  |  |  | Altered | na | na | na | (Zou et al., 2022) |
| 57 | Anethole | 104-46-1 | C_10_H_12_O | Ether | | Altered | na | na | na | (Chen et al., 2021) |
|  |  |  |  |  |  | Altered | na | na | na | (Pesesse et al., 2019) |
| 58 | Eucalyptol | 470-82-6 | C_10_H_18_O | Ether | | Altered | na | na | na | (Chen et al., 2021) |
|  |  |  |  |  |  | Altered | na | na | na | (Koureas et al., 2021) |
|  |  |  |  |  |  | Altered | na | na | na | (Zou et al., 2022) |
|  |  |  |  |  |  | Altered | na | na | na | (Monedeiro et al., 2021) |
| 59 | 1-Chlorooctadecane | 3386-33-2 | C_18_H_37_Cl | Halocompound | | Altered | na | na | na | (Chen et al., 2021) |
|  |  |  |  |  |  | Altered | na | na | na | (M. Wang et al., 2018) |
| 60 | Decane | 124-18-5 | C_10_H_22_ | Hydrocarbon (saturated) | | Altered | na | na | na | (Pesesse et al., 2019) |
|  |  |  |  |  |  | Altered | na | na | na | (Zou et al., 2014) |
|  |  |  |  |  |  | Altered | na | na | na | (Zou et al., 2021) |
| 61 | Dodecane | 112-40-3 | C_12_H_26_ | Hydrocarbon (saturated) | | Upregulated | na | na | na | (Filipiak et al., 2014) |
|  |  |  |  |  |  | Upregulated | 10.58 | 5.18 | ppbv | (Monedeiro et al., 2021) |
|  |  |  |  |  |  | Upregulated | 0.1–9.9 | 0.4–4.5 | ng/L | (Schallschmidt et al., 2016) |
|  |  |  |  |  |  | Altered | na | na | na | (M. Wang et al., 2018) |
|  |  |  |  |  |  | Altered | na | na | na | (Rudnicka et al., 2019) |
| 62 | Eicosane | 112-95-8 | C_20_H_42_ | Hydrocarbon (saturated) | | Altered | na | na | na | (Pesesse et al., 2019) |
|  |  |  |  |  |  | Altered | na | na | na | (Y. Wang et al., 2012) |
|  |  |  |  |  |  | Altered | na | na | na | (Zou et al., 2014) |
| 63 | Heptane | 142-82-5 | C_7_H_16_ | Hydrocarbon (saturated) | | Altered | na | na | na | (Chen et al., 2021) |
|  |  |  |  |  |  | Upregulated | 0.3–76.5 | 0–13.7 | ng/L | (Schallschmidt et al., 2016) |
|  |  |  |  |  |  | Altered | na | na | na | (Zou et al., 2022) |
|  |  |  |  |  |  | Altered | na | na | na | (M. Wang et al., 2018) |
| 64 | Hexane | 110-54-3 | C_6_H_14_ | Hydrocarbon (saturated) | | Upregulated | 23–77 | 9–50 | pM | (Corradi et al., 2015) |
|  |  |  |  |  |  | Upregulated | na | na | na | (Filipiak et al., 2014) |
|  |  |  |  |  |  | Altered | na | na | na | (Gashimova et al., 2021) |
|  |  |  |  |  |  | Upregulated | 0.4–72.3 | 0.3–3.0 | ng/L | (Schallschmidt et al., 2016) |
|  |  |  |  |  |  | Downregulated | 1.13-24.48 | 2.25-5.19 | ng/L | (Koureas et al., 2020) |
|  |  |  |  |  |  | Altered | na | na | na | (Rudnicka et al., 2019) |
| 65 | Nonadecane | 629-92-5 | C_19_H_40_ | Hydrocarbon (saturated) | | Altered | na | na | na | (Y. Wang et al., 2012) |
|  |  |  |  |  |  | Altered | na | na | na | (M. Wang et al., 2018) |
|  |  |  |  |  |  | Altered | na | na | na | (Zou et al., 2014) |
| 66 | Nonane | 111-84-2 | C_9_H_20_ | Hydrocarbon (saturated) | | Altered | na | na | na | (Chen et al., 2021) |
|  |  |  |  |  |  | Upregulated | 0.03^a^ | -0.01^a^ | ppb | (Filipiak et al., 2014) |
|  |  |  |  |  |  | Altered | na | na | na | (Rudnicka et al., 2019) |
|  |  |  |  |  |  | Altered | na | na | na | (Zou et al., 2022) |
| 67 | Octane | 111-65-9 | C_8_H_18_ | Hydrocarbon (saturated) | | Upregulated | 0.05^a^ | 0.02^a^ | ppb | (Filipiak et al., 2014) |
|  |  |  |  |  |  | Altered | 0.5-1.38 | 0.75-1.33 | ng/L | (Koureas et al., 2020) |
|  |  |  |  |  |  | Upregulated | 0.3–35.1 | 0.1–3.9 | ng/L | (Schallschmidt et al., 2016) |
| 68 | Pentadecane | 629-62-9 | C_15_H_32_ | Hydrocarbon (saturated) | | Altered | na | na | na | (Pesesse et al., 2019) |
|  |  |  |  |  |  | Altered | na | na | na | (M. Wang et al., 2018) |
| 69 | Pentane | 109-66-0 | C_5_H_12_ | Hydrocarbon (saturated) | | Upregulated | 290–1300 | 140–880 | pM | (Corradi et al., 2015) |
|  |  |  |  |  |  | Altered | 2.8–197 | 1.5–59.1 | ng/L | (Schallschmidt et al., 2016) |
| 70 | Propane | 74-98-6 | C_3_H_8_ | Hydrocarbon (saturated) | | Altered | na | na | na | (Ligor et al., 2015) |
|  |  |  |  |  |  | Altered | na | na | na | (Rudnicka et al., 2019) |
| 71 | Tetrapentacontane | 5856-66-6 | C_54_H_110_ | Hydrocarbon (saturated) | | Altered | na | na | na | (Chen et al., 2021) |
|  |  |  |  |  |  | Altered | na | na | na | (M. Wang et al., 2018) |
| 72 | Tridecane | 629-50-5 | C_13_H_28_ | Hydrocarbon (saturated) | | Upregulated | 42.16 | 3.43 | ppbv | (Monedeiro et al., 2021) |
|  |  |  |  |  |  | Altered | na | na | na | (Y. Wang et al., 2012) |
|  |  |  |  |  |  | Altered | na | na | na | (Zou et al., 2014) |
| 73 | Undecane | 1120-21-4 | C_11_H_24_ | Hydrocarbon (saturated) | | Altered | na | na | na | (Zou et al., 2022) |
|  |  |  |  |  |  | Upregulated | 3.83 | 0.8 | ppbv | (Monedeiro et al., 2021) |
| 74 | 1,4-Pentadiene | 591-93-5 | C_5_H_8_ | Hydrocarbon (unsaturated) | | Altered | na | na | na | (Rudnicka et al., 2014) |
|  |  |  |  |  |  | Altered | na | na | na | (Rudnicka et al., 2019) |
| 75 | Isoprene | 78-79-5 | C_5_H_8_ | Hydrocarbon (unsaturated) | | Altered | 1037-1986 | 1029-1952 | ng/L | (Koureas et al., 2020) |
|  |  |  |  |  |  | Altered | na | na | na | (Sakumura et al., 2017) |
|  |  |  |  |  |  | Altered | na | na | na | (Rudnicka et al., 2019) |
| 76 | Propylene | 115-07-1 | C_3_H_6_ | Hydrocarbon (unsaturated) | | Upregulated | na | na | na | (Filipiak et al., 2014) |
|  |  |  |  |  |  | Altered | na | na | na | (Ligor et al., 2015) |
| 77 | 2,3-Butanedione | 431-03-8 | C_4_H_6_O_2_ | Ketone | | Upregulated | na | na | na | (Filipiak et al., 2014) |
|  |  |  |  |  |  | Altered | na | na | na | (Rudnicka et al., 2019) |
| 78 | 2-Butanone | 78-93-3 | C_4_H_8_O | Ketone | | Altered | 3.03-6.9 | 3.27-7 | ng/L | (Koureas et al., 2020) |
|  |  |  |  |  |  | Upregulated | 1.93 | 1.74 | ppbv | (Monedeiro et al., 2021) |
|  |  |  |  |  |  | Upregulated | 3.4–179 | 2.4–58.2 | ng/L | (Schallschmidt et al., 2016) |
|  |  |  |  |  |  | Upregulated | 1.35–2.86 | 1.35–3.18 | ppb | (Buszewski et al., 2012) |
| 79 | 2-Pentanone | 107-87-9 | C_5_H_10_O | Ketone | | Altered | na | na | na | (Gashimova et al., 2021) |
|  |  |  |  |  |  | Upregulated | 7.1 - 29.2 | 7.1 - 16.9 | ppb | (Ligor et al., 2015) |
|  |  |  |  |  |  | Downregulated | 0.9–8.7 | 9.2–16.1 | ng/L | (Schallschmidt et al., 2016) |
|  |  |  |  |  |  | Upregulated | 3.25–8.77 | 1.80–4.11 | ppb | (Buszewski et al., 2012) |
|  |  |  |  |  |  | Altered | na | na | na | (Rudnicka et al., 2019) |
| 80 | 6-Methyl-5-heptene-2-one | 110-93-0 | C_8_H_14_O | Ketone | | Altered | na | na | na | (Pesesse et al., 2019) |
|  |  |  |  |  |  | Downregulated | na | na | na | (Filipiak et al., 2014) |
| 81 | Acetone | 67-64-1 | C_3_H_6_O | Ketone | | Upregulated | 34.57-390.60 | 44.20-531.45 | ppb | (Buszewski et al., 2012) |
|  |  |  |  |  |  | Altered | 3157-7921 | 2761-9580 | ng/L | (Koureas et al., 2020) |
|  |  |  |  |  |  | Altered | na | na | na | (Rudnicka et al., 2019) |
| 82 | Cyclohexanone | 108-94-1 | C_6_H_10_O | Ketone | | Altered | <0.48*-1.04 | 0.34-0.92 | ng/L | (Koureas et al., 2020) |
|  |  |  |  |  |  | Altered | na | na | na | (Rudnicka et al., 2019) |
| 83 | Methyl vinyl ketone | 78-94-4 | C_4_H_6_O | Ketone | | Altered | na | na | na | (Monedeiro et al., 2021) |
|  |  |  |  |  |  | Altered | na | na | na | (Rudnicka et al., 2019) |
| 84 | Benzothiazole | 95-16-9 | C_7_H_5_NS | Nitrogen and sulphur containing | | Altered | na | na | na | (Chen et al., 2021) |
|  |  |  |  |  |  | Altered | na | na | na | (M. Wang et al., 2018) |
| 85 | Acetonitrile | 75-05-8 | C_2_H_3_N | Nitrogen-containing | | Altered | na | na | na | (Monedeiro et al., 2021) |
|  |  |  |  |  |  | Altered | na | na | na | (Sakumura et al., 2017) |
|  |  |  |  |  |  | Altered | na | na | na | (Rudnicka et al., 2019) |
| 86 | Acetic acid | 64-19-7 | C_2_H_4_O_2_ | Organic acid | | Upregulated | na | na | na | (Filipiak et al., 2014) |
|  |  |  |  |  |  | Downregulated | na | na | na | (Koureas et al., 2021) |
|  |  |  |  |  |  | Altered | na | na | na | (Zou et al., 2022) |
| 87 | Propionic acid | 79-09-4 | C_3_H_6_O_2_ | Organic acid | | Downregulated | na | na | na | (Koureas et al., 2021) |
|  |  |  |  |  |  | Upregulated | na | na | na | (Muñoz-Lucas et al., 2020) |
| 88 | Dimethyl disulfide | 624-92-0 | C_2_H_6_S_2_ | Sulphur-containing | | Altered | na | na | na | (Gashimova et al., 2021) |
|  |  |  |  |  |  | Altered | na | na | na | (Zou et al., 2022) |
| 89 | Dimethyl sulﬁde | 75-18-3 | C_2_H_6_S | Sulphur-containing | | Upregulated | 5–110 | 5–79 | ppb | (Rudnicka et al., 2014) |
|  |  |  |  |  |  | Altered | na | na | na | (Rudnicka et al., 2019) |
| **Gastric cancer** | | | | | | | | | | |
| **Nº** | **Compound name** | **CAS-N** | **Formula** | **Chemical class** | | **Sign of alteration** | **Concentration (patients)** | **Concentration (controls)** | **Unit** | **Reference** |
| 1 | Nonanal | 124-19-6 | C_9_H_18_O | Aldehyde | | Upregulated | 157.30 ± 21.82 | 92.98 ± 5.47 | ppb | (Amal et al., 2013) |
|  |  |  |  |  |  | Downregulated | na | na | na | (Tong et al., 2017) |
| 2 | 2-Butoxyethanol | 111-76-2 | C_6_H_14_O_2_ | Ether | | Upregulated | 26.1 ± 4.3 | 24.9 ± 1.3 | ppb | (Amal, Leja, Funka, Skapars, et al., 2016) |
|  |  |  |  |  |  | Upregulated | 9.08 ± 0.32 | 9.12 ± 0.33 | ppbv | (Xu et al., 2013) |
| 3 | Furfural | 98-01-1 | C_5_H_4_O_2_ | Ether | | Upregulated | 5.6 ± 3.5 | 4.5 ± 0.9 | ppb | (Amal, Leja, Funka, Skapars, et al., 2016) |
|  |  |  |  |  |  | Upregulated | 2.32 ± 0.22 | 1.88 ± 0.18 | ppbv | (Xu et al., 2013) |
| 4 | Hexadecane | 544-76-3 | C_16_H_34_ | Hydrocarbon (saturated) | | Upregulated | na | na | na | (Tong et al., 2017) |
|  |  |  |  |  |  | Upregulated | 10.7 ± 12.3 | 4.2 ± 4.1 | ppb | (Amal, Leja, Funka, Skapars, et al., 2016) |
| 5 | 6-Methyl-5-hepten-2-one | 110-93-0 | C_8_H_14_O | Ketone | | Upregulated | 15.4 ± 1.12 | 11.42 ± 0.32 | ppb | (Amal et al., 2013) |
|  |  |  |  |  |  | Upregulated | 6.05 ± 1.18 | 4.12 ± 0.98 | ppbv | (Xu et al., 2013) |
|  |  |  |  |  |  | Upregulated | 70.3 ± 31.95 | 57.53 ± 25.20 | na | (Tong et al., 2017) |
| 6 | Acrylonitrile | 107-13-1 | C_3_H_3_N | Nitrogen-containing | | Upregulated | 13.2 ± 13.7 | 7.5 ± 6.2 | ppb | (Amal, Leja, Funka, Skapars, et al., 2016) |
|  |  |  |  |  |  | Upregulated | 4.24 ± 1.28 | 2.62 ± 0.57 | ppbv | (Xu et al., 2013) |
| **Colorectal cancer** | | | | | | | | | | |
| **Nº** | **Compound name** | **CAS-N** | **Formula** | | **Chemical class** | **Sign of alteration** | **Concentration (patients)** | **Concentration (controls)** | **Unit** | **Reference** |
| 1 | Ethanol | 64-17-5 | C_2_H_6_O | | Alcohol | Downregulated | 95.9 ± 48.1 | 464.7 ± 61.7 | ppb | (Amal, Leja, Funka, Lasina, et al., 2016) |
|  |  |  |  |  |  | Altered | na | na | na | (Altomare et al., 2020) |
| 2 | Decanal | 112-31-2 | C_10_H_20_O | | Aldehyde | Altered | na | na | na | (Altomare et al., 2015) |
|  |  |  |  |  |  | Altered | na | na | na | (Altomare et al., 2013) |
|  |  |  |  |  |  | Altered | na | na | na | (Altomare et al., 2020) |
| 3 | Nonanal | 124-19-6 | C_9_H_18_O | | Aldehyde | Altered | na | na | na | (Altomare et al., 2015) |
|  |  |  |  |  |  | Altered | na | na | na | (Altomare et al., 2013) |
|  |  |  |  |  |  | Altered | na | na | na | (Altomare et al., 2020) |
| 4 | m-Xylene | 108-38-3 | C_8_H_10_ | | Aromatic hydrocarbon | Altered | na | na | na | (Altomare et al., 2015) |
|  |  |  |  |  |  | Upregulated | na | na | na | (Altomare et al., 2013) |
| 5 | p-Xylene | 106-42-3 | C_8_H_10_ | | Aromatic hydrocarbon | Altered | na | na | na | (Altomare et al., 2015) |
|  |  |  |  |  |  | Altered | na | na | na | (Altomare et al., 2013) |
| 6 | 2-Methylpentane | 107-83-5 | C_6_H_14_ | | Branched hydrocarbon | Altered | na | na | na | (Altomare et al., 2015) |
|  |  |  |  |  |  | Altered | na | na | na | (Altomare et al., 2013) |
| 7 | 4-Methyloctane | 2216-34-4 | C_9_H_20_ | | Branched hydrocarbon | Downregulated | 16.0 ± 0.63 | 19.1 ± 0.8 | ppb | (Amal, Leja, Funka, Lasina, et al., 2016) |
|  |  |  |  |  |  | Upregulated | na | na | na | (Altomare et al., 2013) |
|  |  |  |  |  |  | Altered | na | na | na | (Altomare et al., 2020) |
| 8 | Isopentane | 78-78-4 | C_5_H_12_ | | Branched hydrocarbon | Altered | na | na | na | (Altomare et al., 2015) |
|  |  |  |  |  |  | Altered | na | na | na | (Altomare et al., 2013) |
| 9 | Cyclohexane | 110-82-7 | C_6_H_12_ | | Cyclic hydrocarbon | Altered | na | na | na | (Altomare et al., 2015) |
|  |  |  |  |  |  | Upregulated | na | na | na | (Altomare et al., 2013) |
| 10 | Methylcyclohexane | 108-87-2 | C_7_H_14_ | | Cyclic hydrocarbon | Altered | na | na | na | (Altomare et al., 2015) |
|  |  |  |  |  |  | Upregulated | na | na | na | (Altomare et al., 2013) |
| 11 | Methylcyclopentane | 96-37-7 | C_6_H_12_ | | Cyclic hydrocarbon | Altered | na | na | na | (Altomare et al., 2015) |
|  |  |  |  |  |  | Altered | na | na | na | (Altomare et al., 2013) |
| 12 | Dodecane | 112-40-3 | C_12_H_26_ | | Hydrocarbon (saturated) | Altered | na | na | na | (Altomare et al., 2015) |
|  |  |  |  |  |  | Upregulated | na | na | na | (C. Wang, Ke, et al., 2014) |
|  |  |  |  |  |  | Altered | na | na | na | (Altomare et al., 2020) |
| 13 | Undecane | 1120-21-4 | C_11_H_24_ | | Hydrocarbon (saturated) | Altered | na | na | na | (Altomare et al., 2015) |
|  |  |  |  |  |  | Altered | na | na | na | (Altomare et al., 2020) |
| 14 | 1,2-Pentadiene | 591-95-7 | C_5_H_8_ | | Hydrocarbon (unsaturated) | Altered | na | na | na | (Altomare et al., 2015) |
|  |  |  |  |  |  | Upregulated | na | na | na | (Altomare et al., 2013) |
| 15 | Methyl isobutyl ketone | 108-10-1 | C_6_H_12_O | | Ketone | Altered | na | na | na | (Altomare et al., 2015) |
|  |  |  |  |  |  | Altered | na | na | na | (Altomare et al., 2013) |
| **Breast cancer** | | | | | | | | | | |
| **Nº** | **Compound name** | **CAS-N** | **Formula** | | **Chemical class** | **Sign of alteration** | **Concentration (patients)** | **Concentration (controls)** | **Unit** | **Reference** |
| 1 | 2-Ethylhexan-1-ol | 104-76-7 | C_8_H_18_O | | Alcohol | Upregulated | 15.5-29.4 | 3.07 | ppb | (Barash et al., 2015) |
|  |  |  |  |  |  | Altered | na | na | na | (Zhang et al., 2020) |
| 2 | 2-Phenyl-2-propanol | 617-94-7 | C_9_H_12_O | | Alcohol | Downregulated | na | na | na | (C. Wang, Sun, et al., 2014) |
|  |  |  |  |  |  | Altered | na | na | na | (Zhang et al., 2020) |
| 3 | Hexanal | 66-25-1 | C_6_H_12_O | | Aldehyde | Upregulated | 0.104-0.591 | 0.014 | ppb | (Barash et al., 2015) |
|  |  |  |  |  |  | Upregulated | 34.49 ± 39.00 | 6.17 ± 6.78 | ppbv | (Li et al., 2014) |
| 4 | 2,3,4-Trimethylheptane | 52896-95-4 | C_10_H_22_ | | Branched hydrocarbon | Upregulated | <0.005*-0.05 | <0.005* | ppb | (Barash et al., 2015) |
|  |  |  |  |  |  | Downregulated | na | na | na | (C. Wang, Sun, et al., 2014) |
| 5 | Butyl acetate | 123-86-4 | C_6_H_12_O_2_ | | Ester | Upregulated | 0.591-2.27 | 0.109 | ppb | (Barash et al., 2015) |
|  |  |  |  |  |  | Downregulated | na | na | na | (C. Wang, Sun, et al., 2014) |
| 6 | Ethylene carbonate | 96-49-1 | C_3_H_4_O_3_ | | Ester | Downregulated | na | na | na | (C. Wang, Sun, et al., 2014) |
|  |  |  |  |  |  | Altered | na | na | na | (Zhang et al., 2020) |
| 7 | 2-Butoxyethanol | 111-76-2 | C_6_H_14_O_2_ | | Ether | Altered | na | na | na | (Zhang et al., 2020) |
|  |  |  |  |  |  | Upregulated | na | na | na | (C. Wang, Sun, et al., 2014) |
| 8 | Cyclohexanone | 108-94-1 | C_6_H_10_O | | Ketone | Upregulated | na | na | na | (C. Wang, Sun, et al., 2014) |
|  |  |  |  |  |  | Altered | na | na | na | (Zhang et al., 2020) |
| 9 | Cyclopentanone | 120-92-3 | C_5_H_8_O | | Ketone | Downregulated | na | na | na | (C. Wang, Sun, et al., 2014) |
|  |  |  |  |  |  | Altered | na | na | na | (Zhang et al., 2020) |
| 10 | Methacrylic Acid | 79-41-4 | C_4_H_6_O_2_ | | Organic acid | Altered | na | na | na | (Zhang et al., 2020) |
|  |  |  |  |  |  | Downregulated | na | na | na | (C. Wang, Sun, et al., 2014) |

| **Table S4**. Summary of group comparisons, statistical approaches and identified (VOC) in the studies focused on asthma, chronic obstructive pulmonary disease, obstructive sleep apnea and cystic fibrosis. *a* altered, *A-AD* asthma with other coexisting atopic diseases, *BS* asthma at baseline, *CF BL* cystic fibrosis at baseline, *CF* cystic fibrosis, *CF EA* cystic fibrosis acute exacerbation, *COPD (A)* chronic obstructive pulmonary disease acute exacerbation, *COPD (R)* chronic obstructive pulmonary disease recovery of exacerbation, *COPD (S)* chronic obstructive pulmonary disease stable, *COPD BL* chronic obstructive pulmonary disease at baseline, *COPD* chronic obstructive pulmonary disease , *down* downregulated, *EA* asthma exacerbation, *EO* eosinophilic asthma, *FEV1pp* percent-predicted forced expiratory volume in one second*, HC* healthy controls, *LC* lung cancer, *LCo* loss of asthma control, *MVA* multivariate analysis, *NA* non-asthma, *NA-AS* non-asthma with atopic diseases, *NA-NAD* non-asthma without atopic diseases, *NEU* neutrophilic asthma, *non-EA* asthma without exacerbation, *OSA* obstructive sleep apnea , *PAU* paucigranulocytic asthma, *PC* persistently controlled asthma, *PU* persistently uncontrolled asthma, *RC* recovery of asthma control, *TW* transient wheezers, *up* upregulated, *UVA* univariate analysis, *VC* variable asthma control. | | | | | |
| --- | --- | --- | --- | --- | --- |
| **Reference** | **Pathology** | **Comparison** | **Statistical approach** | **Significant VOCs** | **Details** |
| (Gahleitner et al., 2013) | Asthma | Asthma (n=11) vs HC (n=12) | UVA/MVA | 1-(Methylsulfanyl)propane (up), 1,4-Dichlorobenzene (up), 1,7-Dimethylnaphtalene (up), 1-Isopropyl-3-methylbenzene (up), 2-Octenal (up), 4-Isopropenyl-1-methylcyclohexene (up), Ethylbenzene (up), Octadecyne (up) |  |
| (Sola-Martínez et al., 2021) | Asthma | A-AD (n= 39) vs NA-AD (n=119) vs NA-NAD (n=178) vs NA (n=297) | MVA | 1,2-Benzenedicarboxylic acid, bis(2-methylpropyl) ester* (a), 2,2,4-Trimethyl-1,3-pentanediol diisobutyrate (a), 2-Ethyl-1-hexanol (a), 2-Propenoic acid, 3-(2-hydroxyphenyl)-, (E)-** (a), Acetone (a), Carbon disulfide (a), Decane* (a), Isoprene** (a), Tetrahydroisoquinoline derivative (a) | * Exclusive VOCs in models A-AD vs NA-AD; ** Exclusive VOCs in models NA-AD vs NA-NAD |
| (Schleich et al., 2019) | Asthma | Asthma (n=495): EO (n=212) vs NEO (n=103) vs PAU (n=180) | UVA/MVA | 1-Propanol*/** (down EO/up NEU), 2-Hexanone* (down), 3,7-Dimethylnonane** (up), 3-Tetradecene** (up), Hexane* / ** (down EO/up NEU), Nonanal** (up), Pentadecene** (up), Undecane** (down) | *Discriminant VOCs for EO; **Discriminant VOCs for NEU |
| (Brinkman et al., 2017) | Asthma | Asthma (n=23): BS (n=23) vs LCo (n=22) vs RC (n=22) | UVA/MVA | Acetonitrile (a), Bicyclo[2.2.2]octan-1-ol, 4-methyl (a), Methanol (a) |  |
| (Van Vliet et al., 2017) | Asthma | Asthma (n=94): EA (n=42) vs non-EA (n=49) | MVA | 1, 2-Dimethylcyclohexane (a), 2-Ethylhexanal (a), 2-Methylfuran / 3-Methylfuran (a), 6, 10-Dimethyl-5,9-undecadien-2-on (a), Nonanal (a), Octanal (a) |  |
| (Van Vliet et al., 2016) | Asthma | Asthma (n=96): PC (n=34) vs VC (n=53) vs PU (n=9) | MVA | 1,2-Dimethylcyclohexane (a), 2,4-Hexadiene (a), 2-Methylfuran (a), 3-Methylfuran (a), Butanoic acid (a), Dimethylsulfone (a), m-Cymene (a), Propylcyclohexane (a), Sulphurdioxide (a), Tetrachloroethylene (a) |  |
| (Meyer et al., 2014) | Asthma | Asthma (n=195) vs HC (n=40) | MVA | 1,3-Dioxolane, 2-(phenylmethyl)-* (up), 1-Dodecanol, 3,7,11-trimethyl-* (up), 2-Butyl-2,7-octadien-1-ol (down), 2-Propionyloxypentadecane (down), 4-Cyclopentene-1,3-dione, 4-phenyl- (up), 5-Hexenoic acid (down), Benzene (up), Dodecane (down), 2,4-dimethyl-heptane (down), Octanal (down), Phenol (down),decahydro-quinoline (down), Tetradecanoic acid (down) | *VOCs used for the cluster analysis |
| Smolinska et al., 2014) | Asthma | Asthma (n=76) vs TW (n=121) vs HC (n=49) | MVA | 1-Methyl-4-(1-methylethenyl) Cyclohexene (down), 2,2,4-Trimethylheptane (down), 2,3,6-Trimethyloctane (down), 2,4-Dimethylheptane (up), 2,4-Dimethylpentane (up), 2,6,10-Trimethyldodecane (down), 2-Ethynylnaphtalene (down), 2-Methylhexane (up), 2-Methylpentane (up), 2-Undecenal (up), Acetone (down), Biphenyl (down), Octane (up) |  |
| (Robroeks et al., 2013) | Asthma | Asthma (n=39): EA (n=16) vs non-EA (n=23) | UVA/MVA | 1-Phenyl-1-butene* (a), 2-Ethyl-1,3-butadiene** (a), 2-Ethyl-4-methyl-1-pentanol* (a), 2-Methyl-4H-1,3-benzoxathiine** (a), 2-Octen-1-ol** (a), 3-Methylpentane* (a), 4,6,9-Nonadecatriene* (a), Benzene** (a), Cyclohexane** (a), p-Xylene* (a) | *Discriminant VOCs of intrasubject comparison; **Discriminant VOCs of intersubject comparison |
| (Caldeira et al., 2012) | Asthma | Asthma (n=32) vs HC (n=27) | MVA | 1-Dodecene (a), 2,2,4,6,6-Pentamethylheptane* (a), 3,6-Dimethyldecane* (a), 6-Methyl-5-hepten-2-one (a), Decanal* (a), Decane* (a), Dodecanal * (a), Dodecane* (a), Nonanal* (a), Nonane* (a), Tetradecane* (a) | *VOCs used for the final model |
| (Monedeiro et al., 2021) | LC / COPD / Asthma | LC (n=16) vs COPD (n=12) vs asthma (n=8) vs HC (n=20) | UVA/MVA | 1-Pentanol (a), (E)-Ocimene* (up), 1,2,4-Trimethylbenzene (up), 1-Propanol (up), 2-Butanone (up), 2-Methyl-1-propanol (a), 2-Methyldecane (a), 2-Methylpentane (up), 2-Propanol* (up), 3,3-Dimethyl-butanamide (a), 3-Amino-butanoic acid (a), 3-Methylpentane* (up), Acetoin (up, down asthma), Acetonitrile (a), Benzonitrile* (up), Dodecane (up), Eucalyptol (a), Isododecane (up), Limonene* (up), m-Cymene* (up), Methyl vinyl ketone (a), Ocimene (a), Phenol* (up), Styrene (up), Terpineol* (up), Tetradecane (a), Tridecane (up), Undecane* (up) | *VOCs quantified and used to build the final model |
| (Pizzini et al., 2018) | COPD | COPD (A) (n=14) vs COPD (S) (n=16) vs HC (n=24) | UVA/MVA | 2,4-Dimethylheptane**** (down), 2,6-Dimethyloctane**** (down), 2-Methylhexane**** (up), 2-Pentanone* (up), 4-Heptanone* (up), 6-Methyl-5-hepten-2-on*** (up), Cyclohexane **** (up), Cyclohexanone* (up), Dimethyl disulfide*** (down), Methyl propyl sulfide** (down), n-Butane* (down), n-Heptane** (up) | *VOCs COPD (A) specific; ** VOCs COPD (S) specific; ***VOCs COPD (A) + COPD (S) specific; **** Differential VOCs COPD (A) vs HC and COPD (S) vs HC |
| (Basanta et al., 2012) | COPD | COPD (n=39) vs HC (n=32) | UVA/MVA | 1, 1'-Biphenyl,3-methyl** (a), 1,4-Methanoazulene, decahydro-4,8, 8-trimethyl-9-methylene-,[1S-(1α,3aβ,4α,8aβ)]*** (a), 2, 2, 4, 4-Tetramethyloctane*** (a), 3-Cyclohexen-1-ol,4-methyl-1-(1-methylethyl)-,acetate* (a), Benzofuran, 4, 5, 6, 7 tetrahydro-3,6-dimethyl* (a), Butanoic acid, 2,2-dimethyl-3-oxo-, ethyl ester (a),Cyclohexanol, 5-methyl-2-(1-methylethyl)-, [1R-(1α,2α,5β)]- (a), Decanal (a), Decane, 3-methyl* (a), Dodecanal (a), Furan,2-pentyl*** (a), Hexanal (a), Naphthalene, 2, 3,6-trimethyl*** (a), Nonanal (a), Oxirane, dodecyl (a), Pentadecanal (a), Pentanoic acid (a), Undecanal (a), Undecane, 3, 7-dimethyl *** (a), α-Methylstyrene* (a) | *VOCs associated with Eosinophils ≥ 1%; **VOCs associated with Eosinophils ≥ 2%; ***VOCs associated with EA ≥ 2/year |
| (Phillips et al., 2012) | COPD | COPD (n=119) vs HC (n=63) | MVA | 1,3,5,-Cycloheptatriene (a), 1-Heptene** (a), Acetic acid* (a), Benzaldehyde* (a), Benzene* (a), Butane (a), Butane, 2-methyl*** (a), Carbon dioxide* (a), Cyclopentanone*** (a), Decanal*** (a), Ethanethioamide*** (a), Hexanal (a), Isoprene* (a), Limonene*** (a), Nonadecane (a), Phenol (a), Phthalic anhydride (a), Sulphur dioxide* (a), Toluene (a) | *VOCs generated in the three comparisons; **VOCs generated in active vs former COPD smokers; ***VOCs generated in COPD vs HC never/former smokers |
| (van Velzen et al., 2019) | COPD | COPD BL (n=14) vs COPD (A) (n=14) vs COPD (R) (n=14) | UVA/MVA | (Z)-2-Decenal (a), 1,2-Pentadiene (a), 1-Undecanol (a), 4,7-Dimethyl-undecane (a), Acetone (a), Butyrolactone (a), Eicosane (a), Ethylbenzene (a), Limonene (a), Toluene (a) |  |
| (Gaida et al., 2016) | COPD | COPD (n=61) vs HC (n=58) | UVA/MVA | 1,6-Dimethyl-1,3,5-heptatriene* (up), 1-Ethyl-3-methyl benzene* (up), Acetic acid (a), Benzene* (up), Butanone (a), Indole* (up), Linalyl acetate (a), m,o,p-Xylene* (up), m/p-Cresol (a), o-Xylene (a), Phenol (a), Toluene* (up), Tridecane (a), Vinyl acetate* (up) | *VOCs in UVA |
| (Cazzola et al., 2015) | COPD | COPD (n=27) vs HC (n=7) | UVA | 1-Pentene,2,4,4-trimethyl (down), 2-Propanol (down), Benzene, 1,3,5-tri-tert-butyl (down), Butylated hydroxytoluene (down), Decane (up), Decane,6-ethyl-2-methyl (up), Hexane,3-ethyl-4-methyl (down), Hexyl ethylphosphonofluoridate (down), Limonene (down) |  |
| (Jareño-Esteban et al., 2017) | COPD | COPD (n=57) vs HC (n=100) | UVA | Hexanal (up), Nonanal* (up) | *Significant VOCs COPD vs HC never smokers |
| (Bayrakli et al. 2016) | OSA | OSA (n=10) vs HC (n=10) | UVA | Butanol |  |
| (Aoki et al. 2017) | OSA | OSA (n=41) vs HC (n=33) | UVA | Acetone*** (up), Decane** (up), Ethylbenzene*/** (up), Heptane (up), Hexane*** (up), Isoprene (up), Nonane* (up), Octane*** (up), Phenylacetic acid* (up), p-Xylene*/** (up), Toluene** (up) | *VOCs increased with OSA severity; *Significant VOCs in severe and most severe OSA; ***Significant VOCs in most severe OSA |
| (Woollam et al., 2022) | CF | CF BL (n=11) vs CF EA (n=7) | UVA | 2,4,4-Trimethyl-1,3-pentanediol 1-isobutyrate (up), 3,7-Dimethyldecane* (down), 5-Methyltridecane (down), Durene (down) | *VOC correlated with FEV1pp and ∆FEV1pp |
| (van Horck et al., 2021) | CF | CF (n=38) | MVA | 2,4-Dimethyl-1-heptene (a), 3-Dimethylbenzene and/or 1,4-Dimethylbenzene (m-/p-xylene) (a), 3-Methyl-2-butanone (a), Camphene (a), p-Benzoquinone (a), Pentadecane (a), Tetradecanal (a) |  |

**REFERENCES**

Altomare, D. F., Di Lena, M., Porcelli, F., Travaglio, E., Longobardi, F., Tutino, M., et al. (2015). Effects of curative colorectal cancer surgery on exhaled volatile organic compounds and potential implications in clinical follow-up. *Ann Surg* 262, 862–867. doi: 10.1097/SLA.0000000000001471.

Altomare, D. F., Di Lena, M., Porcelli, F., Trizio, L., Travaglio, E., Tutino, M., et al. (2013). Exhaled volatile organic compounds identify patients with colorectal cancer. *Br J Surg* 100, 144–150. doi: 10.1002/bjs.8942.

Altomare, D. F., Picciariello, A., Rotelli, M. T., De Fazio, M., Aresta, A., Zambonin, C. G., et al. (2020). Chemical signature of colorectal cancer: Case-control study for profiling the breath print. *BJS Open* 4, 1189–1199. doi: 10.1002/bjs5.50354.

Amal, H., Leja, M., Broza, Y. Y., Tisch, U., Funka, K., Liepniece-Karele, I., et al. (2013). Geographical variation in the exhaled volatile organic compounds. *J Breath Res* 7, 047102. doi: 10.1088/1752-7155/7/4/047102.

Amal, H., Leja, M., Funka, K., Lasina, I., Skapars, R., Sivins, A., et al. (2016a). Breath testing as potential colorectal cancer screening tool. *Int J Cancer* 138, 229–236. doi: 10.1002/ijc.29701.

Amal, H., Leja, M., Funka, K., Skapars, R., Sivins, A., Ancans, G., et al. (2016b). Detection of precancerous gastric lesions and gastric cancer through exhaled breath. *Gut* 65, 400–407. doi: 10.1136/gutjnl-2014-308536.

Aoki, T., Nagaoka, T., Kobayashi, N., Kurahashi, M., Tsuji, C., Takiguchi, H., et al. (2017). Prospective analyses of volatile organic compounds in obstructive sleep apnea patients. *Toxicol Sci* 156, 362–374. doi: 10.1093/toxsci/kfw260.

Barash, O., Zhang, W., Halpern, J. M., Hua, Q. L., Pan, Y. Y., Kayal, H., et al. (2015). Differentiation between genetic mutations of breast cancer by breath volatolomics. *Oncotarget* 6, 44864–44876. doi: 10.18632/oncotarget.6269.

Basanta, M., Ibrahim, B., Dockry, R., Douce, D., Morris, M., Singh, D., et al. (2012). Exhaled volatile organic compounds for phenotyping chronic obstructive pulmonary disease: A cross-sectional study. *Respir Res* 13, 72. doi: 10.1186/1465-9921-13-72.

Bayrakli, I., Öztürk, Ö., and Akman, H. (2016). Investigation of acetone, butanol and carbon dioxide as new breath biomarkers for convenient and noninvasive diagnosis of obstructive sleep apnea syndrome. *Biomed chromatogr* 30, 1890–1899. doi: 10.1002/bmc.3757.

Bhandari, M. P., Polaka, I., Vangravs, R., Mezmale, L., Veliks, V., Kirshners, A., et al. (2023). Volatile markers for cancer in exhaled breath—Could they be the signature of the gut microbiota? *Molecules* 28, 3488. doi: 10.3390/molecules28083488.

Brinkman, P., van de Pol, M. A., Gerritsen, M. G., Bos, L. D., Dekker, T., Smids, B. S., et al. (2017). Exhaled breath profiles in the monitoring of loss of control and clinical recovery in asthma. *Clin Exp Allergy* 47, 1159–1169. doi: 10.1111/cea.12965.

Buszewski, B., Ligor, T., Jezierski, T., Wenda-Piesik, A., Walczak, M., and Rudnicka, J. (2012). Identification of volatile lung cancer markers by gas chromatography-mass spectrometry: Comparison with discrimination by canines. *Anal Bioanal Chem* 404, 141–146. doi: 10.1007/s00216-012-6102-8.

Caldeira, M., Perestrelo, R., Barros, A. S., Bilelo, M. J., Morête, A., Câmara, J. S., et al. (2012). Allergic asthma exhaled breath metabolome: A challenge for comprehensive two-dimensional gas chromatography. *J Chromatogr A* 1254, 87–97. doi: 10.1016/j.chroma.2012.07.023.

Callol-Sanchez, L., Munoz-Lucas, M. A., Gomez-Martin, O., Maldonado-Sanz, J. A., Civera-Tejuca, C., Gutierrez-Ortega, C., et al. (2017). Observation of nonanoic acid and aldehydes in exhaled breath of patients with lung cancer. *J Breath Res* 11, 026004. doi: 10.1088/1752-7163/aa6485.

Cazzola, M., Segreti, A., Capuano, R., Bergamini, A., Martinelli, E., Calzetta, L., et al. (2015). Analysis of exhaled breath fingerprints and volatile organic compounds in COPD. *COPD Res Pract* 1, 7. doi: 10.1186/s40749-015-0010-1.

Chen, X., Muhammad, K. G., Madeeha, C., Fu, W., Xu, L., Hu, Y., et al. (2021). Calculated indices of volatile organic compounds (VOCs) in exhalation for lung cancer screening and early detection. *Lung Cancer* 154, 197–205. doi: 10.1016/j.lungcan.2021.02.006.

Corradi, M., Poli, D., Banda, I., Bonini, S., Mozzoni, P., Pinelli, S., et al. (2015). Exhaled breath analysis in suspected cases of non-small-cell lung cancer: A cross-sectional study. *J Breath Res* 9, 027101. doi: 10.1088/1752-7155/9/2/027101.

Filipiak, W., Filipiak, A., Sponring, A., Schmid, T., Zelger, B., Ager, C., et al. (2014). Comparative analyses of volatile organic compounds (VOCs) from patients, tumors and transformed cell lines for the validation of lung cancer-derived breath markers. *J Breath Res* 8, 027111. doi: 10.1088/1752-7155/8/2/027111.

Gahleitner, F., Guallar-Hoyas, C., Beardsmore, C. S., Pandya, H. C., and Thomas, C. P. (2013). Metabolomics pilot study to identify volatile organic compound markers of childhood asthma in exhaled breath. *Bioanalysis* 5, 2239–2247. doi: 10.4155/bio.13.184.

Gaida, A., Holz, O., Nell, C., Schuchardt, S., Lavae-Mokhtari, B., Kruse, L., et al. (2016). A dual center study to compare breath volatile organic compounds from smokers and non-smokers with and without COPD. *J Breath Res* 10, 026006. doi: 10.1088/1752-7155/10/2/026006.

Gashimova, E., Osipova, A., Temerdashev, A., Porkhanov, V., Polyakov, I., Perunov, D., et al. (2021). Exhaled breath analysis using GC-MS and an electronic nose for lung cancer diagnostics. *Anal Methods* 13, 4793–4804. doi: 10.1039/d1ay01163d.

Jareño-Esteban, J. J., Muñoz-Lucas, M. Á., Gómez-Martín, Ó., Utrilla-Trigo, S., Gutiérrez-Ortega, C., Aguilar-Ros, A., et al. (2017). Study of 5 volatile organic compounds in exhaled breath in chronic obstructive pulmonary disease. *Arch Bronconeumol* 53, 251–256. doi: 10.1016/j.arbres.2016.09.003.

Koureas, M., Kalompatsios, D., Amoutzias, G. D., Hadjichristodoulou, C., Gourgoulianis, K., and Tsakalof, A. (2021). Comparison of targeted and untargeted approaches in breath analysis for the discrimination of lung cancer from benign pulmonary diseases and healthy persons. *Molecules* 26, 2609. doi: 10.3390/molecules26092609.

Koureas, M., Kirgou, P., Amoutzias, G., Hadjichristodoulou, C., Gourgoulianis, K., and Tsakalof, A. (2020). Target analysis of volatile organic compounds in exhaled breath for lung cancer discrimination from other pulmonary diseases and healthy persons. *Metabolites* 10, 317. doi: 10.3390/metabo10080317.

Li, J., Peng, Y., Liu, Y., Li, W., Jin, Y., Tang, Z., et al. (2014). Investigation of potential breath biomarkers for the early diagnosis of breast cancer using gas chromatography-mass spectrometry. *Clin Chim Acta* 436, 59–67. doi: 10.1016/j.cca.2014.04.030.

Ligor, T., Pater, Ł., and Buszewski, B. (2015). Application of an artificial neural network model for selection of potential lung cancer biomarkers. *J Breath Res* 9, 027106. doi: 10.1088/1752-7155/9/2/027106.

Meyer, N., Dallinga, J. W., Nuss, S. J., Moonen, E. J. C., van Berkel, J. J. B. N., Akdis, C., et al. (2014). Defining adult asthma endotypes by clinical features and patterns of volatile organic compounds in exhaled air. *Respir Res* 15, 136. doi: 10.1186/S12931-014-0136-8.

Monedeiro, F., Monedeiro-Milanowski, M., Ratiu, I. A., Brożek, B., Ligor, T., and Buszewski, B. (2021). Needle trap device-GC-MS for characterization of lung diseases based on breath VOC profiles. *Molecules* 26, 1789. doi: 10.3390/molecules26061789.

Muñoz-Lucas, M. Á., Jareño-Esteban, J., Gutiérrez-Ortega, C., López-Guijarro, P., Collado-Yurrita, L., Quintana-Díaz, M., et al. (2020). Influence of chronic obstructive pulmonary disease on volatile organic compounds in patients with non-small cell lung cancer. *Arch Bronconeumol* 56, 801–805. doi: 10.1016/j.arbr.2020.10.004.

Nardi-Agmon, I., Abud-Hawa, M., Liran, O., Gai-Mor, N., Ilouze, M., Onn, A., et al. (2016). Exhaled breath analysis for monitoring response to treatment in advanced lung cancer. *J Thorac Oncol* 11, 827–837. doi: 10.1016/j.jtho.2016.02.017.

Pesesse, R., Stefanuto, P. H., Schleich, F., Louis, R., and Focant, J. F. (2019). Multimodal chemometric approach for the analysis of human exhaled breath in lung cancer patients by TD-GC × GC-TOFMS. *J Chromatogr B* 1114–1115, 146–153. doi: 10.1016/j.jchromb.2019.01.029.

Phillips, C. O., Syed, Y., Parthaláin, N. Mac, Zwiggelaar, R., Claypole, T. C., and Lewis, K. E. (2012). Machine learning methods on exhaled volatile organic compounds for distinguishing COPD patients from healthy controls. *J Breath Res* 6, 036003. doi: 10.1088/1752-7155/6/3/036003.

Pizzini, A., Filipiak, W., Wille, J., Ager, C., Wiesenhofer, H., Kubinec, R., et al. (2018). Analysis of volatile organic compounds in the breath of patients with stable or acute exacerbation of chronic obstructive pulmonary disease. *J Breath Res* 12, 036002. doi: 10.1088/1752-7163/aaa4c5.

Robroeks, C. M., Van Berkel, J. J., Jöbsis, Q., Van Schooten, F. J., Dallinga, J. W., Wouters, E. F., et al. (2013). Exhaled volatile organic compounds predict exacerbations of childhood asthma in a 1-year prospective study. *Eur Respir J* 42, 98–106. doi: 10.1183/09031936.00010712.

Rudnicka, J., Kowalkowski, T., and Buszewski, B. (2019). Searching for selected VOCs in human breath samples as potential markers of lung cancer. *Lung Cancer* 135, 123–129. doi: 10.1016/j.lungcan.2019.02.012.

Rudnicka, J., Walczak, M., Kowalkowski, T., Jezierski, T., and Buszewski, B. (2014). Determination of volatile organic compounds as potential markers of lung cancer by gas chromatography-mass spectrometry versus trained dogs. *Sens Actuators B Chem* 202, 615–621. doi: 10.1016/j.snb.2014.06.006.

Saidi, T., Moufid, M., de Jesus Beleño-Saenz, K., Welearegay, T. G., El Bari, N., Lisset Jaimes-Mogollon, A., et al. (2020). Non-invasive prediction of lung cancer histological types through exhaled breath analysis by UV-irradiated electronic nose and GC/QTOF/MS. *Sens Actuators B Chem* 311, 127932. doi: 10.1016/j.snb.2020.127932.

Sakumura, Y., Koyama, Y., Tokutake, H., Hida, T., Sato, K., Itoh, T., et al. (2017). Diagnosis by volatile organic compounds in exhaled breath from lung cancer patients using support vector machine algorithm. *Sensors (Basel)* 17, 287. doi: 10.3390/s17020287.

Schallschmidt, K., Becker, R., Jung, C., Bremser, W., Walles, T., Neudecker, J., et al. (2016). Comparison of volatile organic compounds from lung cancer patients and healthy controls - Challenges and limitations of an observational study. *J Breath Res* 10, 046007. doi: 10.1088/1752-7155/10/4/046007.

Schleich, F. N., Zanella, D., Stefanuto, P. H., Bessonov, K., Smolinska, A., Dallinga, J. W., et al. (2019). Exhaled volatile organic compounds are able to discriminate between neutrophilic and eosinophilic asthma. *Am J Respir Crit Care Med* 200, 444–453. doi: 10.1164/rccm.201811-2210OC.

Smolinska, A., Klaassen, E. M. M., Dallinga, J. W., Van De Kant, K. D. G., Jobsis, Q., Moonen, E. J. C., et al. (2014). Profiling of volatile organic compounds in exhaled breath as a strategy to find early predictive signatures of asthma in children. *PLoS One* 9, e95668. doi: 10.1371/journal.pone.0095668.

Sola-Martínez, R. A., Lozano-Terol, G., Gallego-Jara, J., Morales, E., Cantero-Cano, E., Sánchez-Solís de Querol, M., et al. (2021). Exhaled volatilome analysis as a useful tool to discriminate asthma with other coexisting atopic diseases in women of childbearing age. *Sci Rep* 11, 13823. doi: 10.1038/S41598-021-92933-2.

Tong, H., Wang, Y., Li, Y., Liu, S., Chi, C., Liu, D., et al. (2017). Volatile organic metabolites identify patients with gastric carcinoma, gastric ulcer, or gastritis and control patients. *Cancer Cell Int* 17, 108. doi: 10.1186/s12935-017-0475-x.

van Horck, M., Smolinska, A., Wesseling, G., de Winter-De Groot, K., de Vreede, I., Winkens, B., et al. (2021). Exhaled volatile organic compounds detect pulmonary exacerbations early in children with cystic fibrosis: Results of a 1 year observational pilot study. *J Breath Res* 15, 026012. doi: 10.1088/1752-7163/abda55.

van Velzen, P., Brinkman, P., Knobel, H. H., van den Berg, J. W. K., Jonkers, R. E., Loijmans, R. J., et al. (2019). Exhaled breath profiles before, during and after exacerbation of COPD: A prospective follow-up study. *COPD* 16, 330–337. doi: 10.1080/15412555.2019.1669550.

Van Vliet, D., Smolinska, A., Jöbsis, Q., Rosias, P., Muris, J., Dallinga, J., et al. (2017). Can exhaled volatile organic compounds predict asthma exacerbations in children? *J Breath Res* 11, 016016. doi: 10.1088/1752-7163/aa5a8b.

Van Vliet, D., Smolinska, A., Jöbsis, Q., Rosias, P. P. R., Muris, J. W. M., Dallinga, J. W., et al. (2016). Association between exhaled inflammatory markers and asthma control in children. *J Breath Res* 10, 016014. doi: 10.1088/1752-7155/10/1/016014.

Wang, C., Ke, C., Wang, X., Chi, C., Guo, L., Luo, S., et al. (2014a). Noninvasive detection of colorectal cancer by analysis of exhaled breath. *Anal Bioanal Chem* 406, 4757–4763. doi: 10.1007/s00216-014-7865-x.

Wang, C., Sun, B., Guo, L., Wang, X., Ke, C., Liu, S., et al. (2014b). Volatile organic metabolites identify patients with breast cancer, cyclomastopathy, and mammary gland fibroma. *Sci Rep* 4, 5383. doi: 10.1038/srep05383.

Wang, M., Sheng, J., Wu, Q., Zou, Y., Hu, Y., Ying, K., et al. (2018). Confounding effect of benign pulmonary diseases in selecting volatile organic compounds as markers of lung cancer. *J Breath Res* 12, 046013. doi: 10.1088/1752-7163/aad9cc.

Wang, Y., Hu, Y., Wang, D., Yu, K., Wang, L., Zou, Y., et al. (2012). The analysis of volatile organic compounds biomarkers for lung cancer in exhaled breath, tissues and cell lines. *Cancer Biomarkers* 11, 129–137. doi: 10.3233/CBM-2012-0270.

Woollam, M., Siegel, A. P., Grocki, P., Saunders, J. L., Sanders, D. B., Agarwal, M., et al. (2022). Preliminary method for profiling volatile organic compounds in breath that correlate with pulmonary function and other clinical traits of subjects diagnosed with cystic fibrosis: A pilot study. *J Breath Res* 16, 027103. doi: 10.1088/1752-7163/ac522f.

Xu, Z. Q., Broza, Y. Y., Ionsecu, R., Tisch, U., Ding, L., Liu, H., et al. (2013). A nanomaterial-based breath test for distinguishing gastric cancer from benign gastric conditions. *Br J Cancer* 108, 941–950. doi: 10.1038/bjc.2013.44.

Zhang, Y., Guo, L., Qiu, Z., Lv, Y., Chen, G., and Li, E. (2020). Early diagnosis of breast cancer from exhaled breath by gas chromatography-mass spectrometry (GC/MS) analysis: A prospective cohort study. *J Clin Lab Anal* 34, e23526. doi: 10.1002/jcla.23526.

Zou, Y., Hu, Y., Jiang, Z., Chen, Y., Zhou, Y., Wang, Z., et al. (2022). Exhaled metabolic markers and relevant dysregulated pathways of lung cancer: A pilot study. *Ann Med* 54, 790–802. doi: 10.1080/07853890.2022.2048064.

Zou, Y., Wang, Y., Jiang, Z., Zhou, Y., Chen, Y., Hu, Y., et al. (2021). Breath profile as composite biomarkers for lung cancer diagnosis. *Lung Cancer* 154, 206–213. doi: 10.1016/j.lungcan.2021.01.020.

Zou, Y., Zhang, X., Chen, X., Hu, Y., Ying, K., and Wang, P. (2014). Optimization of volatile markers of lung cancer to exclude interferences of non-malignant disease. *Cancer Biomarkers* 14, 371–379. doi: 10.3233/CBM-140418.
